# Variational Bayesian Approximation (VBA): Implementation and Comparison of Different Optimization Algorithms

**DOI:** 10.3390/e26080707

**Published:** 2024-08-20

**Authors:** Seyedeh Azadeh Fallah Mortezanejad, Ali Mohammad-Djafari

**Affiliations:** 1 School of Automotive and Traffic Engineering, Jiangsu University, Zhenjiang 212013, China; azadehfallah131@gmail.com; 2 International Science Consulting and Training (ISCT), 91440 Bures sur Yvette, France; 3 Shanfeng Company, Shaoxing 312352, China

**Keywords:** variational Bayesian approach (VBA), Kullback–Leibler divergence (KLD), mean field approximation (MFA), optimization algorithm

## Abstract

In any Bayesian computations, the first step is to derive the joint distribution of all the unknown variables given the observed data. Then, we have to do the computations. There are four general methods for performing computations: Joint MAP optimization; Posterior expectation computations that require integration methods; Sampling-based methods, such as MCMC, slice sampling, nested sampling, etc., for generating samples and numerically computing expectations; and finally, Variational Bayesian Approximation (VBA). In this last method, which is the focus of this paper, the objective is to search for an approximation for the joint posterior with a simpler one that allows for analytical computations. The main tool in VBA is to use the Kullback–Leibler Divergence (KLD) as a criterion to obtain that approximation. Even if, theoretically, this can be conducted formally, for practical reasons, we consider the case where the joint distribution is in the exponential family, and so is its approximation. In this case, the KLD becomes a function of the usual parameters or the natural parameters of the exponential family, where the problem becomes parametric optimization. Thus, we compare four optimization algorithms: general alternate functional optimization; parametric gradient-based with the normal and natural parameters; and the natural gradient algorithm. We then study their relative performances on three examples to demonstrate the implementation of each algorithm and their efficiency performance.

## 1. Introduction

The Bayesian inference starts by defining the joint posterior distribution of all the unknowns in the models given the data. Then, to obtain a point estimation, there are two main solutions: Joint Maximum A Posterior (JMAP), and computation of the posterior expectations. JMAP requires optimization to determine the unknown parameters that maximize the joint posterior distribution. Computing posterior expectations requires integration and involves calculating the expected values of unknown parameters. These computations are possible analytically in the simple case of linear and Normal priors or for exponential families with conjugate priors. In all other cases, the computation of multivariate integration is too costly. Then, there are two main tools. First, sampling methods such as Markov Chain Monte Carlo (MCMC), slice sampling, nested sampling, etc., where we generate samples and then compute means, variances, covariances, etc. Second, analytic approximation methods, which first approximate the original posterior with a simpler one to facilitate analytical computations. The Variational Bayesian Approximation (VBA) is the main method of this kind, using the KLD divergence as the criterion to obtain such an approximation. However, even if theoretically, the functional optimization of KLD is possible, it still needs integral computations, which are possible if we have some conjugacy properties. In particular, when the original posterior expression is in the exponential families, the approximation expression is also in the exponential families thanks to the KLD properties. In this paper, we consider this case and accomplish the optimization task in various ways. The first approach involves directly applying alternate optimization to the KLD criterion. Other methods consist of expressing the KLD as a function of parameters and using gradient-based algorithms. As the exponential families can be represented using normal or natural parameters, we obtain different algorithms that we compare.

For a general Bayesian inference, the main computation tool is the exploration of the joint posterior law via the sampling methods, such as the MCMC, or other more recent techniques such as slice sampling [1] or nested sampling [2]. However, these methods become very costly for high dimensional problems in Computer Vision, Machine Learning, and Artificial Intelligence. Blei et al. (2017) [3] mentioned in their review article the alternative of MCMC with the support of variational inference. They found Bayesian inference fast and easy to scale up to large data sets. Variational inference has less complexity than MCMC in many examples, such as large-scale document analysis, computational neuroscience, and Computer Vision.

The VBA is constructed for a variety of models to approximate a posterior distribution with many variables whose aim is to minimize the computational costs while keeping controllable accuracy. The main reduction in computational cost is achieved by searching for a good and tractable approximation for the joint posterior. VBA has acceptable efficiency computational costs compared with sampling methods.

Parisi and Shankar [4] have been a pioneer in VBA since 1988, and after that MacKay and Neal [5,6] were forerunners in Bayesian Neural Network. Šmídl and Quinn [7] provided a theoretical background of VBA and its iterative algorithms applied in signal processing. Sarkka and Nummenmaa [8] used VBA on an autoregressive model with unknown variance, which is an application of VBA in signal processing. Zheng et al. [9] introduced a new version of VBA for image restoration concerning an ill-posed linear inverse problem applying all variation priors. Their method structure was on the memory transposition subspace algorithm for a probability density function space. Many other authors used this approach for different applications [7,10,11,12,13]. Renard et al. [14] worked on a Bayesian inference application for regional frequency analysis in a nonstationary context to handle intricate models getting physical reality and statistical necessities. Li and Shi [15] explained the matter of the model accuracy in the frameworks of wind power. Bayesian approaches have many advantages in the uncertainty and variability estimations, which are crucial in model prediction. They mentioned Bayesian methods being appropriate for various aspects of wind energy conversion systems, such as enhancing the precision and reliability of wind resource estimation and short-term predictions. Bayesian models have applications on enormous data of microarray technology, which are challenging in making inferences from such massive data sets. Yang et al. [16] introduced Bayesian methods as popular techniques for their benefits in microarray analysis.

In many applications, with indirect observations, such as using a thermometer sensor to measure the temperature (hidden variable), Bayesian inference starts with obtaining the expression of the joint distribution of all the unknown variables (hidden variables and parameters of the model) given the observed data. Then, we have to use it to do inference. In general, this expression is not separable in all its variables. So, the computations become hard and costly. For example, obtaining the marginals for each variable and computing the expectations are difficult and expensive. This problem becomes even more crucial in high-dimensional Computer Vision and Machine Learning and becomes an issue in inverse problems. We may then need to propose a surrogate expression with which we can do approximate computations.

The VBA is a technique that approximates the joint distribution *p* with an easier method; for example, a separable one *q* by minimizing KL(q|p), which makes the marginal computations much easier. For example, in the case of two variables, p(x,y) is approximated by $q\left(x,y\right)=q_{1}\left(x\right)q_{2}\left(y\right)$ via minimizing $KL(q_{1}q_{2}|p)$. When *q* is separable in all the variables of *p*, the approximation is also called Mean Field Approximation (MFA).

To obtain the approximate marginals $q_{1}$ and $q_{2}$, we must minimize $KL(q_{1}q_{2}|p)$. A first standard and general algorithm is the alternate analytic optimization of $KL(q_{1}q_{2}|p)$ with respect to $q_{1}$, and then, to $q_{2}$. Finding the expression of the functional derivatives of $KL(q_{1}q_{2}|p)$ for $q_{1}$ and $q_{2}$ and equating them to zero alternatively, we obtain an iterative optimization algorithm. A second general approach is its optimization in the Riemannian Manifold, where we consider the case *p* is in the exponential family and so are $q_{1}$ and $q_{2}$. For this case, $KL(q_{1}q_{2}|p)$ becomes a function of the parameters θ of the exponential family. Then, we can use any other optimization algorithm to obtain those parameters.

In this paper, we review the VBA technique and then compare four different VBA optimization algorithms in three examples: standard alternate analytic optimization, a gradient-based algorithm for normal and natural parameter space [17,18] and a natural gradient algorithm [19,20,21] The paper aims to consider the first algorithm as our principal method and compare it with the three other algorithms. Building upon our initial proceedings paper [22], presented at the 41st International Workshop, on Bayesian inference and maximum entropy methods in 2022, this journal article further investigates more theoretical details, algorithms, and comparison study.

In this article, we consider the case of exponential families, so that *p* and *q* are both in exponential families. Then, we write the expression of the KL(q|p) and explore four different estimation algorithms for unknown parameters in a model that incorporates prior information. The first iterative algorithm handles directly KL(·), alternatively optimizing it with respect to each marginal $q_{i}$. This is the standard optimization used often in VBA. The function to be optimized is KLD. First, the gradient of KLD for all unknown parameters needs to be found. Then, we can begin with initial values for the parameters, either estimated from data or chosen deliberately. Then, we repeat the iterative algorithm until it converges to some points. If we denote the unknown normal parameter space with θ, then the iterative optimization algorithm can be written as: ${\tilde{\theta }}^{\left(k+1\right)}={\tilde{\theta }}^{\left(k\right)}-\gamma \nabla KL\left({\tilde{\theta }}^{\left(k\right)}\right)$ for gradient-based algorithm and the same with the natural parameters ${\tilde{\Lambda }}^{\left(k+1\right)}={\tilde{\Lambda }}^{\left(k\right)}-\gamma \nabla KL\left({\tilde{\Lambda }}^{\left(k\right)}\right)$ with different values of γ. The natural gradient definition, mentioned by Amari [23], is:

(1) $$\tilde{\nabla }h=F^{-1}\nabla h,$$

where *F* and *h* are the Fisher information matrix and objective function, respectively. In our case, *h* is the KLD. To make Fisher’s formula understandable, we change p(x,y) to p(x,y|θ) to explicitly show the parameters p(x,y), and differentiate for θ. The Fisher information matrix of p(x,y|θ) is given by:

(2) $$\begin{array}{cc} F= & {\langle \nabla \ln p\left(x,y|\theta \right)\nabla \ln p{\left(x,y|\theta \right)}^{⊺}\rangle }_{p(x,y)}\\ = & -{\langle H_{\ln p(x,y|\theta )}\rangle }_{p(x,y)}, \end{array}$$

where $H_{\ln p(x,y|\theta )}$ and ${\langle \cdot \rangle }_{p(x,y)}$ are the Hessian matrix of lnp(x,y|θ) concerning θ and the expectation respect to p(x,y) distribution, respectively. An approximation of the Fisher information, introduced by Schraudolph [24], is called Empirical Fisher:

$$\begin{array}{cc} \bar{F}= & {\langle \nabla \ln p\left(x,y|\theta \right)\nabla \ln p{\left(x,y|\theta \right)}^{⊺}\rangle }_{q(x,y)}\\ = & \frac{1}{∥S_{XY}∥}\sum_{\left(x,y\right)\in S_{XY}}\nabla \ln p\left(y|x,\theta \right)\nabla \ln p{\left(y|x,\theta \right)}^{⊺}. \end{array}$$

The fundamental natural gradient iteration algorithm is:

(3) $${\tilde{\theta }}^{\left(k+1\right)}={\tilde{\theta }}^{\left(k\right)}-\rho_{k}\tilde{\nabla }h,$$

where $\rho_{k}$ is a learning-rate schedule. Martens [25] suggested an optimum update based on a particular second-order local approximation of *h*, $\rho_{k}=1$.

We consider three examples: Normal-Inverse-Gamma, multivariate Normal, and linear inverse problems to assess the performance and convergence speed of the algorithms through multiple simulations. We propose the following organization of this paper: In Section 2, we present a brief explanation of the basic VBA analytical alternate optimization algorithm. In Section 3, we illustrate our first example related to Normal-Inverse-Gamma distribution analytically and, in practice, explain the outcomes of four algorithms to estimate the unknown parameters. In Section 4, we study a more complex example of a multivariate Normal distribution whose means and variance-covariance matrix are unknown and have a Normal-Inverse-Wishart distribution. This section aims to demonstrate marginal distributions of $\tilde{\mu }$ and $\tilde{\Sigma }$ using a set of multivariate Normal observations using these mean and variance. In Section 5, the example is closest to realistic situations and is a linear inverse problem. We simulate the model with different dimensions to see the changes in the performance of the algorithms. In Section 6, we present our work summary in the article and compare the four recursive algorithms in three different examples.

## 2. Variational Bayesian Approach (VBA)

In this paper, our focus is on MFA as a subset of VBA. In MFA, the posterior distribution p(x,y) is approximated by separable q(x,y):

$$q\left(x,y\right)=q_{1}\left(x\right)q_{2}\left(y\right).$$

KL(q|p) [26] is an information measure of discrepancy between two probability functions and one of the most likely used information for divergence and separation measurement and disparity of two density functions [27]. One advantage of KL(q|p) is its effectiveness in solving distributionally robust optimization problems [28]. Despite its computational and theoretical benefits, KL(q|p) faces certain challenges. These include asymmetries, which complicate optimal model selection [27], and the analytical complexity of KL(q|p) when comparing normal mixture models, along with a lack of practical computational algorithms [29]. Let p(x) and q(x) be two density functions of a continuous random variable *x* for support set $S_{X}$. KL(q|p) function is introduced as:

(4) $$KL\left(q|p\right)=\int_{x\in S_{X}}q\left(x\right)\ln\;\frac{q(x)}{p(x)}\underset{\cdot }{x}.$$

The basic structure of VBA is to minimize KL(q|p) and find an estimation for *p* with the density factorial over all variables, consisting of state, hidden, and all unknown parameters. For instance, *x* and *y* represent the state and hidden variables, respectively. The unknown parameters refer to the parameters of the prior distributions of *x* and *y*.

For simplicity, we assume a bivariate case of distribution p(x,y), and want to assess it via the alternative optimization, then we have:

(5) $$KL\left(q|p\right)=-H\left(q_{1}\right)-H\left(q_{2}\right)-{\langle \ln\;p\left(x,y\right)\rangle }_{q_{1}q_{2}},$$

where:

$$H\left(q_{1}\right)=-\int_{x\in S_{X}}q_{1}\left(x\right)\ln\;q_{1}\left(x\right)\underset{\cdot }{x}\;\;\mathrm{and}\;\;H\left(q_{2}\right)=-\int_{y\in S_{Y}}q_{2}\left(y\right)\ln\;q_{2}\left(y\right)\underset{\cdot }{y},$$

are, respectively, the Shannon entropies of *x* and of *y*, and:

$${\langle \ln p\left(x,y\right)\rangle }_{q_{1}q_{2}}=\int \int_{\left(x,y\right)\in S_{XY}}q_{1}\left(x\right)q_{2}\left(y\right)\ln\;p\left(x,y\right)\underset{\cdot }{x}\underset{\cdot }{y}.$$

$H(q_{1})$ and $H(q_{2})$ are fixed term, so the minimization is only on ${\langle \ln\;p\left(x,y\right)\rangle }_{q_{1}q_{2}}$. Now, differentiating the Equation (5) with respect to $q_{1}$, and then with respect to $q_{2}$ and equating them to zero, we obtain:

(6) $$q_{1}\left(x\right)\propto \exp\left\{{\langle \ln\;p\left(x,y\right)\rangle }_{q_{2}\left(y\right)}\right\}\;\mathrm{and}\;q_{2}\left(y\right)\propto \exp\left\{{\langle \ln\;p\left(x,y\right)\rangle }_{q_{1}\left(x\right)}\right\}.$$

These results can be easily extended to more dimensions [10]. They do not have any closed form, because they depend on the expression of p(x,y) and those of $q_{1}$ and $q_{2}$. An interesting case is the case of exponential families and conjugate priors, where writing:

(7) $$p\left(x,y\right)=p\left(x|y\right)p\left(y\right),\;\mathrm{and}p\left(y|x\right)=\frac{p(x,y)}{p(x)}=\frac{p(x|y)p(y)}{p(x)},$$

we can consider p(y) as prior, p(x|y) as the likelihood, and p(y|x) as the posterior distributions. We know that, if p(x,y) is in the exponential families, $q_{1}\left(x\right)$ and $q_{2}\left(y\right)$ are also in the exponential families, and $q_{1}\left(x\right)$ is conjugate to p(y|x) and $q_{2}\left(y\right)$ is conjugate to p(x|y). To illustrate all these properties, we give details of these expressions for a first simple example of Normal-Inverse-Gamma p(x,y)=N(x|μ,y)IG(y|α,β) with $q_{1}\left(x\right)=N\left(x|\mu^{\prime },v\right)$ and $q_{2}=IG\left(y|\alpha ,\beta \right)$. For this simple case, first we give the expression of KL(q|p) with $q_{1}\left(x\right)=N\left(x|{\tilde{\mu }}^{\prime },\tilde{v}\right)$ and $q_{2}\left(y\right)=IG\left(y|\tilde{\alpha },\tilde{\beta }\right)$ as a function of the parameters $\theta =({\tilde{\mu }}^{\prime },\tilde{v},\tilde{\alpha },\tilde{\beta })$ and then the expressions of the four above-mentioned algorithms and we study their convergence.

For a numerical comparison, we start by generating n=100 samples from p(x,y)=N(x|0,y)IG(y|3,1), so we know the true parameters (μ=0,α=3,β=1). Then, for different initializations of $\theta =({\tilde{\mu }}^{\prime },\tilde{v},\tilde{\alpha },\tilde{\beta })$, we run the four algorithms and compare the results. The final resulting margin, which is in this case the Student-t $S(x|\mu ,\alpha ,\beta )=\int N(x|\mu ,y)IG(y|\alpha ,\beta )\underset{\cdot }{y}$. We can then compare the true S(x|μ,α,β) with the obtained $S(x|\hat{\mu },\hat{\alpha },\hat{\beta })$ as well as with $q\left(x,y|\hat{\mu },\hat{v},\hat{\alpha },\hat{\beta }\right)=N\left(x|\hat{\mu },\hat{v}\right)IG\left(y|\hat{\alpha },\hat{\beta }\right)$. As we see in the proceeding of the paper, the initialization is very important. When we have samples of (x,y), we can try to initialize the parameters by their empirical values obtained by simple classical methods such as the method of moments. Another issue is the stopping criteria, which can be based either on the KLD criterion during successive iterations or any distances between the parameters at successive iterations.

## 3. Normal-Inverse-Gamma Distribution Example

The purpose of this section is to explain in detail the process of performing calculations in the alternative analytic algorithm. For this, we consider a simple case Normal-Inverse-Gamma Distribution for which, we have all the necessary expressions. This distribution has many applications in fields, like finance, econometrics, engineering, and Machine Learning. The objective here is to compare the four different algorithms mentioned earlier. The practical problem considered here is the following:

Suppose the data as $z=z_{1},...z_{N}$ and model it by Z=X+E, where E∼N(0,v) and so Z|X∼N(X,v). Thus, we have X∼N(μ,Y) and Y∼IG(α,β), then:

$$p\left(x,y|z\right)=\frac{p(z|x,y)p(x,y)}{p(z)}=\frac{p(z|x)p(x|y)p(y)}{p(z)}.$$

Putting all the elements, we see that p(x,y|z) is a NIG model and not separable in *x* and *y*. We like to approximate it with a separable one $q\left(x,y\right)=q_{1}\left(x\right)q_{2}\left(y\right)$. However, in this simple case, assume we have a sensor, which delivers a physical quantity *X*, N times, $x=\{x_{1},x_{2},\cdots ,x_{N}\}$. We want to model these data. In the first step, we model it as $N(x|\mu^{\prime },v)$ with fixed $\mu^{\prime }$ and *v*. Then, it is easy to estimate the parameters $\left(\mu^{\prime },v\right)$ either by Maximum Likelihood or Bayesian strategy. If we assume that the model is Normal with unknown variance and call this variance *y* and assign an IG prior to it, then we have a model NIG for p(x,y). The NIG priors have been applied to the wavelet context with correlated structures because they are conjugated with Normal priors [30]. We choose Normal-Inverse-Gamma distribution because of this conjugated property and ease of handling.

We showed that the margins are St and IG. Working directly with St is difficult. So, we want to approximate it with a Normal $q_{1}\left(x\right)$. This is equivalent to approximating p(x,y) with $q_{1}\left(x\right)q_{2}\left(y\right)$. Now, we want to find the parameters $\mu^{\prime }$, *v*, α, and β, which minimize $KL(q_{1}q_{2}|p)$. This process is called MFVBA. Then, we compare four algorithms to obtain the parameters, which minimize $KL(q_{1}q_{2}|p)$. $KL(q_{1}q_{2}|p)$ is convex with respect to $q_{1}$ if $q_{2}$ is fixed and is convex with respect to $q_{2}$ if $q_{1}$ is fixed. So, we hope that the iterative algorithm converges. However, $KL(q_{1}q_{2}|p)$ may not be convex in the space of parameters. So, we have to study the shape of this criterion concerning the parameters ${\tilde{\mu }}^{\prime }$, $\tilde{v}$, $\tilde{\alpha }$, and $\tilde{\beta }$.

We want to find p(x). For this process, we assume a simple Normal model, but with unknown variance *y*. So that, the forward model can be written as p(x,y)=N(x|μ,y)IG(y|α,β). In this simple example, we know that p(x) is a Student-t distribution obtained by:

(8) $$S(x|\mu ,\alpha ,\beta )=\int N(x|\mu ,y)IG(y|\alpha ,\beta )\underset{\cdot }{y}.$$

We approximate three parameters θ=(μ,α,β) from the data x and find an approximated distribution q(x) for p(x).

The main idea is to find such $q_{1}\left(x\right)q_{2}\left(y\right)$ as an approximation of p(x,y). Here, we show the standard alternative analytic optimization, step by step. For this, we start by choosing the conjugate families $q_{1}\left(x\right)=N\left(x|{\tilde{\mu }}^{\prime },\tilde{v}\right)$ and $q_{2}\left(y\right)=IG\left(y|\tilde{\alpha },\tilde{\beta }\right)$. Note that ${\langle x\rangle }_{p}=\tilde{\mu }$ and ${\langle x\rangle }_{q_{1}}={\tilde{\mu }}^{\prime }$.

In the first step, we have to calculate lnp(x,y) mentioned earlier:

(9) $$\ln p\left(x,y\right)=c-\frac{1}{2}\ln y-\frac{1}{2y}{\left(x-\tilde{\mu }\right)}^{2}-\left(\tilde{\alpha }+\frac{1}{2}\right)\ln y-\frac{\tilde{\beta }}{y},$$

where *c* is a constant value term independent of *x* and *y*. First of all, to use the iterative algorithm given in (6), starting by $q_{1}=N\left(x|{\tilde{\mu }}^{\prime },\tilde{v}\right)$ we have to find $q_{2}\left(y\right)$, so we start by finding $q_{2}\left(y\right)$. The integration of lnp(x,y) is with respect to $q_{1}\left(x\right)$:

(10) $${\langle \ln\;p\left(x,y\right)\rangle }_{q_{1}}=c-\frac{1}{2y}{\langle {\left(x-\tilde{\mu }\right)}^{2}\rangle }_{q_{1}}-\left(\tilde{\alpha }+1\right)\ln\;y-\frac{\tilde{\beta }}{y}.$$

Since ${\langle {\left(x-\tilde{\mu }\right)}^{2}\rangle }_{q_{1}}=\tilde{v}+{\left(\tilde{\mu }-{\tilde{\mu }}^{\prime }\right)}^{2}$:

(11) $$q_{2}\left(y\right)\propto \exp\left[-\left(\tilde{\alpha }+1\right)\ln\;y-\left(\frac{\tilde{v}+{\left(\tilde{\mu }-{\tilde{\mu }}^{\prime }\right)}^{2}}{2}+\tilde{\beta }\right)\frac{1}{y}\right].$$

Thus, the function $q_{2}\left(y\right)$ is equivalent to an inverse gamma distribution $IG(\tilde{\alpha },\frac{\tilde{v}+{\left(\tilde{\mu }-{\tilde{\mu }}^{\prime }\right)}^{2}}{2}+\tilde{\beta })$. To do so, we only use $q_{1}$ distribution. We have to take integral of lnp(x,y) over $q_{2}$ to find $q_{1}$:

(12) $${\langle \ln\;p\left(x,y\right)\rangle }_{q_{2}}=c-\left(\tilde{\alpha }+1\right){\langle \ln\;y\rangle }_{q_{2}}-\left(\tilde{\beta }+\frac{1}{2}{\left(x-\tilde{\mu }\right)}^{2}\right){\langle \frac{1}{y}\rangle }_{q_{2}}.$$

Note that the first term does not depend on *x* and ${\langle \frac{1}{y}\rangle }_{q_{2}}=\frac{2\tilde{\alpha }}{2\tilde{\beta }+\tilde{v}+{\left(\tilde{\mu }-{\tilde{\mu }}^{\prime }\right)}^{2}}$, so:

(13) $$q_{1}\left(x\right)\propto \exp\left[-\frac{2\tilde{\alpha }}{2\tilde{\beta }+\tilde{v}+{\left(\tilde{\mu }-{\tilde{\mu }}^{\prime }\right)}^{2}}\left(\tilde{\beta }+\frac{1}{2}{\left(x-\tilde{\mu }\right)}^{2}\right)\right]\propto \exp\left[-\frac{{\left(x-\tilde{\mu }\right)}^{2}}{2\frac{2\tilde{\beta }+\tilde{v}+{\left(\tilde{\mu }-{\tilde{\mu }}^{\prime }\right)}^{2}}{2\tilde{\alpha }}}\right].$$

We see that $q_{1}$ is again a Normal distribution but with updated parameters $N(\tilde{\mu },\frac{2\tilde{\beta }+\tilde{v}+{\left(\tilde{\mu }-{\tilde{\mu }}^{\prime }\right)}^{2}}{2\tilde{\alpha }})$, so $\tilde{v}=\frac{2\tilde{\beta }+\tilde{v}+{\left(\tilde{\mu }-{\tilde{\mu }}^{\prime }\right)}^{2}}{2\tilde{\alpha }}$. Note that, we obtained the conjugal property: If p(x|y)=N(x|μ,y) and p(y)=IG(y|α,β), then $p\left(y|x\right)=IG\left(y|\alpha^{\prime },\beta^{\prime }\right)$ where $\mu^{\prime }$, $\alpha^{\prime }$ and $\beta^{\prime }$ are $\mu^{\prime }=\mu$, $\alpha^{\prime }=\alpha$, $\beta^{\prime }=\beta +\frac{2\beta +v+{\left(\mu -\mu^{\prime }\right)}^{2}}{2\alpha }$. In this case, we also know that $p\left(x|\alpha ,\beta \right)=St\left(x|\mu^{\prime },\alpha ,\beta \right)$.

In these calculations, we first calculated the distribution of $q_{2}$ and then $q_{1}$. If we do the opposite and first obtain $q_{1}$ and then $q_{2}$, the parameter *v* is eliminated in the iterative calculations and there is no recursive relationship between β and *v*, and it is only necessary to calculate the value of parameter β.

In standard alternate optimization, there is no need for an iterative process for $\tilde{\mu }$ and $\tilde{\alpha }$, which are approximated by $\tilde{\mu }=\mu_{0}$ and $\tilde{\alpha }=\alpha_{0}$, respectively. The situation for $\tilde{\beta }$ and $\tilde{v}$ is different because there are circular dependencies among them. So, the approximation needs an iterative process, starting from ${\tilde{\mu }}^{\left(1\right)}=\mu_{0}$, ${\tilde{v}}^{\left(1\right)}=v_{0}$, $\alpha^{\left(1\right)}=\alpha_{0}$, and $\beta^{\left(1\right)}=\beta_{0}$.

1. Standard alternate optimization algorithm:

$$\begin{array}{ccc} {\tilde{\alpha }}^{\left(k+1\right)} & = & {\tilde{\alpha }}^{\left(k\right)},\\ {\tilde{\beta }}^{\left(k+1\right)} & = & {\tilde{\beta }}^{\left(k\right)}+\frac{{\tilde{v}}^{\left(k\right)}+{\left(\tilde{\mu }-{\tilde{\mu }}^{\prime \left(k\right)}\right)}^{2}}{2},\\ {\tilde{\mu }}^{\prime \left(k+1\right)} & = & {\tilde{\mu }}^{\prime \left(k\right)},\\ {\tilde{v}}^{\left(k+1\right)} & = & \frac{2{\tilde{\beta }}^{\left(k+1\right)}+{\tilde{v}}^{\left(k\right)}+{\left(\tilde{\mu }-{\tilde{\mu }}^{\prime \left(k+1\right)}\right)}^{2}}{2{\tilde{\alpha }}^{\left(k+1\right)}}. \end{array}$$

This algorithm converges to $\tilde{v}=\left(2\tilde{\beta }+\tilde{v}+{\left(\tilde{\mu }-{\tilde{\mu }}^{\prime }\right)}^{2}\right)/\left(2\tilde{\alpha }\right)$, which gives $\tilde{v}=\left(2\tilde{\beta }+{\left(\tilde{\mu }-{\tilde{\mu }}^{\prime }\right)}^{2}\right)/\left(2\tilde{\alpha }-1\right)$ and $\tilde{\beta }=0$, so $\tilde{v}=0$ that means very strange convergence. That is why this alternate algorithm can not work in this case.

For other algorithms based on normal parameters, such as gradient-based and natural gradient algorithms, it is necessary to find the expression of $KL(q_{1}q_{2}|p)$ as a function of the normal parameters $\tilde{\theta }=\left(\tilde{\alpha },\tilde{\beta },{\tilde{\mu }}^{\prime },\tilde{v}\right)$:

(14) $$\begin{array}{cc} KL(\tilde{\theta })\propto & -\frac{1}{2}\ln\tilde{v}+\frac{1}{2}\left(\ln\;\tilde{\beta }-\psi_{0}\left(\tilde{\alpha }\right)\right)+\frac{\tilde{\alpha }\left(\tilde{v}+{\left(\tilde{\mu }-{\tilde{\mu }}^{\prime }\right)}^{2}\right)}{2\tilde{\beta }}. \end{array}$$

Then, we also need the gradient expression of $\nabla KL(\tilde{\theta })$ for $\tilde{\theta }$:

(15) $$\begin{array}{cc} \nabla KL\left(\tilde{\theta }\right)= & (\frac{\tilde{v}+{\left(\tilde{\mu }-{\tilde{\mu }}^{\prime }\right)}^{2}-\tilde{\beta }\psi_{1}\left(\tilde{\alpha }\right)}{2\tilde{\beta }},\;\frac{-\tilde{\alpha }\left(\tilde{v}+{\left(\tilde{\mu }-{\tilde{\mu }}^{\prime }\right)}^{2}\right)+\tilde{\beta }}{2{\tilde{\beta }}^{2}},\\ \; & \;\frac{\tilde{\alpha }\left({\tilde{\mu }}^{\prime }-\tilde{\mu }\right)}{\tilde{\beta }},\left.\;\frac{\tilde{\alpha }}{2\tilde{\beta }}-\frac{1}{2\tilde{v}}\right). \end{array}$$

The details are available in Appendix A.

2. The gradient-based algorithm with normal parameters:

$$\begin{array}{ccc} {\tilde{\alpha }}^{\left(k+1\right)} & = & {\tilde{\alpha }}^{\left(k\right)}-\gamma \left(\frac{{\tilde{v}}^{\left(k\right)}+{\left(\tilde{\mu }-{\tilde{\mu }}^{\prime \left(k\right)}\right)}^{2}-{\tilde{\beta }}^{\left(k\right)}\psi_{1}\left({\tilde{\alpha }}^{\left(k\right)}\right)}{2{\tilde{\beta }}^{\left(k\right)}}\right),\\ {\tilde{\beta }}^{\left(k+1\right)} & = & {\tilde{\beta }}^{\left(k\right)}-\gamma \left(\frac{-{\tilde{\alpha }}^{\left(k+1\right)}\left({\tilde{v}}^{\left(k\right)}+{\left(\tilde{\mu }-{\tilde{\mu }}^{\prime \left(k\right)}\right)}^{2}\right)+{\tilde{\beta }}^{\left(k\right)}}{2{\left[{\tilde{\beta }}^{\left(k\right)}\right]}^{2}}\right),\\ {\tilde{\mu }}^{\prime \left(k+1\right)} & = & {\tilde{\mu }}^{\prime \left(k\right)}-\gamma \left(\frac{{\tilde{\alpha }}^{\left(k+1\right)}\left({\tilde{\mu }}^{\prime \left(k\right)}-\tilde{\mu }\right)}{{\tilde{\beta }}^{\left(k+1\right)}}\right),\\ {\tilde{v}}^{\left(k+1\right)} & = & {\tilde{v}}^{\left(k+1\right)}-\gamma \left(\frac{{\tilde{\alpha }}^{\left(k+1\right)}}{2{\tilde{\beta }}^{\left(k+1\right)}}-\frac{1}{2{\tilde{v}}^{\left(k\right)}}\right), \end{array}$$

where γ is a fixed value for the gradient algorithm. We propose two values for γ be equal to 1 and $\frac{1}{∥\nabla KL(\tilde{\theta })∥}$. We need to calculate the Fisher information for the third algorithm based on the normal parameters. Then, the natural gradient based on $KL(\tilde{\theta })$ is calculated as follows:

$$\begin{array}{cc} \tilde{\nabla }KL\left(\tilde{\theta }\right)= & \left(-\frac{1}{2},\;-\frac{1}{2}\left(\tilde{v}+{\left(\tilde{\mu }-{\tilde{\mu }}^{\prime }\right)}^{2}\right),\;-\frac{\tilde{\alpha }\tilde{v}\left(\tilde{\mu }-{\tilde{\mu }}^{\prime }\right)}{\tilde{\beta }},\;-\frac{\tilde{v}\left(\tilde{\beta }-\tilde{\alpha }\tilde{v}\right)}{\tilde{\beta }}\right). \end{array}$$

The corresponding algorithm is in the following:

3. The natural gradient algorithm:

$$\begin{array}{ccc} {\tilde{\alpha }}^{\left(k+1\right)} & = & {\tilde{\alpha }}^{\left(k\right)},\\ {\tilde{\beta }}^{\left(k+1\right)} & = & {\tilde{\beta }}^{\left(k\right)}+\frac{1}{2}\left({\tilde{v}}^{\left(k\right)}+{\left(\tilde{\mu }-{\tilde{\mu }}^{\prime \left(k\right)}\right)}^{2}\right),\\ {\tilde{\mu }}^{\prime \left(k+1\right)} & = & {\tilde{\mu }}^{\prime \left(k\right)}+\frac{{\tilde{\alpha }}^{\left(k+1\right)}{\tilde{v}}^{\left(k\right)}\left(\tilde{\mu }-{\tilde{\mu }}^{\prime \left(k\right)}\right)}{{\tilde{\beta }}^{\left(k+1\right)}},\\ {\tilde{v}}^{\left(k+1\right)} & = & {\tilde{v}}^{\left(k\right)}+\frac{{\tilde{v}}^{\left(k\right)}\left({\tilde{\beta }}^{\left(k+1\right)}-{\tilde{\alpha }}^{\left(k+1\right)}{\tilde{v}}^{\left(k\right)}\right)}{{\tilde{\beta }}^{\left(k+1\right)}}. \end{array}$$

We consider another sub-algorithm for gradient-based optimization with natural parameters, explained in detail in Appendix A. The algorithm for the last two components produces different results.

**Figure 1:**
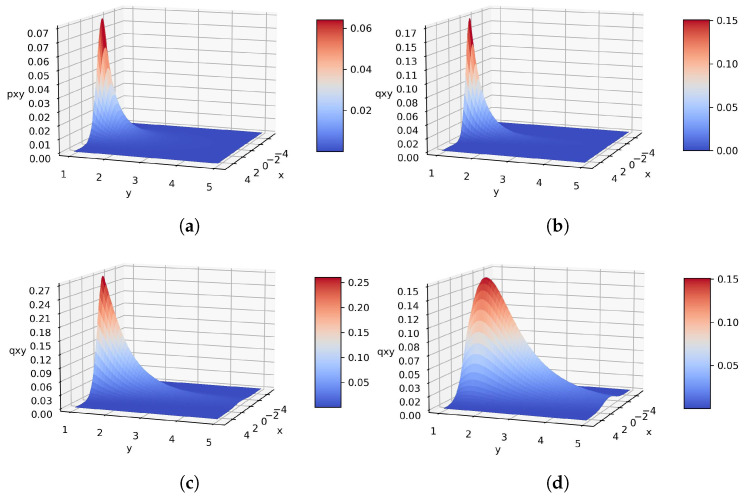

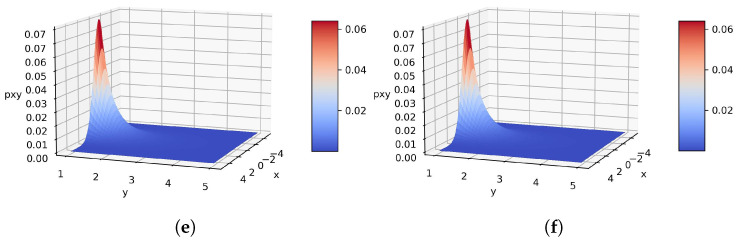
(**a**) Surface plot of the true model N(x|0,y)IG(y|3,1); (**b**) Surface plot of the alternative method with KL(·)=0.58; (**c**) Surface plot of the gradient method with γ=1 and KL(·)=0.58; (**d**) Surface plot of the gradient method with $\gamma ={∥KL\left(\cdot \right)∥}^{-1}$ and KL(·)=0.56; (**e**) Surface plot of the gradient method with natural parameters and KL(·)=0.59; (**f**) Surface plot of the natural gradient method with KL(·)=0.56.

**Figure 2:**
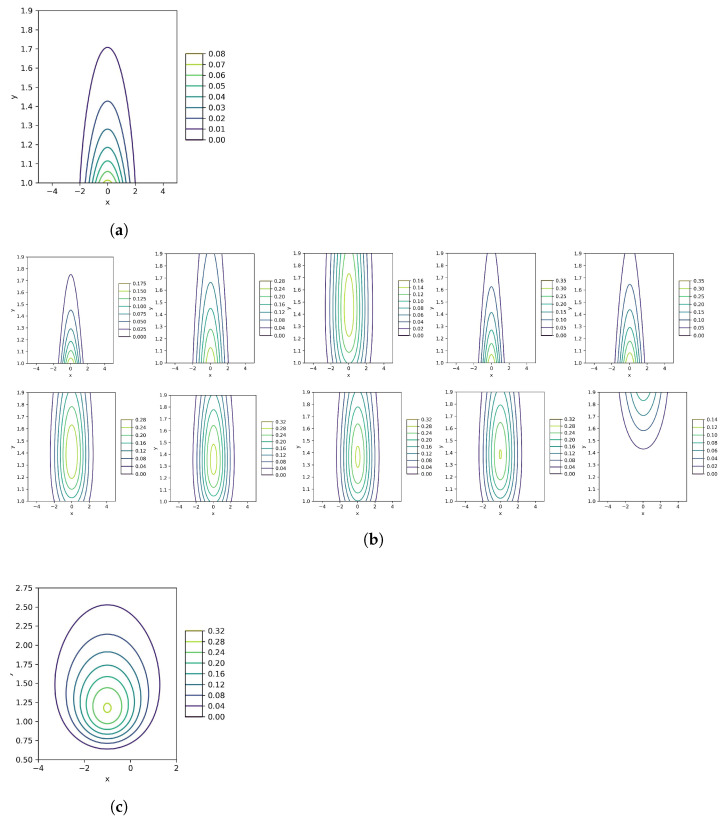

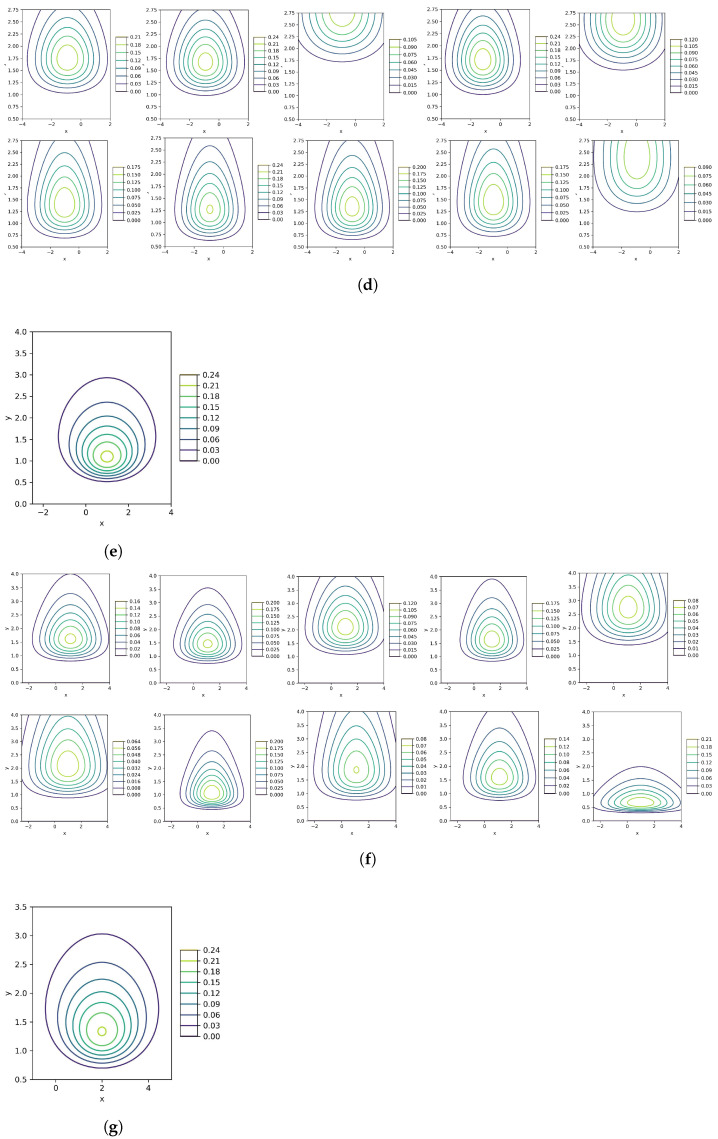

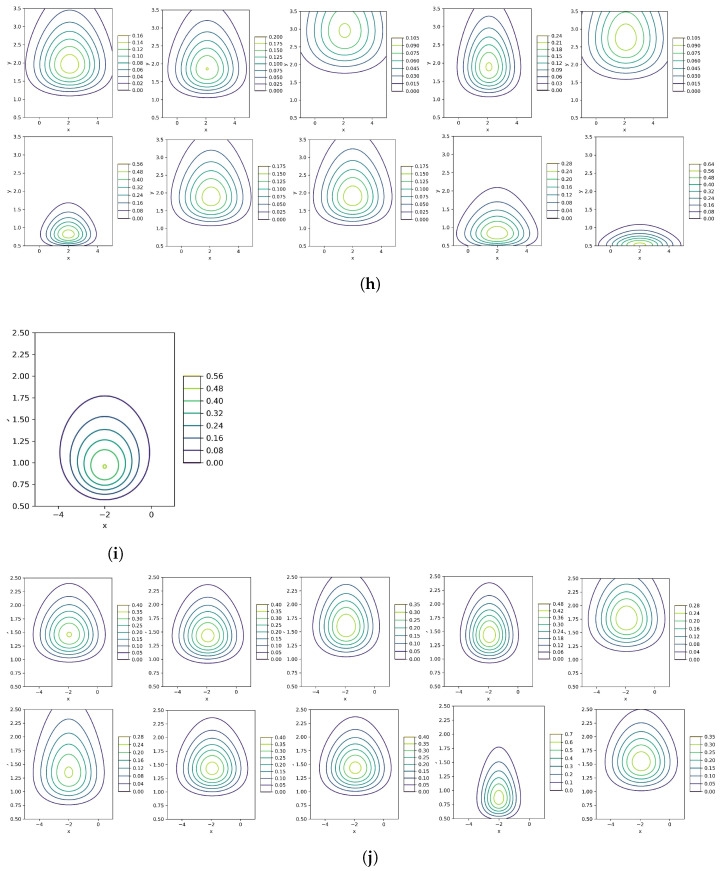
These plots are related to Table 1. The columns from left to right are related to the standard alternate optimization, parametric gradient-based with γ=1,∥KL(·)∥, gradient with natural parameters, and natural gradient, respectively. The rows of the estimated plots from top to bottom are based on MLE estimators and the initializations in the absence of information, respectively. The visualization inference is that in models N(x|0,y)IG(y|3,1) and N(x|−2,y)IG(y|10,11), the standard algorithm has the appropriate approximation, gradient-based method with $\gamma ={∥KL\left(\cdot \right)∥}^{-1}$ acts acceptable in models N(x|1,y)IG(y|4,6) and N(x|2,y)IG(y|6,10), and all methods have admissible estimations for N(x|−1,y)IG(y|7,10). (**a**) The true model N(x|0,y)IG(y|3,1); (**b**) The estimation plots of (**a**); (**c**) The true model N(x|1,y)IG(y|4,6); (**d**) The estimation plots of (**e**); (**e**) The true model N(x|−1,y)IG(y|7,10); (**f**) The estimation plots of (**c**); (**g**) The true model N(x|2,y)IG(y|6,10); (**h**) The estimation plots of (**g**); (**i**) The true model N(x|−2,y)IG(y|10,11); (**j**) The estimation plots of (**i**).

We generate n=100 samples from the model p(x,y)=N(x|1,y)IG(y|3,1) for the numerical computations. Thus, we know the exact values of the unknown parameters, just keeping in mind, not used in algorithms. The estimated parameters are in Table 1 using the alternative, gradient-based with $\gamma =1,\frac{1}{∥\nabla KL(\tilde{\theta })∥}$, natural gradient algorithms and gradient considering natural parameters along with their contour and surface plots in Figure 1 and Figure 2, respectively.

All four algorithms attempt to minimize the same criterion. So, the objectives are consistent, but the number of steps in the recursive process may vary. The requirements must meet the minimum KL(·). In this simple example of the Normal-Inverse-Gamma distribution with the model p(x,y)=N(x|1,y)IG(y|3,1), the convergence step numbers of the alternative, gradient-based with γ=1, ∥KL(·)∥, gradient with natural parameters, and natural gradient algorithms are 1, 10, 8, 2, and 9 using Maximum Likelihood Estimation (MLE) initializations. One crucial point is that the process should not be repeated excessively because we need to find the local minimum of KL(·). In this model, the VBA and gradient with respect to the natural parameters are the fastest, and the VBA provides the best approximation for the model depicted in Figure 2.

We simulate more models in Table 1 with the same joint distribution of the Normal-Inverse-Gamma along with different parameters. We draw all final results in Figure 2 and see their visual appearances. We start the recursive processes with two primary value groups for the unknown variables containing the MLE and the desired with no evidence. The selection for μ and *v* is quite simple because we have its data and can plot the histogram and approximately guess the correct values. The situation for α and β is different and a bit problematic to surmise perfectly. If we assume any outliers for α and β, the algorithm finds a minimization for KL(·) in the first stage of the iteration or another local minimum far from the truth. The more the iteration loop is executed, the KLD value increases or decreases in any case. Thus, the initializations are crucial to obtain the closest results for the unknown parameters. For instance, in N(x|1,y)IG(y|4,6) with elementary points μ=1, v=3.5, α=3, and β=3 in the natural gradient analytic algorithm, the repetitive result is the same as the initial points. So, these initializations are not suitable for the available data.

An algorithm with the lowest KLD value provides a more accurate estimate of the model parameters. As we discussed earlier, for the model N(x|0,y)IG(y|3,1), the first, fourth, and fifth algorithms estimate the parameters well, as shown in Figure 2, and Table 1. Remember, we use two values for γ in the gradient-based algorithm with normal parameters, so the total estimated values are five for each unknown parameter. The first three algorithms approximate the parameters of the model N(x|1,y)IG(y|4,6) well, but the issue lies in fitting the center, resulting in a shifted approximation. All methods perform well in the models N(x|−1,y)IG(y|7,10), N(x|2,y)IG(y|6,10), and N(x|−2,y)IG(y|10,11). The optimal options for N(x|−1,y)IG(y|7,10) include VBA, gradient with γ=1, and gradient with respect to natural parameters. Considering the model N(x|2,y)IG(y|6,10), the best approach is to use gradient algorithms. The best option for the final model is the alternative approximation.

**Figure 3:**
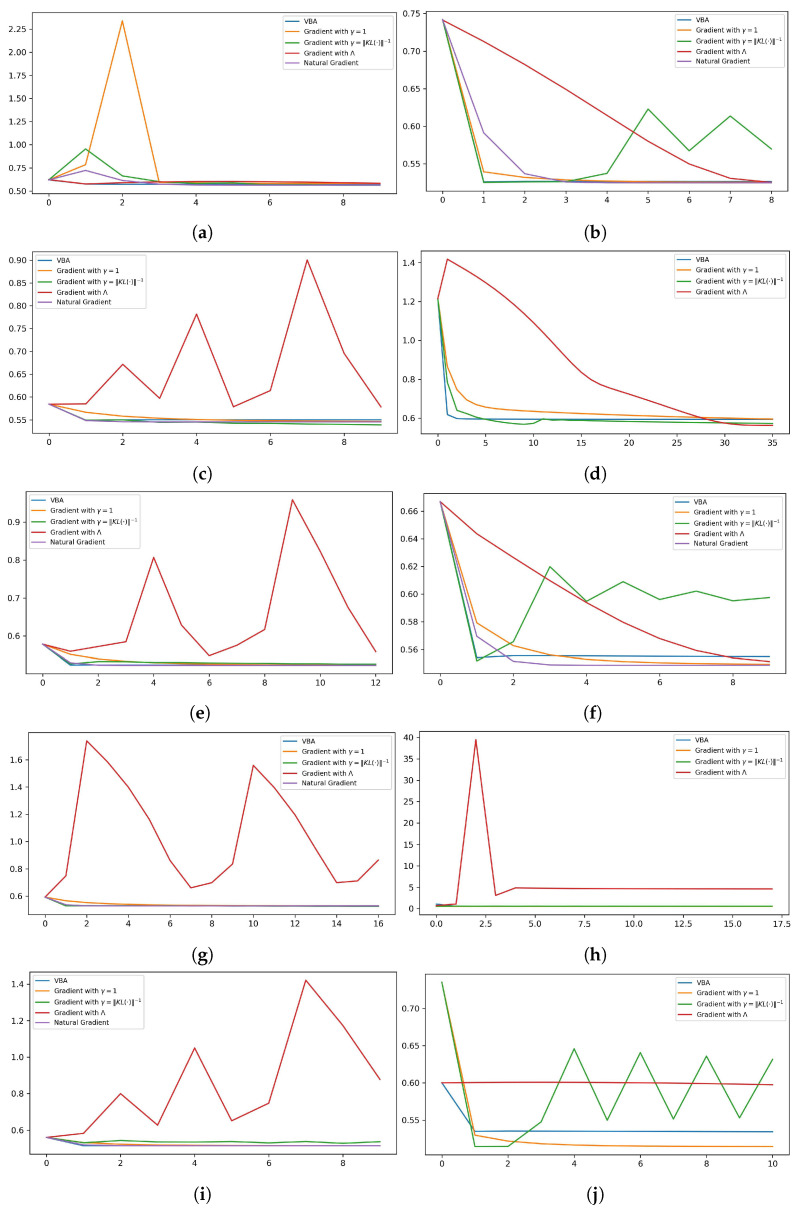
The vertical and horizontal axis are the KL(·) values and the number of iterations, respectively. The left and right columns are for the MLE and evidence-free initializations, respectively. (**a**,**b**) N(x|0,y)IG(y|3,1); (**c**,**d**) N(x|1,y)IG(y|4,6); (**e**,**f**) N(x|−1,y)IG(y|7,10); (**g**,**h**) N(x|2,y)IG(y|6,10). (**i**,**j**) N(x|−2,y)IG(y|10,11).

To gain an overview of the KL(·) process, we plot its trend with respect to the number of iterations in Figure 3. The left column shows the results for the MLE initializations, where the minimization of KL(·) occurs more quickly compared to the right column, which is associated with the evidence-free initializations. We do not plot the trend of the natural gradient’s KL(·) in the second and last two models. The reason is that the KL(·) becomes NaN in the initial iterations, so it is necessary to start the recursive process with some points estimated from the available data. The algorithms approximate the joint density function with a separable one but with different accuracy. In the following section, we tackle a more complex model.

## 4. Multivariate Normal-Inverse-Wishart Example

In the previous section, we explain how to perform VBA optimization methods to approximate a complicated joint distribution function by a tractable factorial of margins over a simple case study. In this section, a multivariate Normal case $p\left(x\right)=N\left(x|\tilde{\mu },\tilde{\Sigma }\right)$ is considered, which is approximated by $q\left(x\right)=\prod_{i}N\left(x_{i}|{\tilde{\mu }}_{i},{\tilde{\nu }}_{i}\right)$ for different shapes for the covariance matrix $\tilde{\Sigma }$.

We assume that the basic structure of an available data set is multivariate Normal with the unknown mean vector $\tilde{\mu }$ and variance-covariance matrix $\tilde{\Sigma }$. Their joint prior distribution is a Normal-Inverse-Wishart distribution of $NIW(\tilde{\mu },\tilde{\Sigma }|{\tilde{\mu }}_{0},\tilde{\kappa },\tilde{\Psi },\tilde{\nu })$ defined by $N\left(\tilde{\mu }|{\tilde{\mu }}_{0},\frac{1}{\tilde{\kappa }}\tilde{\Sigma }\right)IW\left(\tilde{\Sigma }|\tilde{\Psi },\tilde{\nu }\right)$, which is the generalized form of classical NIG. One of its applications is in image segmentation tasks. The posteriors are multivariate Normal for the mean vector and Inverse Wishart for the variance-covariance matrix. Inverse Wishart distribution has many properties on the density function of unknown variances, and so there is a variety of references. For example, Bouriga and Féron [31] worked on covariance matrix estimation using Inverse Wishart distribution. In this regard, they inquired about posterior properties and Bayesian risk. Also, they applied Daniels and Kass prior [32] to the hyper-parameters of Σ.

Before approximating the expression of $p(x,\tilde{\mu },\tilde{\Sigma })$, let us define some notations that we are using here:

$$x=\left[\begin{array}{c} x_{1}\\ \vdots \\ x_{p} \end{array}\right],\;\tilde{\mu }=\left[\begin{array}{c} {\tilde{\mu }}_{1}\\ \vdots \\ {\tilde{\mu }}_{p} \end{array}\right],\;\mathrm{and}\tilde{\Sigma }=\left[\begin{array}{ccc} {\tilde{\Sigma }}_{11} & \cdots & {\tilde{\Sigma }}_{1p}\\ {\tilde{\Sigma }}_{21} & \cdots & {\tilde{\Sigma }}_{2p}\\ \vdots & \ddots & \vdots \\ {\tilde{\Sigma }}_{p1} & \cdots & {\tilde{\Sigma }}_{pp} \end{array}\right].$$

Since the Normal-Inverse-Wishart distribution is a conjugate prior distribution for multivariate Normal, the posterior distribution of $\tilde{\mu }$ and $\tilde{\Sigma }$ again belongs to the same family, and their corresponding margins are:

(16) $$MN\left(\frac{\tilde{\kappa }{\tilde{\mu }}_{0}+n\bar{x}}{\tilde{\kappa }+n},\frac{1}{\tilde{\kappa }+n}\tilde{\Lambda }\right),\;IW\left(\tilde{\Lambda }+\tilde{\Psi }+\sum_{i=1}^{n}\left(x_{i}-\bar{x}\right){\left(x_{i}-\bar{x}\right)}^{⊺},\tilde{\nu }+n\right),$$

where *n* and x are the sample size and observations of x, respectively. The proof is shown in Appendix B.

For other algorithms, we define $\tilde{\Theta }=\left(\tilde{\kappa },{\tilde{\mu }}_{0},\tilde{\Lambda },\tilde{\nu },\tilde{\Psi }\right)$ for calculation of $KL(\tilde{\Theta })$. After some tedious computation available in Appendix C, the $KL(\tilde{\Theta })$ is equivalence by:

(17) $$\begin{array}{c} KL\left(\tilde{\Theta }\right)\propto \frac{p-1}{2}\ln\tilde{\kappa }+\frac{1}{2}\ln∥\tilde{\Psi }{\tilde{\Lambda }}^{-1}∥+\frac{\tilde{\nu }}{2}\mathrm{Tr}\left[{\tilde{\Psi }}^{-1}\tilde{\Lambda }+I\right]-\frac{p\tilde{\nu }}{2}-\frac{1}{2}\sum_{i=1}^{p}\psi_{0}\left(\frac{\tilde{\nu }-p+i}{2}\right), \end{array}$$

and the corresponding gradient-based and natural gradient algorithms are determined similar to Section 3 based on $\nabla KL(\tilde{\Theta })$, also available in Appendix C.

To present the performance of the four algorithms, we work on a data set coming from x∼NIW(x|μ,Σ) whose parameters have the below density structure:

(18) $$\mu ∼MN\left(\mu |\left[\begin{array}{c} 2\\ 1 \end{array}\right],\frac{1}{2}\left[\begin{array}{cc} 3 & -1\\ -1 & 1 \end{array}\right]\right),\;\Sigma ∼IW\left(\Sigma |\left[\begin{array}{cc} 3 & -1\\ -1 & 1 \end{array}\right],\;6\right).$$

**Figure 4:**
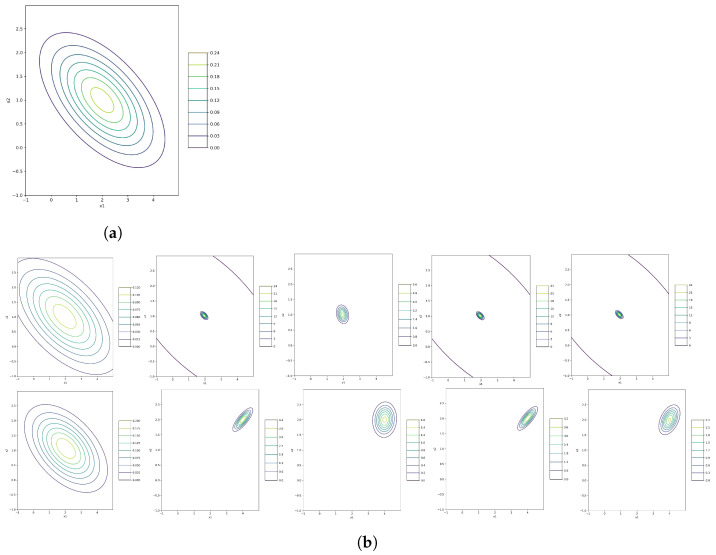
The corresponding estimation values are in Table 2. From the left to right, the columns show the contour plots with MLE and evidence-free initialization, respectively. The first raw is for the result of the standard alternative optimization, and the two others are for gradient and natural gradient algorithms, respectively. In this example, the standard alternative method has the most fitted result. (**a**) Model (18); (**b**) Estimations of the Model.

We use only the data of x in the estimation processes. The results of algorithms are in Table 2 and drowned in Figure 4 along with its true contour plot of the model. In this figure, we have two different rows of results. In the first row, we use the MLE estimation of the mean and covariance parameters as our pre-information to start the algorithms and find the best local minimum for the KL(·). In this regard, we put some gusted value for κ and ν. At the bottom of the figure, we use some uninformative initializations. This choice is crucial because the evidence-free primitive starter may end in some local optimization, which is far from the part we need. Thus, using MLE prevents this problem to a great extent. Although the result for the alternative optimization is good using evidence-free elementary points in Figure 4, it does not always work this way and depends on luck. Other gradient-based and natural gradient algorithms do not work well in this example. One reason can be the more dimensions, the worse the results.

In Table 2, we have more models with a variety of dimensions 2, 3, and 5 applying two initialization groups, MLE and uninformative starters. In all cases, the standard alternative algorithm has the closest estimation to the real model in the case of KL(·) minimization. The larger the KL(·), the greater the distance between the true and estimated posterior distributions. Therefore, all gradient-based and natural gradient algorithms are not worthy practices to extract the posterior distribution with more dimensions in the data, while our alternative acts better in this situation. In Table 3, we provide a Normal-Inverse-Wishart model with 10 dimensions. In comparison, the alternative method approximates the distribution with so many parameters more precisely than the other methods, which seems easy to use with any large-scale dimension dataset, and the result is suitable enough.

## 5. Simple Linear Inverse Problem

The third example is the case of linear inverse problems g=Hf+ϵ with priors $p\left(\epsilon \right)=N\left(\epsilon |0,v_{\epsilon }I\right)$ and $p\left(f\right)=N\left(f|0,\mathrm{diag}\left[v\right]\right)$, where $f=[f_{1},f_{2},\cdots ,f_{N}]$ and $v=[v_{1},v_{2},\cdots ,v_{N}]$ for which we have $p\left(f,v|g\right)\propto p\left(g|f,v_{\epsilon }\right)p\left(f|v\right)p\left(v\right)$ with $p\left(g|f,v_{\epsilon }\right)=N\left(g|Hf,v_{\epsilon }I\right)$, $p\left(f|v\right)=N\left(f|0,\mathrm{diag}\left[v\right]\right)$ and $p\left(v|\alpha ,\beta \right)=\prod_{j}IG\left(v_{j}|\alpha ,\beta \right)$, [33].

In the inverse problem, we have the same multivariate case, except in Section 3 that this time, the sensor does not give directly f, but g, such that g=Hf+ϵ where ϵ is again a Normal with known or unknown variance. In the first step, we assume that the variance $v_{\epsilon }$ and H are known. H is called the transfer function of the sensor. So, this time we have p(g,f,v)=N(g|f)N(f|v)IG(v) and we approximate it by $q_{1}\left(\tilde{f}|g,\tilde{v}\right)q_{2}\left(\tilde{v}\right)$. The main reason of this choice for $q_{1}\left(\tilde{f}|g,\tilde{v}\right)$ is that, when g and $\tilde{v}$ are known, this becomes a Normal $q_{1}\left(\tilde{f}|g,\tilde{v}\right)=N\left(\tilde{f}|\tilde{\mu },\tilde{\Sigma }\right)$ where the expressions of $\tilde{\mu }$ and $\tilde{\Sigma }$ are given in the paper. Now, we have a multivariate NIG problem. Thus, the joint distribution of g, f, and v is estimated by VBA as the following relation:

(19) p(f,v|g)∝p(f|g,v)p(v).

Although we can mathematically compute the margins in this particular example, we desire to approximate them via the iterative alternative algorithm $q\left(g,\tilde{f},\tilde{v}\right)=q_{1}\left(g|\tilde{f}\right)q_{2}\left(\tilde{f}\right)$$q_{3}\left(\tilde{v}\right)$ compared them with gradient and natural gradient-based algorithms. The objective function is the estimation of $q_{2}\left(\tilde{f}\right)$, but in the recursive process, $q_{1}\left(g|\tilde{f}\right)$ and $q_{3}\left(\tilde{v}\right)$ are updated, too. For simplicity, we suppose that the transposition matrix H is an identical matrix I. The final outputs are as follows, with the details and algorithm in Appendix D:

(20) $$\begin{array}{c} \tilde{f}∼MN\left(\frac{{\tilde{\mu }}_{\tilde{f}}}{1+\frac{2\tilde{v_{\epsilon }}\tilde{\alpha }}{n({\tilde{v}}_{\tilde{f}}+{\tilde{\mu }}_{\tilde{f}}^{2}+2\tilde{\beta })}},\mathrm{diag}\left[\frac{\tilde{v_{\epsilon }}\left({\tilde{v}}_{\tilde{f}}+{\tilde{\mu }}_{\tilde{f}}^{2}+2\tilde{\beta }\right)}{n\left({\tilde{v}}_{\tilde{f}}+{\tilde{\mu }}_{\tilde{f}}^{2}\right)+2n\tilde{\beta }+2\tilde{v_{\epsilon }}\tilde{\alpha }}\right]\right),\\ g∼N\left({\tilde{\mu }}_{\tilde{f}},\tilde{v_{\epsilon }}I\right),\;\mathrm{and}\;{\tilde{v}}_{k}∼IG\left({\tilde{\alpha }}_{k},\frac{{\tilde{v}}_{{\tilde{f}}_{k}}+{\tilde{\mu }}_{{\tilde{f}}_{k}}^{2}}{2}+{\tilde{\beta }}_{k}\right),\;k=1,\cdots ,p. \end{array}$$

The corresponding $KL(\tilde{\theta })$ for $\tilde{\theta }=\left({\tilde{\mu }}_{g},\tilde{v_{\epsilon }},{\tilde{v}}_{\tilde{f}},\tilde{\alpha },\tilde{\beta }\right)$ is below and the details are available in Appendix E along with its gradient and other algorithms:

$$\begin{array}{cc} KL(\tilde{\theta })\propto & -\frac{1}{2}\sum_{j=1}^{p}\ln\left({\tilde{v}}_{{\tilde{f}}_{j}}\right)+\frac{n}{2\tilde{v_{\epsilon }}}\sum_{j=1}^{p}\left({\tilde{\mu }}_{g_{j}}^{2}+{\tilde{v}}_{{\tilde{f}}_{j}}\right)+\sum_{j=1}^{p}\frac{{\tilde{\alpha }}_{j}{\tilde{v}}_{{\tilde{f}}_{j}}}{{\tilde{\beta }}_{j}}-\frac{1}{2}\sum_{j=1}^{p}\psi_{0}\left({\tilde{\alpha }}_{j}\right)+\frac{1}{2}\sum_{j=1}^{p}\ln{\tilde{\beta }}_{j}. \end{array}$$

We choose a model to see the performance of these margins and compare them with gradient-based considering normal and natural parameters and natural gradient algorithms. The selected model is g=Hf+ϵ with the following knowledge:

(21) $$\begin{array}{cc} H & =I,\\ f & ∼MN(f|0,\mathrm{diag}\left[v_{1},v_{2}\right]),\\ v_{1} & ∼IG(v_{1}|3,2)\\ v_{2} & ∼IG(v_{2}|4,3)\\ \epsilon & ∼MN(\epsilon |0,I). \end{array}$$

**Figure 5:**
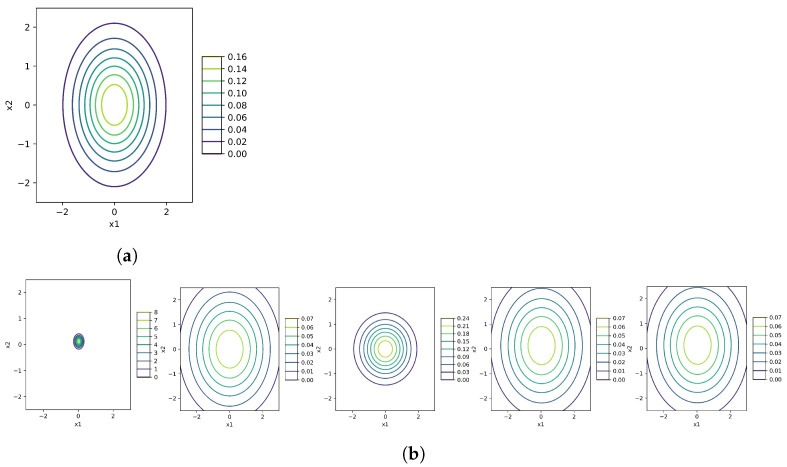
(**a**) True contour plot of model (5), almost separable; (**b**) These are the estimation of the model based on the standard alternative analytic approximation, gradient-based algorithm with $\gamma =1,∥KL\left(\tilde{\theta }\right){∥}^{-1}$ and concerning the natural parameters, and natural gradient algorithm, respectively, from left to right.

In the assessment procedure, we do not apply the above information. The outputs of algorithms are shown in Figure 5, as well as the actual contour plot. The KL(·) for the four algorithms, starting from 108.91, are 4.93, 108.91, 19.57, 108.91, and 108.91, respectively. In this example, the best diagnosis is from the alternative algorithm with the minimum of KL(·).

The objective of the inverse problem is to approximate the distribution of f. We use the distribution of g in Figure 5 to show the number of method accuracies. In Figure 5, we can observe improved approximations for the second and last two plots. In these algorithms, the best choice is the initialization, and the distribution has a higher KL(·) by reputation. Here, are the conjectures of the standard alternative, gradient-based with $\gamma =1,∥KL\left(\tilde{\theta }\right){∥}^{-1}$ and natural parameters, and natural gradient algorithms, respectively:

$$\left\{\begin{array}{c} \mu_{\tilde{f}}=\left[\begin{array}{c} 0.036\\ 0.118 \end{array}\right],\\ {\tilde{\Sigma }}_{\tilde{f}}=\left[\begin{array}{cc} 0.023 & 0\\ 0 & 0.022 \end{array}\right], \end{array}\right.\left\{\begin{array}{c} \mu_{\tilde{f}}=\left[\begin{array}{c} 0.038\\ 0.129 \end{array}\right],\\ {\tilde{\Sigma }}_{\tilde{f}}=\left[\begin{array}{cc} 2.459 & 0\\ 0 & 2.178 \end{array}\right], \end{array}\right.\left\{\begin{array}{c} \mu_{\tilde{f}}=\left[\begin{array}{c} 0\\ 0 \end{array}\right],\\ {\tilde{\Sigma }}_{\tilde{f}}=\left[\begin{array}{cc} 0.891 & 0\\ 0 & 0.523 \end{array}\right], \end{array}\right.$$

$$\left\{\begin{array}{c} \mu_{\tilde{f}}=\left[\begin{array}{c} 0.04\\ 0.13 \end{array}\right],\\ {\tilde{\Sigma }}_{\tilde{f}}=\left[\begin{array}{cc} 2.459 & 0\\ 0 & 2.178 \end{array}\right], \end{array}\right.\left\{\begin{array}{c} \mu_{\tilde{f}}=\left[\begin{array}{c} 0.04\\ 0.13 \end{array}\right],\\ {\tilde{\Sigma }}_{\tilde{f}}=\left[\begin{array}{cc} 2.459 & 0\\ 0 & 2.178 \end{array}\right]. \end{array}\right.$$

We simulate additional g=Hf+ϵ models with varying dimensions in Table 4. Based on the results, the optimal posterior is obtained from the standard alternative algorithm when we increase the dimensions, as it exhibits lower values of KL(·). In the inverse problem, the primary focus is on observing f, so another way to compare could be the accuracy of $v_{f}$. When it comes to the variance-covariance matrix of f, the most effective algorithm is parametric natural gradient optimization for approximating the posterior distribution of f because it yields results that closely align with the data of f. We would like to remind you that we do not use f data in the recursive processes; we only have g data. It is applicable to real-world situations, but since this is a simulated example, we have the f data, but we do not utilize it. In the example above, we observe that the alternative method yields a lower KL(·) in the two-dimensional dataset, too. As the number of dimensions increases, so does the value of KL(·). However, the accuracy of the variance-covariance of f is not reduced.

## 6. Conclusions

This paper presents four approximation methods for estimating the density functions of hidden variables, referred to as VBA. We also consider three examples of the Normal-Inverse-Gamma, Normal-Inverse-Wishart, and linear inverse problems. We provide the details of the first model here and include the details for two other examples in the appendices. In all three models, the parameters are unobserved and estimated using recursive algorithms. We attempt to approximate the joint complex distribution by simplifying the margin factorials to resemble independent cases. We compare the performance and accuracy of VBA algorithms. The standard alternative analytic optimization algorithm demonstrates the highest robustness in minimizing KLD, particularly as the number of dimensions increases, and converges fairly quickly. The numerical computation cost is negligible in our examples because we find the explicit form for each parameter, and the algorithms are well-formulated. The main difference in algorithms lies in the accuracy of the results. They estimate the complex joint distribution using separable ones. In the linear inverse problem, the standard alternative algorithm yields lower KL(·) values, while the parametric gradient algorithm with $\gamma ={∥KL\left(\cdot \right)∥}^{-1}$ produces variance-covariance matrices that are closest to the real ones. The overall performance of the alternative is most satisfactory, especially in high dimensions.

## Figures and Tables

**Table 1:** Simulation results for five different models using the Normal-Inverse-Gamma distribution are presented here. We use two initialization point groups. The first column shows the MLE for each parameter. The second column contains some intended initializations that we refer to as evidence-free initialization. They are not based on any information and are optional. It is evident that when we apply data-derived points, the results are highly accurate. However, when we rely on evidence-free points, the results deviate significantly from the true models. Also, we calculate the KL(·) values for the initializations and the final estimations for each method. Each algorithm optimizes the function KL(·) in different ways. The alternative provides acceptable parameter estimations for the correct parameters, although the criterion is to minimize KL(·). The corresponding plots can be found in Figure 2 to provide a comprehensive overview of the approximations and facilitate comparisons.

Models	Algorithm↓		MLE Initializations					Evidence-Free Initializations				
			Parameters				KL(·)	Parameters				KL(·)
			$\mu^{\prime }$	v	α	β		$\mu^{\prime }$	v	α	β	
N(x|0,y)IG(y|3,1)	Initial Points →		0.04	0.59	3.79	1.47	0.62	0	0.5	10	15	0.74
	Alternative		0.04	0.54	3.79	1.77	0.58	0	1.55	10	15.25	0.53
	Gradient	γ=1	0.04	1.05	3.27	3.39	0.58	0.04	1.45	10.09	14.95	0.53
		$\gamma =∥\nabla KL\left(\tilde{\theta }\right){∥}^{-1}$	0.04	1.77	4.29	7.64	0.56	0.04	1.50	10.05	15.07	0.53
		Natural Parameters	0	0.55	3.71	2.73	0.59	0	1.52	10.17	15.47	0.52
	Natural Gradient		0.04	0.74	4.29	3.54	0.56	0.04	2.52	10.50	27.67	0.52
N(x|1,y)IG(y|4,6)	Initial Points →		1.09	1.20	5.35	9.62	0.58	1	3.5	3	3	1.22
	Alternative		1.09	2.02	5.35	10.22	0.55	1	3.24	3	8.37	0.60
	Gradient	γ=1	1.09	1.68	5.56	9.55	0.55	1.09	1.40	3.26	4.45	0.58
		$\gamma =∥\nabla KL\left(\tilde{\theta }\right){∥}^{-1}$	1.09	2.33	6.15	14.96	0.54	1.09	2.55	2.53	6.58	0.68
		Natural Parameters	1.36	1.54	5.44	10.44	0.58	1.10	1.91	4.18	8.21	0.56
	Natural Gradient		1.09	2.94	5.85	18.66	0.55	1	3.5	3	3	1.22
N(x|−1,y)IG(y|7,10)	Initial Points →		−0.92	1.11	10.82	19.99	0.52	−1	0.75	5	8	0.67
	Alternative		−0.92	1.95	10.82	20.54	0.52	−1	1.75	5	8.38	0.55
	Gradient	γ=1	−0.92	1.77	10.94	19.94	0.52	−0.92	1.48	5.24	7.91	0.55
		$\gamma =∥\nabla KL\left(\tilde{\theta }\right){∥}^{-1}$	−0.92	3.24	11.84	36.34	0.52	−0.86	1.69	5.17	8.25	0.55
		Natural Parameters	−1.19	1.6	10.87	20.25	0.55	−0.97	1.68	5.20	9.01	0.55
	Natural Gradient		−0.92	2.72	11.32	32.21	0.52	−0.92	2.60	5.50	15.58	0.55
N(x|2,y)IG(y|6,10)	Initial Points →		2.09	1.21	8.86	18.66	0.59	2	2	8	5	1.06
	Alternative		2.09	2.24	8.86	19.27	0.53	2	0.98	8	7.34	0.54
	Gradient	γ=1	2.09	1.91	9.01	18.61	0.53	2.09	2.08	8.94	18.65	0.53
		$\gamma =∥\nabla KL\left(\tilde{\theta }\right){∥}^{-1}$	2.09	3.15	10.27	33.24	0.52	2	2	8.86	18.66	0.53
		Natural Parameters	2.09	1.21	8.86	18.66	0.59	2	2	5	5	0.71
	Natural Gradient		2.09	2.88	9.36	28.39	0.53	2	2	8.5	5	1.06
N(x|−2,y)IG(y|10,11)	Initial Points →		−1.93	0.96	17.52	26.54	0.56	−2	0.5	8	7	0.60
	Alternative		−1.93	1.57	17.52	27.02	0.51	−2	1.61	8	12.13	0.53
	Gradient	γ=1	−1.93	1.47	17.56	26.51	0.51	−1.93	1.48	17.57	26.51	0.51
		$\gamma =∥\nabla KL\left(\tilde{\theta }\right){∥}^{-1}$	−1.93	1.33	17.42	29.43	0.53	−1.93	1.50	17.55	26.57	0.51
		Natural Parameters	−1.93	0.96	17.52	26.54	0.56	−2.01	0.56	8.20	7.96	0.60
	Natural Gradient		−1.93	1.80	18.02	33.32	0.51	−1.93	1.58	18.02	29.30	0.51

**Table 2:** There are some models with their estimation via four optimization algorithms with two different initializations. The lower the KL(·), the more accurate the fitted posterior distribution.

Models	Algorithm↓		MLE Initializations						Evidence-Free Initializations					
			Parameters					KL(·)	Parameters					KL(·)
			$\mu_{0}$	κ	Λ	$\frac{\Psi }{\nu +n-p-1}$	ν		$\mu_{0}$	κ	Λ	$\frac{\Psi }{\nu +n-p-1}$	$\tilde{\nu }$	
	Initial Points →		$\left[\begin{array}{c} 1.94\\ 1.01 \end{array}\right]$	5	$\left[\begin{array}{cc} 158.08 & -50.41\\ -50.41 & 49.38 \end{array}\right]$	$\left[\begin{array}{cc} 0.01 & 0\\ 0 & 0 \end{array}\right]$	5	520.59	$\left[\begin{array}{c} 4\\ 2 \end{array}\right]$	5	$\left[\begin{array}{cc} 10 & -5\\ -5 & 4 \end{array}\right]$	$\left[\begin{array}{cc} 0.10 & 0.05\\ 0.05 & 0.04 \end{array}\right]$	5	21.91
	Alternative		$\left[\begin{array}{c} 1.94\\ 1.01 \end{array}\right]$	5	$\left[\begin{array}{cc} 1.51 & -0.48\\ -0.48 & 0.47 \end{array}\right]$	$\left[\begin{array}{cc} 3.03 & -0.96\\ -0.96 & 0.96 \end{array}\right]$	5	5.59	$\left[\begin{array}{c} 2.04\\ 1.06 \end{array}\right]$	5	$\left[\begin{array}{cc} 0.10 & -0.05\\ -0.05 & 0.04 \end{array}\right]$	$\left[\begin{array}{cc} 1.66 & -0.47\\ -0.47 & 0.53 \end{array}\right]$	5	7
		γ=1	$\left[\begin{array}{c} 1.94\\ 1.01 \end{array}\right]$	5	$\left[\begin{array}{cc} 158.08 & -50.41\\ -50.41 & 49.38 \end{array}\right]$	$\left[\begin{array}{cc} 0.01 & -0.01\\ -0.01 & 0.01 \end{array}\right]$	5	520.59	$\left[\begin{array}{c} 4\\ 2 \end{array}\right]$	5	$\left[\begin{array}{cc} 10 & -5\\ -5 & 4 \end{array}\right]$	$\left[\begin{array}{cc} 0.10 & 0.05\\ 0.05 & 0.04 \end{array}\right]$	5	21.91
$NIW\left(\tilde{\mu },\tilde{\Sigma }|\left[\begin{array}{c} 2\\ 1 \end{array}\right],2,\left[\begin{array}{cc} 3 & -1\\ -1 & 1 \end{array}\right],6\right)$	Gradient	$\gamma =∥\nabla KL\left(\tilde{\Theta }\right){∥}^{-1}$	$\left[\begin{array}{c} 1.94\\ 1.01 \end{array}\right]$	4.85	$\left[\begin{array}{cc} 158.04 & -50.42\\ -50.42 & 49.32 \end{array}\right]$	$\left[\begin{array}{cc} 0.04 & -0.01\\ -0.01 & 0.03 \end{array}\right]$	3.99	88.82	$\left[\begin{array}{c} 4\\ 2 \end{array}\right]$	1.31	$\left[\begin{array}{cc} 7.82 & -1.72\\ -1.72 & 2.79 \end{array}\right]$	$\left[\begin{array}{cc} 0.14 & 0.01\\ 0.01 & 0.09 \end{array}\right]$	3.99	2.64
		Natural Parameters	$\left[\begin{array}{c} 1.94\\ 1.01 \end{array}\right]$	5	$\left[\begin{array}{cc} 158.08 & -50.41\\ -50.41 & 49.38 \end{array}\right]$	$\left[\begin{array}{cc} 0.01 & 0\\ 0 & 0 \end{array}\right]$	5	520.59	$\left[\begin{array}{c} 4\\ 2 \end{array}\right]$	5	$\left[\begin{array}{cc} 10 & -5\\ -5 & 4 \end{array}\right]$	$\left[\begin{array}{cc} 0.10 & 0.05\\ 0.05 & 0.04 \end{array}\right]$	5	21.91
	Natural Gradient		$\left[\begin{array}{c} 1.94\\ 1.01 \end{array}\right]$	5	$\left[\begin{array}{cc} 158.08 & -50.41\\ -50.41 & 49.38 \end{array}\right]$	$\left[\begin{array}{cc} 0.01 & 0\\ 0 & 0 \end{array}\right]$	5	520.59	$\left[\begin{array}{c} 4\\ 2 \end{array}\right]$	5	$\left[\begin{array}{cc} 16.67 & -9.33\\ -9.33 & 6.67 \end{array}\right]$	$\left[\begin{array}{cc} 0.11 & 0.03\\ 0.03 & 0.06 \end{array}\right]$	5	11.67
	Initial Points →		$\left[\begin{array}{c} 3.35\\ -1.39\\ 2.25 \end{array}\right]$	5	$\left[\begin{array}{ccc} 857.08 & -388.16 & 553.55\\ -388.16 & 1041.13 & -310.01\\ 553.55 & -310.01 & 1127.20 \end{array}\right]$	$\left[\begin{array}{ccc} 0.08 & -0.04 & 0.05\\ -0.04 & 0.10 & -0.03\\ 0.05 & -0.03 & 0.11 \end{array}\right]$	5	781.55	$\left[\begin{array}{c} 4\\ -2\\ 3 \end{array}\right]$	5	$\left[\begin{array}{ccc} 9 & -6 & 6\\ -6 & 10 & -6\\ 6 & -6 & 6 \end{array}\right]$	$\left[\begin{array}{ccc} 0.09 & 0.06 & 0.06\\ 0.06 & 0.10 & 0.06\\ 0.06 & 0.06 & 0.06 \end{array}\right]$	5	23.53
	Alternative		$\left[\begin{array}{c} 3.35\\ -1.39\\ 2.25 \end{array}\right]$	5	$\left[\begin{array}{ccc} 8.16 & -3.70 & 5.27\\ -3.70 & 9.92 & -2.95\\ 5.27 & -2.95 & 10.74 \end{array}\right]$	$\left[\begin{array}{ccc} 16.57 & -7.50 & 10.70\\ -7.50 & 20.13 & -5.99\\ 10.70 & -5.99 & 21.79 \end{array}\right]$	5	9.05	$\left[\begin{array}{c} 3.19\\ -1.32\\ 2.15 \end{array}\right]$	5	$\left[\begin{array}{ccc} 0.09 & -0.06 & 0.06\\ -0.06 & 0.10 & -0.06\\ 0.06 & -0.06 & 0.06 \end{array}\right]$	$\left[\begin{array}{ccc} 8.18 & -3.62 & 5.29\\ -3.62 & 9.92 & -2.89\\ 5.29 & -2.89 & 10.64 \end{array}\right]$	5	15.89
		γ=1	$\left[\begin{array}{c} 3.35\\ -1.39\\ 2.25 \end{array}\right]$	5	$\left[\begin{array}{ccc} 857.08 & -388.16 & 553.55\\ -388.16 & 1041.13 & -310.01\\ 553.55 & -310.01 & 1127.20 \end{array}\right]$	$\left[\begin{array}{ccc} 0.08 & -0.04 & 0.05\\ -0.04 & 0.10 & -0.03\\ 0.05 & -0.03 & 0.11 \end{array}\right]$	5	781.55	$\left[\begin{array}{c} 4\\ -2\\ 3 \end{array}\right]$	5	$\left[\begin{array}{ccc} 9 & -6 & 6\\ -6 & 10 & -6\\ 6 & -6 & 6 \end{array}\right]$	$\left[\begin{array}{ccc} 0.09 & 0.06 & 0.06\\ 0.06 & 0.10 & 0.06\\ 0.06 & 0.06 & 0.06 \end{array}\right]$	5	23.53
$NIW\left(\tilde{\mu },\tilde{\Sigma }|\left[\begin{array}{c} 3\\ -1\\ 2 \end{array}\right],3,\left[\begin{array}{ccc} 3 & -1 & 2\\ -1 & 4 & -1\\ 2 & -1 & 5 \end{array}\right],4\right)$	Gradient	$\gamma =∥\nabla KL\left(\tilde{\Theta }\right){∥}^{-1}$	$\left[\begin{array}{c} 3.35\\ -1.39\\ 2.25 \end{array}\right]$	5	$\left[\begin{array}{ccc} 857.08 & -388.16 & 553.55\\ -388.16 & 1041.13 & -310.01\\ 553.55 & -310.01 & 1127.20 \end{array}\right]$	$\left[\begin{array}{ccc} 0.08 & -0.04 & 0.05\\ -0.04 & 0.10 & -0.03\\ 0.05 & -0.03 & 0.11 \end{array}\right]$	4.11	614.41	$\left[\begin{array}{c} 4\\ -2\\ 3 \end{array}\right]$	4.98	$\left[\begin{array}{ccc} 8.95 & -6 & 6.05\\ -6 & 9.96 & -5.94\\ 6.05 & -5.94 & 5.88 \end{array}\right]$	$\left[\begin{array}{ccc} 0.09 & 0.06 & 0.06\\ 0.06 & 0.10 & 0.06\\ 0.06 & 0.06 & 0.07 \end{array}\right]$	4.76	16.44
		Natural Parameters	$\left[\begin{array}{c} 3.35\\ -1.39\\ 2.25 \end{array}\right]$	5	$\left[\begin{array}{ccc} 857.08 & -388.16 & 553.55\\ -388.16 & 1041.13 & -310.01\\ 553.55 & -310.01 & 1127.2 \end{array}\right]$	$\left[\begin{array}{ccc} 0.08 & -0.04 & 0.05\\ -0.04 & 0.1 & -0.03\\ 0.05 & -0.03 & 0.11 \end{array}\right]$	5	781.55	$\left[\begin{array}{c} 4\\ -2\\ 3 \end{array}\right]$	5	$\left[\begin{array}{ccc} 9 & -6 & 6\\ -6 & 10 & -6\\ 6 & -6 & 6 \end{array}\right]$	$\left[\begin{array}{ccc} 0.09 & 0.06 & 0.06\\ 0.06 & 0.10 & 0.06\\ 0.06 & 0.06 & 0.06 \end{array}\right]$	5	23.53
	Natural Gradient		$\left[\begin{array}{c} 3.35\\ -1.39\\ 2.25 \end{array}\right]$	5	$\left[\begin{array}{ccc} 857.08 & -388.16 & 553.55\\ -388.16 & 1041.13 & -310.01\\ 553.55 & -310.01 & 1127.2 \end{array}\right]$	$\left[\begin{array}{ccc} 0.08 & -0.04 & 0.05\\ -0.04 & 0.1 & -0.03\\ 0.05 & -0.03 & 0.11 \end{array}\right]$	5	781.55	$\left[\begin{array}{c} 4\\ -2\\ 3 \end{array}\right]$	5	$\left[\begin{array}{ccc} 4 & -2 & 3\\ -10.8 & 16 & -10.8\\ 9.6 & -10.8 & 9.6 \end{array}\right]$	$\left[\begin{array}{ccc} 0.09 & 0.06 & 0.06\\ 0.06 & 0.11 & 0.05\\ 0.06 & 0.05 & 0.07 \end{array}\right]$	21.75	21.75
	Initial Points →		$\left[\begin{array}{c} -0.18\\ -0.29\\ 0.42\\ -0.23\\ -0.05 \end{array}\right]$	5	$\left[\begin{array}{ccccc} 473.25 & 152.04 & 66.00 & 31.17 & -58.19\\ 152.04 & 334.02 & 34.61 & -72.16 & 221.59\\ 66.00 & 34.61 & 947.32 & -90.75 & 16.37\\ 31.17 & -72.16 & -90.75 & 553.61 & -68.83\\ -58.19 & 221.59 & 16.37 & -68.83 & 545.75 \end{array}\right]$	$\left[\begin{array}{ccccc} 0.05 & 0.015 & 0.01 & 0 & -0.01\\ 0.015 & 0.032 & 0 & -0.01 & 0.02\\ 0.01 & 0 & 0.09 & -0.01 & 0\\ 0 & -0.01 & -0.01 & 0.05 & -0.01\\ -0.01 & 0.02 & 0 & -0.01 & 0.05 \end{array}\right]$	5	1304.77	$\left[\begin{array}{c} 0.5\\ 1\\ -0.5\\ 1\\ 0.5 \end{array}\right]$	5	$\left[\begin{array}{ccccc} 10 & 10 & 10 & 10 & -5\\ 10 & 10 & 10 & -10 & 10\\ 10 & 10 & 8 & -10 & 10\\ 10 & -10 & -10 & 10 & -6\\ -5 & 10 & 10 & -6 & 10 \end{array}\right]$	$\left[\begin{array}{ccccc} 0.10 & 0.10 & 0.10 & 0.10 & 0.05\\ 0.10 & 0.10 & 0.10 & -0.10 & 0.10\\ 0.10 & 0.10 & 0.08 & 0.10 & 0.10\\ 0.10 & -0.10 & 0.10 & 0.10 & -0.06\\ 0.05 & 0.10 & 0.10 & -0.06 & 0.10 \end{array}\right]$	5	21.85
	Alternative		$\left[\begin{array}{c} -0.18\\ -0.29\\ 0.42\\ -0.23\\ -0.05 \end{array}\right]$	5	$\left[\begin{array}{ccccc} 4.51 & 1.45 & 0.63 & 0.30 & -0.55\\ 1.45 & 3.18 & 0.33 & -0.69 & 2.11\\ 0.63 & 0.33 & 9.02 & -0.86 & 0.16\\ 0.30 & -0.69 & -0.86 & 5.27 & -0.66\\ -0.55 & 2.11 & 0.16 & -0.66 & 5.20 \end{array}\right]$	$\left[\begin{array}{ccccc} 9.33 & 2.30 & 1.30 & 0.61 & -1.15\\ 2.30 & 6.59 & 0.68 & -1.42 & 4.37\\ 1.30 & 0.68 & 18.68 & -1.79 & 0.32\\ 0.61 & -1.42 & -1.79 & 10.92 & -1.36\\ -1.15 & 4.37 & 0.32 & -1.36 & 10.76 \end{array}\right]$	5	17.28	$\left[\begin{array}{c} 0\\ 0\\ 0\\ 0\\ 0 \end{array}\right]$	5	$\left[\begin{array}{ccccc} 10 & 10 & 10 & 10 & -5\\ 10 & 10 & 10 & -10 & 10\\ 10 & 10 & 8 & -10 & 10\\ 10 & -10 & -10 & 10 & -6\\ -5 & 10 & 10 & -6 & 10 \end{array}\right]$	$\left[\begin{array}{ccccc} 0.10 & 0.10 & 0.10 & 0.10 & 0.05\\ 0.10 & 0.10 & 0.10 & -0.10 & 0.10\\ 0.10 & 0.10 & 0.18 & 0.10 & 0.10\\ 0.10 & -0.10 & 0.10 & 0.10 & 0.06\\ 0.05 & 0.10 & 0.10 & -0.06 & 0.10 \end{array}\right]$	5	21.85
		γ=1	$\left[\begin{array}{c} -0.18\\ -0.29\\ 0.42\\ -0.23\\ -0.05 \end{array}\right]$	5	$\left[\begin{array}{ccccc} 473.25 & 152.04 & 66.00 & 31.17 & -58.19\\ 152.04 & 334.02 & 34.61 & -72.16 & 221.59\\ 66.00 & 34.61 & 947.32 & -90.75 & 16.37\\ 31.17 & -72.16 & -90.75 & 553.61 & -68.83\\ -58.19 & 221.59 & 16.37 & -68.83 & 545.75 \end{array}\right]$	$\left[\begin{array}{ccccc} 0.05 & 0.01 & 0.01 & 0.00 & -0.01\\ 0.01 & 0.03 & 0.00 & -0.01 & 0.02\\ 0.01 & 0.00 & 0.09 & -0.01 & 0.00\\ 0.00 & 0.00 & -0.01 & 0.05 & -0.01\\ -0.01 & 0.02 & 0.00 & 0.00 & 0.05 \end{array}\right]$	5	1304.77	$\left[\begin{array}{c} 0.5\\ 1\\ -0.5\\ 1\\ 0.5 \end{array}\right]$	4.6	$\left[\begin{array}{ccccc} 10.00 & 9.64 & 9.91 & 10.00 & -4.55\\ 9.64 & 9.70 & 10.49 & -9.88 & 10.09\\ 9.91 & 10.49 & 7.92 & -10.11 & 9.57\\ 10.00 & -9.88 & -10.11 & 10.04 & -5.98\\ -4.55 & 10.09 & 9.57 & -5.98 & 9.91 \end{array}\right]$	$\left[\begin{array}{ccccc} 0.10 & 0.11 & 0.10 & 0.10 & 0.04\\ 0.11 & 0.10 & 0.09 & -0.10 & 0.10\\ 0.10 & 0.09 & 0.08 & 0.10 & 0.11\\ 0.10 & -0.10 & 0.10 & 0.10 & -0.06\\ 0.04 & 0.10 & 0.11 & -0.06 & 0.11 \end{array}\right]$	3.66	11.15
$NIW\left(\tilde{\mu },\tilde{\Sigma }|\left[\begin{array}{c} 0\\ 0\\ 0\\ 0\\ 0 \end{array}\right],4,\left[\begin{array}{ccccc} 6 & 2 & 1 & 1 & 0\\ 2 & 4 & 0 & -1 & 2\\ 1 & 0 & 9 & 1 & 0\\ 1 & -1 & 1 & 7 & 0\\ 0 & 2 & 0 & 0 & 5 \end{array}\right],7\right)$	Gradient	$\gamma =∥\nabla KL\left(\tilde{\Theta }\right){∥}^{-1}$	$\left[\begin{array}{c} -0.18\\ -0.29\\ 0.42\\ -0.23\\ -0.05 \end{array}\right]$	5	$\left[\begin{array}{ccccc} 473.25 & 152.04 & 66.00 & 31.17 & -58.19\\ 152.04 & 334.02 & 34.61 & -72.16 & 221.59\\ 66.00 & 34.61 & 947.32 & -90.75 & 16.37\\ 31.17 & -72.16 & -90.75 & 553.61 & -68.83\\ -58.19 & 221.59 & 16.37 & -68.83 & 545.75 \end{array}\right]$	$\left[\begin{array}{ccccc} 0.05 & 0.01 & 0.01 & 0.00 & 0.00\\ 0.01 & 0.04 & 0.00 & 0.00 & 0.02\\ 0.01 & 0.00 & 0.10 & 0.00 & 0.00\\ 0.00 & 0.00 & -0.01 & 0.06 & -0.01\\ 0.00 & 0.02 & 0.00 & -0.01 & 0.06 \end{array}\right]$	4.28	1018.67	$\left[\begin{array}{c} 0.5\\ 1\\ -0.5\\ 1\\ 0.5 \end{array}\right]$	4.88	$\left[\begin{array}{ccccc} 10.00 & 9.90 & 9.97 & 10.00 & -4.87\\ 9.90 & 9.91 & 10.14 & -9.96 & 10.03\\ 9.97 & 10.14 & 7.98 & -10.03 & 9.87\\ 10.00 & -9.96 & -10.03 & 10.01 & -5.99\\ -4.87 & 10.03 & 9.87 & -5.99 & 9.97 \end{array}\right]$	$\left[\begin{array}{ccccc} 0.10 & 0.10 & 0.10 & 0.10 & 0.05\\ 0.10 & 0.10 & -0.10 & 0.10 & 0.10\\ 0.10 & 0.10 & 0.08 & 0.10 & 0.10\\ 0.10 & -0.10 & 0.10 & 0.10 & -0.06\\ 0.05 & 0.10 & 0.10 & -0.06 & 0.10 \end{array}\right]$	4.71	19.13
		Natural Parameters	$\left[\begin{array}{c} -0.18\\ -0.29\\ 0.42\\ -0.23\\ -0.05 \end{array}\right]$	5	$\left[\begin{array}{ccccc} 473.25 & 152.04 & 66.00 & 31.17 & -58.19\\ 152.04 & 334.02 & 34.61 & -72.16 & 221.59\\ 66.00 & 34.61 & 947.32 & -90.75 & 16.37\\ 31.17 & -72.16 & -90.75 & 553.61 & -68.83\\ -58.19 & 221.59 & 16.37 & -68.83 & 545.75 \end{array}\right]$	$\left[\begin{array}{ccccc} 0.05 & 0.015 & 0.01 & 0 & -0.01\\ 0.015 & 0.032 & 0 & -0.01 & 0.02\\ 0.01 & 0 & 0.09 & -0.01 & 0\\ 0 & -0.01 & -0.01 & 0.05 & -0.01\\ -0.01 & 0.02 & 0 & -0.01 & 0.05 \end{array}\right]$	5	1304.77	$\left[\begin{array}{c} 0.5\\ 1\\ -0.5\\ 1\\ 0.5 \end{array}\right]$	5	$\left[\begin{array}{ccccc} 10 & 10 & 10 & 10 & -5\\ 10 & 10 & 10 & -10 & 10\\ 10 & 10 & 8 & -10 & 10\\ 10 & -10 & -10 & 10 & -6\\ -5 & 10 & 10 & -6 & 10 \end{array}\right]$	$\left[\begin{array}{ccccc} 0.10 & 0.10 & 0.10 & 0.10 & 0.05\\ 0.10 & 0.10 & 0.10 & -0.10 & 0.10\\ 0.10 & 0.10 & 0.08 & 0.10 & 0.10\\ 0.10 & -0.10 & 0.10 & 0.10 & -0.06\\ 0.05 & 0.10 & 0.10 & -0.06 & 0.10 \end{array}\right]$	5	21.85
	Natural Gradient		$\left[\begin{array}{c} -0.18\\ -0.29\\ 0.42\\ -0.23\\ -0.05 \end{array}\right]$	5	$\left[\begin{array}{ccccc} 473.25 & 152.04 & 66.00 & 31.17 & -58.19\\ 152.04 & 334.02 & 34.61 & -72.16 & 221.59\\ 66.00 & 34.61 & 947.32 & -90.75 & 16.37\\ 31.17 & -72.16 & -90.75 & 553.61 & -68.83\\ -58.19 & 221.59 & 16.37 & -68.83 & 545.75 \end{array}\right]$	$\left[\begin{array}{ccccc} 0.05 & 0.015 & 0.01 & 0 & -0.01\\ 0.015 & 0.032 & 0 & -0.01 & 0.02\\ 0.01 & 0 & 0.09 & -0.01 & 0\\ 0 & -0.01 & -0.01 & 0.05 & -0.01\\ -0.01 & 0.02 & 0 & -0.01 & 0.05 \end{array}\right]$	5	1304.77	$\left[\begin{array}{c} -0.18\\ -0.29\\ 0.42\\ -0.23\\ -0.05 \end{array}\right]$	5	$\left[\begin{array}{ccccc} 473.25 & 152.04 & 66.00 & 31.17 & -58.19\\ 152.04 & 334.02 & 34.61 & -72.16 & 221.59\\ 66.00 & 34.61 & 947.32 & -90.75 & 16.37\\ 31.17 & -72.16 & -90.75 & 553.61 & -68.83\\ -58.19 & 221.59 & 16.37 & -68.83 & 545.75 \end{array}\right]$	$\left[\begin{array}{ccccc} 0.05 & 0.015 & 0.01 & 0 & -0.01\\ 0.015 & 0.032 & 0 & -0.01 & 0.02\\ 0.01 & 0 & 0.09 & -0.01 & 0\\ 0 & -0.01 & -0.01 & 0.05 & -0.01\\ -0.01 & 0.02 & 0 & -0.01 & 0.05 \end{array}\right]$	5	1304.77

**Table 3:** We provide an example of running the algorithms on a large-scale dataset. The standard alternative algorithm is outstanding here.

Models	Algorithm↓		MLE Initializations					
			Parameters					KL(·)
			$\mu_{0}$	κ	Λ	$\frac{\Psi }{\nu +n-p-1}$	ν	
	Initial Points →		$\left[\begin{array}{c} 1\\ 2.02\\ 1.02\\ 3.05\\ -1.02\\ 4.05\\ -4.98\\ 10.03\\ 1.08\\ 0.03 \end{array}\right]$	5	$\left[\begin{array}{cccccccccc} 3.03 & 0.18 & -2.69 & 1.07 & 0.43 & 0.38 & 1.49 & 0.49 & 0.5 & -1.33\\ 0.18 & 4.73 & 2.56 & 1.57 & 3.75 & 4.62 & 3.12 & 3.48 & 3.49 & 1.02\\ -2.69 & 2.56 & 11.84 & -0.18 & 4.91 & 3.69 & 2.44 & 1.37 & 1.64 & 0.25\\ 1.07 & 1.57 & -0.18 & 6.67 & 2.21 & 4.75 & 2.65 & 1.87 & 4.21 & 0.16\\ 0.43 & 3.75 & 4.91 & 2.21 & 7.62 & 4.31 & 4.63 & 2.93 & 2.3 & 0.33\\ 0.38 & 4.62 & 3.69 & 4.75 & 4.31 & 8.59 & 4.52 & 4.69 & 7.47 & 1.78\\ 1.49 & 3.12 & 2.44 & 2.65 & 4.63 & 4.52 & 5.31 & 3.66 & 4.01 & 0.21\\ 0.49 & 3.48 & 1.37 & 1.87 & 2.93 & 4.69 & 3.66 & 7.73 & 4.21 & 5.67\\ 0.5 & 3.49 & 1.64 & 4.21 & 2.3 & 7.47 & 4.01 & 4.21 & 9.51 & 1.81\\ -1.33 & 1.02 & 0.25 & 0.16 & 0.33 & 1.78 & 0.21 & 5.67 & 1.81 & 8.67 \end{array}\right]$	$\left[\begin{array}{cccccccccc} 0.2 & 0.01 & -0.18 & 0.07 & 0.03 & 0.02 & 0.1 & 0.03 & 0.03 & -0.09\\ 0.01 & 0.31 & 0.17 & 0.1 & 0.25 & 0.3 & 0.21 & 0.23 & 0.23 & 0.07\\ -0.18 & 0.17 & 0.78 & -0.01 & 0.32 & 0.24 & 0.16 & 0.09 & 0.11 & 0.02\\ 0.07 & 0.1 & -0.01 & 0.44 & 0.15 & 0.31 & 0.17 & 0.12 & 0.28 & 0.01\\ 0.03 & 0.25 & 0.32 & 0.15 & 0.5 & 0.28 & 0.3 & 0.19 & 0.15 & 0.02\\ 0.02 & 0.3 & 0.24 & 0.31 & 0.28 & 0.56 & 0.3 & 0.31 & 0.49 & 0.12\\ 0.1 & 0.21 & 0.16 & 0.17 & 0.3 & 0.3 & 0.35 & 0.24 & 0.26 & 0.01\\ 0.03 & 0.23 & 0.09 & 0.12 & 0.19 & 0.31 & 0.24 & 0.51 & 0.28 & 0.37\\ 0.03 & 0.23 & 0.11 & 0.28 & 0.15 & 0.49 & 0.26 & 0.28 & 0.63 & 0.12\\ -0.09 & 0.07 & 0.02 & 0.01 & 0.02 & 0.12 & 0.01 & 0.37 & 0.12 & 0.57 \end{array}\right]$	10	5231.57
	Alternative		$\left[\begin{array}{c} 1.09\\ 2.44\\ 1.44\\ 4.19\\ -1.59\\ 5.2\\ -4.61\\ 10.81\\ 3.14\\ 0.79 \end{array}\right]$	5	$\left[\begin{array}{cccccccccc} 19.75 & 1.15 & -17.53 & 6.96 & 2.79 & 2.45 & 9.72 & 3.21 & 3.26 & -8.68\\ 1.15 & 30.8 & 16.64 & 10.21 & 24.44 & 30.1 & 20.3 & 22.69 & 22.69 & 6.63\\ -17.53 & 16.64 & 77.06 & -1.17 & 31.99 & 24.04 & 15.86 & 8.9 & 10.66 & 1.61\\ 6.96 & 10.21 & -1.17 & 43.41 & 14.38 & 30.95 & 17.28 & 12.16 & 27.39 & 1.05\\ 2.79 & 24.44 & 31.99 & 14.38 & 49.64 & 28.05 & 30.12 & 19.05 & 15 & 2.16\\ 2.45 & 30.1 & 24.04 & 30.95 & 28.05 & 55.9 & 29.45 & 30.51 & 48.65 & 11.62\\ 9.72 & 20.3 & 15.86 & 17.28 & 30.12 & 29.45 & 34.55 & 23.8 & 26.11 & 1.35\\ 3.21 & 22.69 & 8.9 & 12.16 & 19.05 & 30.51 & 23.8 & 50.35 & 27.43 & 36.89\\ 3.26 & 22.69 & 10.66 & 27.39 & 15 & 48.65 & 26.11 & 27.43 & 61.89 & 11.82\\ -8.68 & 6.63 & 1.61 & 1.05 & 2.16 & 11.62 & 1.35 & 36.89 & 11.82 & 56.43 \end{array}\right]$	$\left[\begin{array}{cccccccccc} 40.9 & 2.37 & -36.3 & 14.41 & 5.77 & 5.07 & 20.13 & 6.66 & 6.75 & -17.97\\ 2.37 & 63.77 & 34.45 & 21.13 & 50.62 & 62.33 & 42.03 & 46.98 & 46.99 & 13.72\\ -36.3 & 34.45 & 159.58 & -2.43 & 66.25 & 49.77 & 32.84 & 18.44 & 22.08 & 3.34\\ 14.41 & 21.13 & -2.43 & 89.89 & 29.77 & 64.08 & 35.79 & 25.18 & 56.72 & 2.17\\ 5.77 & 50.62 & 66.25 & 29.77 & 102.79 & 58.09 & 62.37 & 39.44 & 31.05 & 4.46\\ 5.07 & 62.33 & 49.77 & 64.08 & 58.09 & 115.75 & 60.97 & 63.17 & 100.74 & 24.06\\ 20.13 & 42.03 & 32.84 & 35.79 & 62.37 & 60.97 & 71.53 & 49.29 & 54.07 & 2.8\\ 6.66 & 46.98 & 18.44 & 25.18 & 39.44 & 63.17 & 49.29 & 104.26 & 56.79 & 76.38\\ 6.75 & 46.99 & 22.08 & 56.72 & 31.05 & 100.74 & 54.07 & 56.79 & 128.16 & 24.47\\ -17.97 & 13.72 & 3.34 & 2.17 & 4.46 & 24.06 & 2.8 & 76.38 & 24.47 & 116.85 \end{array}\right]$	10	31.70
		γ=1	$\left[\begin{array}{c} 1.09\\ 2.44\\ 1.44\\ 4.19\\ -1.59\\ 5.2\\ -4.61\\ 10.81\\ 3.14\\ 0.79 \end{array}\right]$	5	$\left[\begin{array}{cccccccccc} 2074.17 & 120.32 & -1840.86 & 730.59 & 292.65 & 257.14 & 1020.81 & 337.48 & 342.19 & -911.26\\ 120.32 & 3233.78 & 1746.98 & 1071.53 & 2566.57 & 3160.45 & 2131.31 & 2382.43 & 2382.86 & 695.73\\ -1840.86 & 1746.98 & 8091.77 & -123.13 & 3359.43 & 2523.71 & 1665.11 & 934.91 & 1119.54 & 169.52\\ 730.59 & 1071.53 & -123.13 & 4558.21 & 1509.46 & 3249.33 & 1814.84 & 1276.68 & 2875.88 & 109.91\\ 292.65 & 2566.57 & 3359.43 & 1509.46 & 5212.43 & 2945.44 & 3162.79 & 1999.9 & 1574.61 & 226.38\\ 257.14 & 3160.45 & 2523.71 & 3249.33 & 2945.44 & 5869.57 & 3091.85 & 3203.23 & 5108.31 & 1219.85\\ 1020.81 & 2131.31 & 1665.11 & 1814.84 & 3162.79 & 3091.85 & 3627.32 & 2499.28 & 2741.79 & 142.06\\ 337.48 & 2382.43 & 934.91 & 1276.68 & 1999.9 & 3203.23 & 2499.28 & 5286.96 & 2879.7 & 3873.26\\ 342.19 & 2382.86 & 1119.54 & 2875.88 & 1574.61 & 5108.31 & 2741.79 & 2879.7 & 6498.71 & 1240.72\\ -911.26 & 695.73 & 169.52 & 109.91 & 226.38 & 1219.85 & 142.06 & 3873.26 & 1240.72 & 5925.01 \end{array}\right]$	$\left[\begin{array}{cccccccccc} 0.2 & 0.01 & -0.18 & 0.07 & 0.03 & 0.02 & 0.1 & 0.03 & 0.03 & -0.09\\ 0.01 & 0.31 & 0.17 & 0.1 & 0.25 & 0.3 & 0.21 & 0.23 & 0.23 & 0.07\\ -0.18 & 0.17 & 0.78 & -0.01 & 0.32 & 0.24 & 0.16 & 0.09 & 0.11 & 0.02\\ 0.07 & 0.1 & -0.01 & 0.44 & 0.15 & 0.31 & 0.17 & 0.12 & 0.28 & 0.01\\ 0.03 & 0.25 & 0.32 & 0.15 & 0.5 & 0.28 & 0.3 & 0.19 & 0.15 & 0.02\\ 0.02 & 0.3 & 0.24 & 0.31 & 0.28 & 0.56 & 0.3 & 0.31 & 0.49 & 0.12\\ 0.1 & 0.21 & 0.16 & 0.17 & 0.3 & 0.3 & 0.35 & 0.24 & 0.26 & 0.01\\ 0.03 & 0.23 & 0.09 & 0.12 & 0.19 & 0.31 & 0.24 & 0.51 & 0.28 & 0.37\\ 0.03 & 0.23 & 0.11 & 0.28 & 0.15 & 0.49 & 0.26 & 0.28 & 0.63 & 0.12\\ -0.09 & 0.07 & 0.02 & 0.01 & 0.02 & 0.12 & 0.01 & 0.37 & 0.12 & 0.57 \end{array}\right]$	10	5231.57
$NIW\left(\tilde{\mu },\tilde{\Sigma }|\left[\begin{array}{c} 1\\ 2\\ 1\\ 3\\ -1\\ 4\\ -5\\ 10\\ 1\\ 0 \end{array}\right],10,\left[\begin{array}{cccccccccc} 67 & -11 & -50 & 16 & -4 & -2 & 21 & 21 & 10 & -16\\ -11 & 92 & 37 & 20 & 49 & 78 & 37 & 51 & 53 & 32\\ -50 & 37 & 177 & 14 & 53 & 76 & 41 & 29 & 61 & 9\\ 16 & 20 & 14 & 121 & 10 & 88 & 28 & 59 & 89 & 15\\ -4 & 49 & 53 & 10 & 114 & 57 & 53 & 44 & 30 & 26\\ -2 & 78 & 76 & 88 & 57 & 160 & 69 & 92 & 145 & 43\\ 21 & 37 & 41 & 28 & 53 & 69 & 71 & 60 & 73 & 11\\ 21 & 51 & 29 & 59 & 44 & 92 & 60 & 170 & 86 & 120\\ 10 & 53 & 61 & 89 & 30 & 145 & 73 & 86 & 196 & 33\\ -16 & 32 & 9 & 15 & 26 & 43 & 11 & 120 & 33 & 165 \end{array}\right],15\right)$	Gradient	$\gamma =∥\nabla KL\left(\tilde{\Theta }\right){∥}^{-1}$	$\left[\begin{array}{c} 1.09\\ 2.44\\ 1.44\\ 4.19\\ -1.59\\ 5.2\\ -4.61\\ 10.81\\ 3.14\\ 0.79 \end{array}\right]$	5	$\left[\begin{array}{cccccccccc} 2074.17 & 120.32 & -1840.86 & 730.59 & 292.65 & 257.14 & 1020.81 & 337.48 & 342.19 & -911.27\\ 120.32 & 3233.78 & 1746.98 & 1071.53 & 2566.57 & 3160.45 & 2131.31 & 2382.43 & 2382.86 & 695.73\\ -1840.86 & 1746.98 & 8091.77 & -123.13 & 3359.43 & 2523.71 & 1665.11 & 934.91 & 1119.54 & 169.52\\ 730.59 & 1071.53 & -123.13 & 4558.21 & 1509.46 & 3249.33 & 1814.84 & 1276.68 & 2875.88 & 109.91\\ 292.65 & 2566.57 & 3359.43 & 1509.46 & 5212.43 & 2945.44 & 3162.79 & 1999.9 & 1574.61 & 226.38\\ 257.14 & 3160.45 & 2523.71 & 3249.33 & 2945.44 & 5869.57 & 3091.85 & 3203.23 & 5108.31 & 1219.85\\ 1020.81 & 2131.31 & 1665.11 & 1814.84 & 3162.79 & 3091.85 & 3627.32 & 2499.28 & 2741.79 & 142.06\\ 337.48 & 2382.43 & 934.91 & 1276.68 & 1999.9 & 3203.23 & 2499.28 & 5286.96 & 2879.7 & 3873.26\\ 342.19 & 2382.86 & 1119.54 & 2875.88 & 1574.61 & 5108.31 & 2741.79 & 2879.7 & 6498.71 & 1240.72\\ -911.27 & 695.73 & 169.52 & 109.91 & 226.38 & 1219.85 & 142.06 & 3873.26 & 1240.72 & 5925.01 \end{array}\right]$	$\left[\begin{array}{cccccccccc} 0.2 & 0.01 & -0.18 & 0.07 & 0.03 & 0.02 & 0.1 & 0.03 & 0.03 & -0.09\\ 0.01 & 0.32 & 0.17 & 0.1 & 0.25 & 0.31 & 0.21 & 0.23 & 0.23 & 0.07\\ -0.18 & 0.17 & 0.79 & -0.01 & 0.33 & 0.24 & 0.16 & 0.09 & 0.11 & 0.02\\ 0.07 & 0.1 & -0.01 & 0.44 & 0.15 & 0.31 & 0.18 & 0.12 & 0.28 & 0.01\\ 0.03 & 0.25 & 0.33 & 0.15 & 0.51 & 0.29 & 0.31 & 0.19 & 0.15 & 0.02\\ 0.02 & 0.31 & 0.24 & 0.31 & 0.29 & 0.57 & 0.3 & 0.31 & 0.5 & 0.12\\ 0.1 & 0.21 & 0.16 & 0.18 & 0.31 & 0.3 & 0.35 & 0.24 & 0.27 & 0.01\\ 0.03 & 0.23 & 0.09 & 0.12 & 0.19 & 0.31 & 0.24 & 0.51 & 0.28 & 0.38\\ 0.03 & 0.23 & 0.11 & 0.28 & 0.15 & 0.5 & 0.27 & 0.28 & 0.63 & 0.12\\ -0.09 & 0.07 & 0.02 & 0.01 & 0.02 & 0.12 & 0.01 & 0.38 & 0.12 & 0.58 \end{array}\right]$	9.09	4681.41
		Natural Parameters	$\left[\begin{array}{c} 1\\ 2.02\\ 1.02\\ 3.05\\ -1.02\\ 4.05\\ -4.98\\ 10.03\\ 1.08\\ 0.03 \end{array}\right]$	5	$\left[\begin{array}{cccccccccc} 3.03 & 0.18 & -2.69 & 1.07 & 0.43 & 0.38 & 1.49 & 0.49 & 0.5 & -1.33\\ 0.18 & 4.73 & 2.56 & 1.57 & 3.75 & 4.62 & 3.12 & 3.48 & 3.49 & 1.02\\ -2.69 & 2.56 & 11.84 & -0.18 & 4.91 & 3.69 & 2.44 & 1.37 & 1.64 & 0.25\\ 1.07 & 1.57 & -0.18 & 6.67 & 2.21 & 4.75 & 2.65 & 1.87 & 4.21 & 0.16\\ 0.43 & 3.75 & 4.91 & 2.21 & 7.62 & 4.31 & 4.63 & 2.93 & 2.3 & 0.33\\ 0.38 & 4.62 & 3.69 & 4.75 & 4.31 & 8.59 & 4.52 & 4.69 & 7.47 & 1.78\\ 1.49 & 3.12 & 2.44 & 2.65 & 4.63 & 4.52 & 5.31 & 3.66 & 4.01 & 0.21\\ 0.49 & 3.48 & 1.37 & 1.87 & 2.93 & 4.69 & 3.66 & 7.73 & 4.21 & 5.67\\ 0.5 & 3.49 & 1.64 & 4.21 & 2.3 & 7.47 & 4.01 & 4.21 & 9.51 & 1.81\\ -1.33 & 1.02 & 0.25 & 0.16 & 0.33 & 1.78 & 0.21 & 5.67 & 1.81 & 8.67 \end{array}\right]$	$\left[\begin{array}{cccccccccc} 0.2 & 0.01 & -0.18 & 0.07 & 0.03 & 0.02 & 0.1 & 0.03 & 0.03 & -0.09\\ 0.01 & 0.31 & 0.17 & 0.1 & 0.25 & 0.3 & 0.21 & 0.23 & 0.23 & 0.07\\ -0.18 & 0.17 & 0.78 & -0.01 & 0.32 & 0.24 & 0.16 & 0.09 & 0.11 & 0.02\\ 0.07 & 0.1 & -0.01 & 0.44 & 0.15 & 0.31 & 0.17 & 0.12 & 0.28 & 0.01\\ 0.03 & 0.25 & 0.32 & 0.15 & 0.5 & 0.28 & 0.3 & 0.19 & 0.15 & 0.02\\ 0.02 & 0.3 & 0.24 & 0.31 & 0.28 & 0.56 & 0.3 & 0.31 & 0.49 & 0.12\\ 0.1 & 0.21 & 0.16 & 0.17 & 0.3 & 0.3 & 0.35 & 0.24 & 0.26 & 0.01\\ 0.03 & 0.23 & 0.09 & 0.12 & 0.19 & 0.31 & 0.24 & 0.51 & 0.28 & 0.37\\ 0.03 & 0.23 & 0.11 & 0.28 & 0.15 & 0.49 & 0.26 & 0.28 & 0.63 & 0.12\\ -0.09 & 0.07 & 0.02 & 0.01 & 0.02 & 0.12 & 0.01 & 0.37 & 0.12 & 0.57 \end{array}\right]$	10	5231.57
	Natural Gradient		$\left[\begin{array}{c} 1\\ 2.02\\ 1.02\\ 3.05\\ -1.02\\ 4.05\\ -4.98\\ 10.03\\ 1.08\\ 0.03 \end{array}\right]$	5	$\left[\begin{array}{cccccccccc} 3.03 & 0.18 & -2.69 & 1.07 & 0.43 & 0.38 & 1.49 & 0.49 & 0.5 & -1.33\\ 0.18 & 4.73 & 2.56 & 1.57 & 3.75 & 4.62 & 3.12 & 3.48 & 3.49 & 1.02\\ -2.69 & 2.56 & 11.84 & -0.18 & 4.91 & 3.69 & 2.44 & 1.37 & 1.64 & 0.25\\ 1.07 & 1.57 & -0.18 & 6.67 & 2.21 & 4.75 & 2.65 & 1.87 & 4.21 & 0.16\\ 0.43 & 3.75 & 4.91 & 2.21 & 7.62 & 4.31 & 4.63 & 2.93 & 2.3 & 0.33\\ 0.38 & 4.62 & 3.69 & 4.75 & 4.31 & 8.59 & 4.52 & 4.69 & 7.47 & 1.78\\ 1.49 & 3.12 & 2.44 & 2.65 & 4.63 & 4.52 & 5.31 & 3.66 & 4.01 & 0.21\\ 0.49 & 3.48 & 1.37 & 1.87 & 2.93 & 4.69 & 3.66 & 7.73 & 4.21 & 5.67\\ 0.5 & 3.49 & 1.64 & 4.21 & 2.3 & 7.47 & 4.01 & 4.21 & 9.51 & 1.81\\ -1.33 & 1.02 & 0.25 & 0.16 & 0.33 & 1.78 & 0.21 & 5.67 & 1.81 & 8.67 \end{array}\right]$	$\left[\begin{array}{cccccccccc} 0.2 & 0.01 & -0.18 & 0.07 & 0.03 & 0.02 & 0.1 & 0.03 & 0.03 & -0.09\\ 0.01 & 0.31 & 0.17 & 0.1 & 0.25 & 0.3 & 0.21 & 0.23 & 0.23 & 0.07\\ -0.18 & 0.17 & 0.78 & -0.01 & 0.32 & 0.24 & 0.16 & 0.09 & 0.11 & 0.02\\ 0.07 & 0.1 & -0.01 & 0.44 & 0.15 & 0.31 & 0.17 & 0.12 & 0.28 & 0.01\\ 0.03 & 0.25 & 0.32 & 0.15 & 0.5 & 0.28 & 0.3 & 0.19 & 0.15 & 0.02\\ 0.02 & 0.3 & 0.24 & 0.31 & 0.28 & 0.56 & 0.3 & 0.31 & 0.49 & 0.12\\ 0.1 & 0.21 & 0.16 & 0.17 & 0.3 & 0.3 & 0.35 & 0.24 & 0.26 & 0.01\\ 0.03 & 0.23 & 0.09 & 0.12 & 0.19 & 0.31 & 0.24 & 0.51 & 0.28 & 0.37\\ 0.03 & 0.23 & 0.11 & 0.28 & 0.15 & 0.49 & 0.26 & 0.28 & 0.63 & 0.12\\ -0.09 & 0.07 & 0.02 & 0.01 & 0.02 & 0.12 & 0.01 & 0.37 & 0.12 & 0.57 \end{array}\right]$	10	5231.57

**Table 4:** We have additional simulation results for the model g=Hf+ϵ with 4, 6, and 10 dimensions. The initializations are calculated based on some guesses using available data g. Although we do not need the values of $\mu_{f}$ and $v_{f}$ for the algorithm implementations, we have included two columns for them. The purpose of inverse problems is to extract f from the dataset of g. $v_{f}$ is a diagonal matrix, which we represent by its main diagonal components as a vector here.

Models	Algorithm↓		Data-Based Initializations					
			Parameters					KL(·)
			α	β	$v_{\epsilon }$	$\mu_{f}$	$v_{f}$	
	Initial Points →		$\left[\begin{array}{c} 3.08\\ 3.13\\ 2.7\\ 3.13 \end{array}\right]$	$\left[\begin{array}{c} 2.6\\ 1.98\\ 5.35\\ 2.32 \end{array}\right]$	3.06	−	−	212.55
	Alternative		$\left[\begin{array}{c} 3.08\\ 3.13\\ 2.7\\ 3.13 \end{array}\right]$	$\left[\begin{array}{c} 3.92\\ 2.99\\ 8.17\\ 3.5 \end{array}\right]$	3.06	$\left[\begin{array}{c} -0.02\\ -0.07\\ 0.36\\ -0.07 \end{array}\right]$	$\left[\begin{array}{c} 0.03\\ 0.03\\ 0.03\\ 0.03 \end{array}\right]$	12.37
		γ=1	$\left[\begin{array}{c} 3.08\\ 3.13\\ 2.7\\ 3.13 \end{array}\right]$	$\left[\begin{array}{c} 2.6\\ 1.98\\ 5.35\\ 2.32 \end{array}\right]$	3.06	$\left[\begin{array}{c} -0.02\\ -0.07\\ 0.37\\ -0.07 \end{array}\right]$	$\left[\begin{array}{c} 2.6\\ 1.98\\ 5.35\\ 2.32 \end{array}\right]$	212.55
$\alpha =\left[\begin{array}{c} 7\\ 4\\ 3\\ 5 \end{array}\right],\beta =\left[\begin{array}{c} 6\\ 3\\ 8\\ 4 \end{array}\right],v_{\epsilon }=,\mu_{f}=0,v_{f}=\left[\begin{array}{c} 1.23\\ 0.82\\ 3.23\\ 1.36 \end{array}\right]$	Gradient	$\gamma =∥KL\left(\tilde{\theta }\right){∥}^{-1}$	$\left[\begin{array}{c} 3.01\\ 3.07\\ 2.6\\ 3.06 \end{array}\right]$	$\left[\begin{array}{c} 2.89\\ 2.22\\ 5.68\\ 2.59 \end{array}\right]$	6.5	0	$\left[\begin{array}{c} 0.89\\ 0.24\\ 3.71\\ 0.6 \end{array}\right]$	46.37
		Natural Parameters	$\left[\begin{array}{c} 3.08\\ 3.13\\ 2.7\\ 3.13 \end{array}\right]$	$\left[\begin{array}{c} 2.6\\ 1.98\\ 5.35\\ 2.32 \end{array}\right]$	3.06	$\left[\begin{array}{c} -0.02\\ -0.07\\ 0.37\\ -0.07 \end{array}\right]$	$\left[\begin{array}{c} 2.6\\ 1.98\\ 5.35\\ 2.32 \end{array}\right]$	212.55
	Natural Gradient		$\left[\begin{array}{c} 3.08\\ 3.13\\ 2.7\\ 3.13 \end{array}\right]$	$\left[\begin{array}{c} 2.6\\ 1.98\\ 5.35\\ 2.32 \end{array}\right]$	3.06	$\left[\begin{array}{c} -0.02\\ -0.07\\ 0.37\\ -0.07 \end{array}\right]$	$\left[\begin{array}{c} 2.6\\ 1.98\\ 5.35\\ 2.32 \end{array}\right]$	212.55
	Initial Points →		$\left[\begin{array}{c} 3.58\\ 3.64\\ 3.91\\ 3.41\\ 3.65\\ 3.52 \end{array}\right]$	$\left[\begin{array}{c} 1.52\\ 2.89\\ 4.77\\ 7.56\\ 2.86\\ 2.39 \end{array}\right]$	3.67	−	−	320.4
	Alternative		$\left[\begin{array}{c} 3.58\\ 3.64\\ 3.91\\ 3.41\\ 3.65\\ 3.52 \end{array}\right]$	$\left[\begin{array}{c} 2.3\\ 4.36\\ 7.23\\ 11.43\\ 4.32\\ 3.63 \end{array}\right]$	3.67	$\left[\begin{array}{c} 0.08\\ 0.03\\ -0.23\\ 0.26\\ 0.02\\ 0.13 \end{array}\right]$	$\left[\begin{array}{c} 0.03\\ 0.04\\ 0.04\\ 0.04\\ 0.04\\ 0.04 \end{array}\right]$	16.38
		γ=1	$\left[\begin{array}{c} 3.58\\ 3.64\\ 3.91\\ 3.41\\ 3.65\\ 3.52 \end{array}\right]$	$\left[\begin{array}{c} 1.52\\ 2.89\\ 4.77\\ 7.56\\ 2.86\\ 2.39 \end{array}\right]$	3.67	$\left[\begin{array}{c} 0.09\\ 0.03\\ -0.24\\ 0.26\\ 0.02\\ 0.14 \end{array}\right]$	$\left[\begin{array}{c} 1.52\\ 2.89\\ 4.77\\ 7.56\\ 2.86\\ 2.39 \end{array}\right]$	320.4
$\alpha =\left[\begin{array}{c} 6\\ 4\\ 3\\ 2\\ 3\\ 5 \end{array}\right],\beta =\left[\begin{array}{c} 4\\ 5\\ 6\\ 8\\ 3\\ 7 \end{array}\right],v_{\epsilon }=,\mu_{f}=0,v_{f}=\left[\begin{array}{c} 0.73\\ 1.91\\ 3.61\\ 7.41\\ 1.3\\ 1.41 \end{array}\right]$	Gradient	$\gamma =∥KL\left(\tilde{\theta }\right){∥}^{-1}$	$\left[\begin{array}{c} 3.52\\ 3.56\\ 3.82\\ 3.32\\ 3.57\\ 3.46 \end{array}\right]$	$\left[\begin{array}{c} 1.73\\ 3.21\\ 5.16\\ 7.92\\ 3.18\\ 2.68 \end{array}\right]$	7.58	$\left[\begin{array}{c} 0.01\\ 0\\ -0.01\\ 0.02\\ 0\\ 0.01 \end{array}\right]$	$\left[\begin{array}{c} 0.27\\ 1.72\\ 3.63\\ 6.46\\ 1.69\\ 1.2 \end{array}\right]$	108.9
		Natural Parameters	$\left[\begin{array}{c} 3.58\\ 3.64\\ 3.91\\ 3.41\\ 3.65\\ 3.52 \end{array}\right]$	$\left[\begin{array}{c} 1.52\\ 2.89\\ 4.77\\ 7.56\\ 2.86\\ 2.39 \end{array}\right]$	3.67	$\left[\begin{array}{c} 0.09\\ 0.03\\ -0.24\\ 0.26\\ 0.02\\ 0.14 \end{array}\right]$	$\left[\begin{array}{c} 1.52\\ 2.89\\ 4.77\\ 7.56\\ 2.86\\ 2.39 \end{array}\right]$	320.4
	Natural Gradient		$\left[\begin{array}{c} 3.58\\ 3.64\\ 3.91\\ 3.41\\ 3.65\\ 3.52 \end{array}\right]$	$\left[\begin{array}{c} 1.52\\ 2.89\\ 4.77\\ 7.56\\ 2.86\\ 2.39 \end{array}\right]$	3.67	$\left[\begin{array}{c} 0.09\\ 0.03\\ -0.24\\ 0.26\\ 0.02\\ 0.14 \end{array}\right]$	$\left[\begin{array}{c} 1.52\\ 2.89\\ 4.77\\ 7.56\\ 2.86\\ 2.39 \end{array}\right]$	320.4
	Initial Points →		$\left[\begin{array}{c} 3.89\\ 3.41\\ 3.66\\ 3.76\\ 3.55\\ 3.9\\ 3.88\\ 3.8\\ 3.51\\ 3.95 \end{array}\right]$	$\left[\begin{array}{c} 2.04\\ 3.79\\ 2.27\\ 7.35\\ 12.92\\ 2.9\\ 1.36\\ 1.33\\ 1.35\\ 2.7 \end{array}\right]$	3.8	−	−	536.28
	Alternative		$\left[\begin{array}{c} 3.89\\ 3.41\\ 3.66\\ 3.76\\ 3.55\\ 3.9\\ 3.88\\ 3.8\\ 3.51\\ 3.95 \end{array}\right]$	$\left[\begin{array}{c} 3.08\\ 5.85\\ 3.44\\ 11.04\\ 19.46\\ 4.38\\ 2.06\\ 2.01\\ 2.13\\ 4.09 \end{array}\right]$	3.8	$\left[\begin{array}{c} -0.08\\ 0.38\\ 0.13\\ 0.04\\ 0.25\\ -0.1\\ -0.07\\ 0\\ 0.26\\ -0.14 \end{array}\right]$	$\left[\begin{array}{c} 0.04\\ 0.04\\ 0.04\\ 0.04\\ 0.04\\ 0.04\\ 0.04\\ 0.04\\ 0.04\\ 0.04 \end{array}\right]$	27.43
		γ=1	$\left[\begin{array}{c} 3.89\\ 3.41\\ 3.66\\ 3.76\\ 3.55\\ 3.9\\ 3.88\\ 3.8\\ 3.51\\ 3.95 \end{array}\right]$	$\left[\begin{array}{c} 2.04\\ 3.79\\ 2.27\\ 7.35\\ 12.92\\ 2.9\\ 1.36\\ 1.33\\ 1.35\\ 2.7 \end{array}\right]$	3.8	$\left[\begin{array}{c} -0.09\\ 0.39\\ 0.14\\ 0.04\\ 0.25\\ -0.1\\ -0.08\\ 0\\ 0.29\\ -0.15 \end{array}\right]$	$\left[\begin{array}{c} 2.04\\ 3.79\\ 2.27\\ 7.35\\ 12.92\\ 2.9\\ 1.36\\ 1.33\\ 1.35\\ 2.7 \end{array}\right]$	536.28
$\alpha =\left[\begin{array}{c} 6\\ 2\\ 3\\ 2\\ 1\\ 5\\ 5\\ 4\\ 4\\ 3 \end{array}\right],\beta =\left[\begin{array}{c} 4\\ 5\\ 2\\ 6\\ 3\\ 7\\ 2\\ 1\\ 1\\ 3 \end{array}\right],v_{\epsilon }=,\mu_{f}=0,v_{f}=\left[\begin{array}{c} 0.91\\ 3.51\\ 1.2\\ 6.76\\ 12.4\\ 2.2\\ 0.41\\ 0.33\\ 0.36\\ 1.27 \end{array}\right]$	Gradient	$\gamma =∥KL\left(\tilde{\theta }\right){∥}^{-1}$	$\left[\begin{array}{c} 3.81\\ 3.29\\ 3.57\\ 3.62\\ 3.41\\ 3.8\\ 3.84\\ 3.76\\ 3.47\\ 3.85 \end{array}\right]$	$\left[\begin{array}{c} 2.41\\ 4.25\\ 2.65\\ 7.94\\ 13.52\\ 3.38\\ 1.58\\ 1.53\\ 1.55\\ 3.16 \end{array}\right]$	10.04	$\left[\begin{array}{c} -0\\ 0.02\\ 0.01\\ 0\\ 0.01\\ 0\\ 0\\ 0\\ 0.01\\ -0.01 \end{array}\right]$	$\left[\begin{array}{c} 0.51\\ 2.38\\ 0.77\\ 5.99\\ 11.59\\ 1.43\\ 0.05\\ 0.19\\ 0\\ 1.22 \end{array}\right]$	136.63
		Natural Parameters	$\left[\begin{array}{c} 3.89\\ 3.41\\ 3.66\\ 3.76\\ 3.55\\ 3.9\\ 3.88\\ 3.8\\ 3.51\\ 3.95 \end{array}\right]$	$\left[\begin{array}{c} 2.04\\ 3.79\\ 2.27\\ 7.35\\ 12.92\\ 2.9\\ 1.36\\ 1.33\\ 1.35\\ 2.7 \end{array}\right]$	3.8	$\left[\begin{array}{c} -0.09\\ 0.39\\ 0.14\\ 0.04\\ 0.25\\ -0.1\\ -0.08\\ 0\\ 0.29\\ -0.15 \end{array}\right]$	$\left[\begin{array}{c} 2.04\\ 3.79\\ 2.27\\ 7.35\\ 12.92\\ 2.9\\ 1.36\\ 1.33\\ 1.35\\ 2.7 \end{array}\right]$	536.28
	Natural Gradient		$\left[\begin{array}{c} 3.89\\ 3.41\\ 3.66\\ 3.76\\ 3.55\\ 3.9\\ 3.88\\ 3.8\\ 3.51\\ 3.95 \end{array}\right]$	$\left[\begin{array}{c} 2.04\\ 3.79\\ 2.27\\ 7.35\\ 12.92\\ 2.9\\ 1.36\\ 1.33\\ 1.35\\ 2.7 \end{array}\right]$	3.8	$\left[\begin{array}{c} -0.09\\ 0.39\\ 0.14\\ 0.04\\ 0.25\\ -0.1\\ -0.08\\ 0\\ 0.29\\ -0.15 \end{array}\right]$	$\left[\begin{array}{c} 2.04\\ 3.79\\ 2.27\\ 7.35\\ 12.92\\ 2.9\\ 1.36\\ 1.33\\ 1.35\\ 2.7 \end{array}\right]$	536.28

## Data Availability

The data presented in this study are available on request from the corresponding author.

## Appendix A. Computation of KL($\tilde{\theta }$) and its Gradient for Normal-Inverse-Gamma

The mathematical computations of $KL(\tilde{\theta })$ in Section 3 is based on (5) as follows:

(A1) $$\begin{array}{cc} KL(q_{1}q_{2}|p)= & -H\left(q_{1}\right)-H\left(q_{2}\right)-\int \int q_{1}\left(x\right)q_{2}\left(y\right)\ln\left\{\frac{1}{\sqrt{2\pi y}}\right.\\ \; & \;\left.\exp\left\{-\frac{{\left(x-\tilde{\mu }\right)}^{2}}{2y}\right\}\frac{{\tilde{\beta }}^{\tilde{\alpha }}}{\Gamma (\tilde{\alpha })}y^{-(\tilde{\alpha }+1)}\exp\left\{-\frac{\tilde{\beta }}{y}\right\}\right\}dxdy\\ = & -H\left(q_{1}\right)-H\left(q_{2}\right)-\int \int q_{1}\left(x\right)q_{2}\left(y\right)\left\{-\frac{1}{2}\ln\;\left(2\pi \right)\right.\\ \; & \;\left.-\frac{{\left(x-\tilde{\mu }\right)}^{2}}{2y}+\tilde{\alpha }\ln\;\tilde{\beta }-\ln\;\Gamma \left(\tilde{\alpha }\right)-\left(\tilde{\alpha }+\frac{3}{2}\right)\;\ln\;y-\frac{\tilde{\beta }}{y}\right\}dxdy. \end{array}$$

Since, $H(q_{1})$ and $H(q_{2})$ are Shannon entropy, we have:

(A2) $$\begin{array}{cc} -H(q_{1})= & \int q_{1}\left(x\right)\ln\;\left(\frac{1}{\sqrt{2\pi \tilde{v}}}\exp\left\{-\frac{{\left(x-{\tilde{\mu }}^{\prime }\right)}^{2}}{2\tilde{v}}\right\}\right)dx\\ = & -\frac{1}{2}\ln\;\left(2\pi \tilde{v}\right)-\frac{1}{2}, \end{array}$$

and

(A3) $$\begin{array}{cc} -H(q_{2})= & \int q_{2}\left(y\right)\ln\;\left(\frac{{\tilde{\beta }}^{\tilde{\alpha }}}{\Gamma (\tilde{\alpha })}y^{-(\tilde{\alpha }+1)}\exp\left\{-\frac{\tilde{\beta }}{y}\right\}\right)dy\\ = & -\tilde{\alpha }-\ln\;\left(\tilde{\beta }\Gamma \left(\tilde{\alpha }\right)\right)+\left(\tilde{\alpha }+1\right)\psi_{0}\left(\tilde{\alpha }\right). \end{array}$$

Also, we need to know $-{\langle \ln\;p\left(x,y\right)\rangle }_{q_{1}q_{2}}$ as well:

(A4) $$\begin{array}{cc} -{\langle \ln\;p\left(x,y\right)\rangle }_{q_{1}q_{2}}= & \int \int q_{1}\left(x\right)q_{2}\left(y\right)\left(-\frac{1}{2}\ln\;\left(2\pi \right)\right.\\ \; & \left.-\frac{{\left(x-\tilde{\mu }\right)}^{2}}{2y}+\tilde{\alpha }\ln\;\tilde{\beta }-\ln\;\Gamma \left(\tilde{\alpha }\right)-\left(\tilde{\alpha }+\frac{3}{2}\right)\;\ln\;y-\frac{\tilde{\beta }}{y}\right)dxdy\\ = & \frac{1}{2}\ln\;\left(2\pi \right)-\tilde{\alpha }\;\ln\;\tilde{\beta }+\ln\;\Gamma \left(\tilde{\alpha }\right)+\left(\tilde{\alpha }+\frac{3}{2}\right)\left(\ln\;\tilde{\beta }-\psi_{0}\left(\tilde{\alpha }\right)\right)\\ \; & +\left(\frac{\tilde{v}+{\left(\tilde{\mu }-{\tilde{\mu }}^{\prime }\right)}^{2}}{2}+\tilde{\beta }\right)\frac{\tilde{\alpha }}{\tilde{\beta }}. \end{array}$$

Thus, the desire function $KL(\tilde{\theta })$ is:

(A5) $$\begin{array}{cc} KL(\tilde{\theta })\propto & \ln\Gamma \left(\tilde{\alpha }\right)-\frac{1}{2}\ln\tilde{v}-\ln\left(\tilde{\beta }\Gamma \left(\tilde{\alpha }\right)\right)+\left(\tilde{\alpha }+\frac{3}{2}\right)\left(\ln\;\tilde{\beta }-\psi_{0}\left(\tilde{\alpha }\right)\right)\\ \; & -\tilde{\alpha }\ln\tilde{\beta }+\left(\tilde{\alpha }+1\right)\psi_{0}\left(\tilde{\alpha }\right)+\frac{\tilde{\alpha }\left(\tilde{v}+{\left(\tilde{\mu }-{\tilde{\mu }}^{\prime }\right)}^{2}\right)}{2\tilde{\beta }}\\ \propto & -\frac{1}{2}\ln\tilde{v}+\frac{1}{2}\left(\ln\;\tilde{\beta }-\psi_{0}\left(\tilde{\alpha }\right)\right)+\frac{\tilde{\alpha }\left(\tilde{v}+{\left(\tilde{\mu }-{\tilde{\mu }}^{\prime }\right)}^{2}\right)}{2\tilde{\beta }}, \end{array}$$

where, $\psi_{0}\left(\cdot \right)$ is the polygamma function of order 0, or called digamma function. The gradient expression concerning $\tilde{\theta }=\left(\tilde{\alpha },\tilde{\beta },{\tilde{\mu }}^{\prime },\tilde{v}\right)$ is:

(A6) $$\begin{array}{cc} \nabla KL(\tilde{\theta })= & \left(\frac{\tilde{v}+{\left(\tilde{\mu }-{\tilde{\mu }}^{\prime }\right)}^{2}-\tilde{\beta }\psi_{1}\left(\tilde{\alpha }\right)}{2\tilde{\beta }},\;\frac{-\tilde{\alpha }\left(\tilde{v}+{\left(\tilde{\mu }-{\tilde{\mu }}^{\prime }\right)}^{2}\right)+\tilde{\beta }}{2{\tilde{\beta }}^{2}},\right.\\ \; & \;\left.\frac{\tilde{\alpha }\left({\tilde{\mu }}^{\prime }-\tilde{\mu }\right)}{\tilde{\beta }},\;\frac{\tilde{\alpha }}{2\tilde{\beta }}-\frac{1}{2\tilde{v}}\right), \end{array}$$

where, $\psi_{1}\left(\cdot \right)$ is the polygamma function of order 1. We substitute (A6) in the gradient-based formulas, so the algorithm is as follows:

2. The gradient-based algorithm with normal parameters:

$$\begin{array}{ccc} {\tilde{\alpha }}^{\left(k+1\right)} & = & {\tilde{\alpha }}^{\left(k\right)}+\gamma \frac{\partial KL}{\partial \tilde{\alpha }}\left({\tilde{\alpha }}^{\left(k\right)},{\tilde{\beta }}^{\left(k\right)},{\tilde{\mu }}^{\prime \left(k\right)},{\tilde{v}}^{\left(k\right)}\right)\\ & = & {\tilde{\alpha }}^{\left(k\right)}-\gamma \left(\frac{{\tilde{v}}^{\left(k\right)}+{\left(\tilde{\mu }-{\tilde{\mu }}^{\prime \left(k\right)}\right)}^{2}-{\tilde{\beta }}^{\left(k\right)}\psi_{1}\left({\tilde{\alpha }}^{\left(k\right)}\right)}{2{\tilde{\beta }}^{\left(k\right)}}\right),\\ {\tilde{\beta }}^{\left(k+1\right)} & = & {\tilde{\beta }}^{\left(k\right)}-\gamma \frac{\partial KL}{\partial \tilde{\beta }}\left({\tilde{\alpha }}^{\left(k+1\right)},{\tilde{\beta }}^{\left(k\right)},{\tilde{\mu }}^{\prime \left(k\right)},{\tilde{v}}^{\left(k\right)}\right)\\ & = & {\tilde{\beta }}^{\left(k\right)}-\gamma \left(\frac{-{\tilde{\alpha }}^{\left(k+1\right)}\left({\tilde{v}}^{\left(k\right)}+{\left(\tilde{\mu }-{\tilde{\mu }}^{\prime \left(k\right)}\right)}^{2}\right)+{\tilde{\beta }}^{\left(k\right)}}{2{\left[{\tilde{\beta }}^{\left(k\right)}\right]}^{2}}\right),\\ {\tilde{\mu }}^{\prime \left(k+1\right)} & = & {\tilde{\mu }}^{\prime \left(k\right)}-\gamma \frac{\partial KL}{\partial \tilde{\mu }}\left({\tilde{\alpha }}^{\left(k+1\right)},{\tilde{\beta }}^{\left(k+1\right)},{\tilde{\mu }}^{\prime \left(k\right)},{\tilde{v}}^{\left(k\right)}\right)\\ & = & {\tilde{\mu }}^{\prime \left(k\right)}-\gamma \left(\frac{{\tilde{\alpha }}^{\left(k+1\right)}\left({\tilde{\mu }}^{\prime \left(k\right)}-\tilde{\mu }\right)}{{\tilde{\beta }}^{\left(k+1\right)}}\right),\\ {\tilde{v}}^{\left(k+1\right)} & = & {\tilde{v}}^{\left(k\right)}-\gamma \frac{\partial KL}{\partial \tilde{v}}\left({\tilde{\alpha }}^{\left(k+1\right)},{\tilde{\beta }}^{\left(k+1\right)},{\tilde{\mu }}^{\prime \left(k+1\right)},{\tilde{v}}^{\left(k\right)}\right)\\ & = & {\tilde{v}}^{\left(k+1\right)}-\gamma \left(\frac{{\tilde{\alpha }}^{\left(k+1\right)}}{2{\tilde{\beta }}^{\left(k+1\right)}}-\frac{1}{2{\tilde{v}}^{\left(k\right)}}\right), \end{array}$$

where γ is a fix value. For the natural gradient algorithm, we need to calculate the gradient of $\ln p(x,y|\tilde{\theta })$ in (9). Remember, we change the notation p(x,y) to $p(x,y|\tilde{\theta })$ for the natural gradient algorithm explained in (2). Since Equation (9) is a function of $\tilde{\alpha }$, $\tilde{\beta }$, and ${\tilde{\mu }}^{\prime }$, the final algorithm has no information about $\tilde{v}$. Therefore, we replace $\ln p(x,y|\tilde{\theta })$ with $\ln q(x,y|\tilde{\theta })$:

(A7) $$\begin{array}{cc} \ln q(x,y|\tilde{\theta })= & \ln\left(\frac{{\tilde{\beta }}^{\tilde{\alpha }}}{\Gamma (\tilde{\alpha })}y^{-(\tilde{\alpha }+1)}\exp\left\{-\frac{\tilde{\beta }}{y}\right\}\frac{1}{\sqrt{2\pi \tilde{v}}}\exp\left\{-\frac{{\left(x-{\tilde{\mu }}^{\prime }\right)}^{2}}{2\tilde{v}}\right\}\right)\\ = & \tilde{\alpha }\ln\tilde{\beta }-\ln\Gamma \left(\tilde{\alpha }\right)-\left(\tilde{\alpha }+1\right)\ln y-\frac{\tilde{\beta }}{y}-\frac{1}{2}\ln\tilde{v}\\ \; & -\frac{1}{2}\ln\left(2\pi \right)-\frac{{\left(x-{\tilde{\mu }}^{\prime }\right)}^{2}}{2\tilde{v}}. \end{array}$$

So, we obtain the following Fisher information matrix by using $\ln q(x,y|\tilde{\theta })$:

$$\bar{F}=\langle \left(\begin{array}{cccc} {\left(\ln\tilde{\beta }-\psi_{0}\left(\tilde{\alpha }\right)-\ln y\right)}^{2} & \left(\ln\tilde{\beta }-\psi_{0}\left(\tilde{\alpha }\right)-\ln y\right)\left(\frac{\tilde{\alpha }}{\tilde{\beta }}-\frac{1}{y}\right) & \left(\ln\tilde{\beta }-\psi_{0}\left(\tilde{\alpha }\right)-\ln y\right)\frac{\left(x-{\tilde{\mu }}^{\prime }\right)}{\tilde{v}} & \left(\ln\tilde{\beta }-\psi_{0}\left(\tilde{\alpha }\right)-\ln y\right)\left(\frac{{\left(x-{\tilde{\mu }}^{\prime }\right)}^{2}}{2{\tilde{v}}^{2}}-\frac{1}{2\tilde{v}}\right)\\ \left(\ln\tilde{\beta }-\psi_{0}\left(\tilde{\alpha }\right)-\ln y\right)\left(\frac{\tilde{\alpha }}{\tilde{\beta }}-\frac{1}{y}\right) & {\left(\frac{\tilde{\alpha }}{\tilde{\beta }}-\frac{1}{y}\right)}^{2} & \left(\frac{\tilde{\alpha }}{\tilde{\beta }}-\frac{1}{y}\right)\frac{\left(x-{\tilde{\mu }}^{\prime }\right)}{\tilde{v}} & \left(\frac{\tilde{\alpha }}{\tilde{\beta }}-\frac{1}{y}\right)\left(\frac{{\left(x-{\tilde{\mu }}^{\prime }\right)}^{2}}{2{\tilde{v}}^{2}}-\frac{1}{2\tilde{v}}\right)\\ \left(\ln\tilde{\beta }-\psi_{0}\left(\tilde{\alpha }\right)-\ln y\right)\frac{\left(x-{\tilde{\mu }}^{\prime }\right)}{\tilde{v}} & \left(\frac{\tilde{\alpha }}{\tilde{\beta }}-\frac{1}{y}\right)\frac{\left(x-{\tilde{\mu }}^{\prime }\right)}{\tilde{v}} & \frac{{\left(x-{\tilde{\mu }}^{\prime }\right)}^{2}}{{\tilde{v}}^{2}} & \frac{\left(x-{\tilde{\mu }}^{\prime }\right)}{\tilde{v}}\left(\frac{{\left(x-{\tilde{\mu }}^{\prime }\right)}^{2}}{2{\tilde{v}}^{2}}-\frac{1}{2\tilde{v}}\right)\\ \left(\ln\tilde{\beta }-\psi_{0}\left(\tilde{\alpha }\right)-\ln y\right)\left(\frac{{\left(x-{\tilde{\mu }}^{\prime }\right)}^{2}}{2{\tilde{v}}^{2}}-\frac{1}{2\tilde{v}}\right) & \left(\frac{\tilde{\alpha }}{\tilde{\beta }}-\frac{1}{y}\right)\left(\frac{{\left(x-{\tilde{\mu }}^{\prime }\right)}^{2}}{2{\tilde{v}}^{2}}-\frac{1}{2\tilde{v}}\right) & \frac{\left(x-{\tilde{\mu }}^{\prime }\right)}{\tilde{v}}\left(\frac{{\left(x-{\tilde{\mu }}^{\prime }\right)}^{2}}{2{\tilde{v}}^{2}}-\frac{1}{2\tilde{v}}\right) & {\left(\frac{{\left(x-{\tilde{\mu }}^{\prime }\right)}^{2}}{2{\tilde{v}}^{2}}-\frac{1}{2\tilde{v}}\right)}^{2} \end{array}\right)\rangle_{q(x,y)}.$$

After integrating over $q(x,y|\tilde{\theta })$, we have:

$$\bar{F}=\left(\begin{array}{cccc} \psi_{1}\left(\tilde{\alpha }\right) & -\frac{1}{\tilde{\beta }} & 0 & 0\\ -\frac{1}{\tilde{\beta }} & \frac{\tilde{\alpha }}{{\tilde{\beta }}^{2}} & 0 & 0\\ 0 & 0 & \frac{1}{\tilde{v}} & 0\\ 0 & 0 & 0 & \frac{1}{2{\tilde{v}}^{2}} \end{array}\right).$$

Then, we have $\tilde{\nabla }KL\left(\tilde{\theta }\right)$ utilizing (1):

$$\begin{array}{cc} \tilde{\nabla }KL\left(\tilde{\theta }\right)= & \left(\begin{array}{cccc} \frac{\tilde{\alpha }}{\tilde{\alpha }\psi_{1}\left(\tilde{\alpha }\right)-1} & \frac{\tilde{\beta }}{\tilde{\alpha }\psi_{1}\left(\tilde{\alpha }\right)-1} & 0 & 0\\ \frac{\tilde{\beta }}{\tilde{\alpha }\psi_{1}\left(\tilde{\alpha }\right)-1} & \frac{{\tilde{\beta }}^{2}\psi_{1}\left(\tilde{\alpha }\right)}{\tilde{\alpha }\psi_{1}\left(\tilde{\alpha }\right)-1} & 0 & 0\\ 0 & 0 & \tilde{v} & 0\\ 0 & 0 & 0 & 2{\tilde{v}}^{2} \end{array}\right)\left(\begin{array}{c} \frac{\tilde{v}+{\left(\tilde{\mu }-{\tilde{\mu }}^{\prime }\right)}^{2}-\tilde{\beta }\psi_{1}\left(\tilde{\alpha }\right)}{2\tilde{\beta }}\\ \frac{-\tilde{\alpha }\left(\tilde{v}+{\left(\tilde{\mu }-{\tilde{\mu }}^{\prime }\right)}^{2}\right)+\tilde{\beta }}{2{\tilde{\beta }}^{2}}\\ \frac{\tilde{\alpha }\left({\tilde{\mu }}^{\prime }-\tilde{\mu }\right)}{\tilde{\beta }}\\ \frac{\tilde{\alpha }}{2\tilde{\beta }}-\frac{1}{2\tilde{v}} \end{array}\right)\\ = & \left(\begin{array}{c} -\frac{1}{2}\\ -\frac{1}{2}\left(\tilde{v}+{\left(\tilde{\mu }-{\tilde{\mu }}^{\prime }\right)}^{2}\right)\\ -\frac{\tilde{\alpha }\tilde{v}\left(\tilde{\mu }-{\tilde{\mu }}^{\prime }\right)}{\tilde{\beta }}\\ -\frac{\tilde{v}\left(\tilde{\beta }-\tilde{\alpha }\tilde{v}\right)}{\tilde{\beta }} \end{array}\right). \end{array}$$

$\tilde{\alpha }$, the first component of $\tilde{\nabla }KL\left(\tilde{\theta }\right)$, does not need an iteration and is fixed to $\tilde{\alpha }+\frac{1}{2}$, but others require iterations. Thus, the natural gradient algorithm based on (3) is as follows:

3. The natural gradient algorithm:

$$\begin{array}{ccc} {\tilde{\alpha }}^{\left(k+1\right)} & = & {\tilde{\alpha }}^{\left(k\right)},\\ {\tilde{\beta }}^{\left(k+1\right)} & = & {\tilde{\beta }}^{\left(k\right)}+\frac{1}{2}\left({\tilde{v}}^{\left(k\right)}+{\left(\tilde{\mu }-{\tilde{\mu }}^{\prime \left(k\right)}\right)}^{2}\right),\\ {\tilde{\mu }}^{\prime \left(k+1\right)} & = & {\tilde{\mu }}^{\prime \left(k\right)}+\frac{{\tilde{\alpha }}^{\left(k+1\right)}{\tilde{v}}^{\left(k\right)}\left(\tilde{\mu }-{\tilde{\mu }}^{\prime \left(k\right)}\right)}{{\tilde{\beta }}^{\left(k+1\right)}},\\ {\tilde{v}}^{\left(k+1\right)} & = & {\tilde{v}}^{\left(k\right)}+\frac{{\tilde{v}}^{\left(k\right)}\left({\tilde{\beta }}^{\left(k+1\right)}-{\tilde{\alpha }}^{\left(k+1\right)}{\tilde{v}}^{\left(k\right)}\right)}{{\tilde{\beta }}^{\left(k+1\right)}}. \end{array}$$

Another gradient algorithm to consider is based on the natural parameters $\tilde{\Lambda }$. We use $q(x,y|\tilde{\Lambda })$ instead of q(x,y) to clarify the computations, $\tilde{\Lambda }=\left({\tilde{\lambda }}_{1},{\tilde{\lambda }}_{2},{\tilde{\lambda }}_{3},{\tilde{\lambda }}_{4}\right)$:

$$q\left(x,y|\tilde{\Lambda }\right)\propto \exp\left[-{\tilde{\lambda }}_{4}\frac{x^{2}}{2}+{\tilde{\lambda }}_{3}x-{\tilde{\lambda }}_{1}\ln y-{\tilde{\lambda }}_{2}\frac{1}{y}\right],$$

where ${\tilde{\lambda }}_{1}=\tilde{\alpha }+1$, ${\tilde{\lambda }}_{2}=\tilde{\beta }$, ${\tilde{\lambda }}_{3}=\frac{{\tilde{\mu }}^{\prime }}{\tilde{v}}$, and ${\tilde{\lambda }}_{4}=\frac{1}{\tilde{v}}$. The logarithm of $q(x,y|\tilde{\Lambda })$ is:

$$\ln q\left(x,y|\tilde{\Lambda }\right)\propto -{\tilde{\lambda }}_{4}\frac{x^{2}}{2}+{\tilde{\lambda }}_{3}x-{\tilde{\lambda }}_{1}\ln y-{\tilde{\lambda }}_{2}\frac{1}{y}.$$

By substituting $\tilde{\Lambda }$ in (A5), we obtain:

$$\begin{array}{c} KL\left(\tilde{\Lambda }\right)\propto \frac{1}{2}\ln{\tilde{\lambda }}_{4}+\frac{1}{2}\left(\ln\;{\tilde{\lambda }}_{2}-\psi_{0}\left({\tilde{\lambda }}_{1}-1\right)\right)+\frac{\left({\tilde{\lambda }}_{1}-1\right)}{2{\tilde{\lambda }}_{2}}\left(\frac{1}{{\tilde{\lambda }}_{4}}+{\left(\tilde{\mu }-\frac{{\tilde{\lambda }}_{3}}{{\tilde{\lambda }}_{4}}\right)}^{2}\right). \end{array}$$

Then, we have:

(A8) $$\begin{array}{cc} \nabla KL(\tilde{\Lambda })= & \left(\frac{1}{2{\tilde{\lambda }}_{2}}\left(\frac{1}{{\tilde{\lambda }}_{4}}+{\left(\tilde{\mu }-\frac{{\tilde{\lambda }}_{3}}{{\tilde{\lambda }}_{4}}\right)}^{2}\right)-\frac{1}{2}\psi_{1}\left({\tilde{\lambda }}_{1}\right),\;-\frac{1}{2{\tilde{\lambda }}_{2}}-\frac{{\tilde{\lambda }}_{1}-1}{2{\tilde{\lambda }}_{2}^{2}}\left(\frac{1}{{\tilde{\lambda }}_{4}}+{\left(\tilde{\mu }-\frac{{\tilde{\lambda }}_{3}}{{\tilde{\lambda }}_{4}}\right)}^{2}\right),\right.\\ \; & \left.\;-\frac{{\tilde{\lambda }}_{1}-1}{{\tilde{\lambda }}_{2}{\tilde{\lambda }}_{4}}\left(\tilde{\mu }-\frac{{\tilde{\lambda }}_{3}}{{\tilde{\lambda }}_{4}}\right),\;\frac{1}{2{\tilde{\lambda }}_{4}}+\frac{{\tilde{\lambda }}_{1}-1}{2{\tilde{\lambda }}_{2}{\tilde{\lambda }}_{4}^{2}}\left(2{\tilde{\lambda }}_{3}\left(\tilde{\mu }-\frac{{\tilde{\lambda }}_{3}}{{\tilde{\lambda }}_{4}}\right)-1\right)\right). \end{array}$$

The $\nabla KL(\tilde{\Lambda })$ with respect to the natural parameters, in Equation (A8), is not equal to the one obtained from Equation (A6) after substituting the corresponding normal parameters as defined earlier. The issue pertains to the chain rule in the differentiation, which involves the relationship between ${\tilde{\lambda }}_{3}$ and ${\tilde{\lambda }}_{4}$. Thus, the new $\nabla KL(\tilde{\theta })$ is:

$$\begin{array}{cc} \nabla KL(\tilde{\theta })= & \left(\frac{\tilde{v}+{\left(\tilde{\mu }-{\tilde{\mu }}^{\prime }\right)}^{2}-\tilde{\beta }\psi_{1}\left(\tilde{\alpha }\right)}{2\tilde{\beta }},\;\frac{-\tilde{\alpha }\left(\tilde{v}+{\left(\tilde{\mu }-{\tilde{\mu }}^{\prime }\right)}^{2}\right)+\tilde{\beta }}{2{\tilde{\beta }}^{2}},\right.\\ \; & \;\left.\frac{\tilde{\alpha }\tilde{v}\left({\tilde{\mu }}^{\prime }-\tilde{\mu }\right)}{\tilde{\beta }},\;\frac{\tilde{v}}{2}-\frac{\tilde{\alpha }}{2\tilde{\beta }}\left({\tilde{v}}^{2}-2{\tilde{\mu }}^{\prime }\tilde{v}\left(\tilde{\mu }-{\tilde{\mu }}^{\prime }\right)\right)\right). \end{array}$$

4. The gradient-based algorithm concerning natural parameters:

$$\begin{array}{ccc} {\tilde{\alpha }}^{\left(k+1\right)} & = & {\tilde{\alpha }}^{\left(k\right)}-\left(\frac{{\tilde{v}}^{\left(k\right)}+{\left(\tilde{\mu }-{\tilde{\mu }}^{\prime \left(k\right)}\right)}^{2}-{\tilde{\beta }}^{\left(k\right)}\psi_{1}\left({\tilde{\alpha }}^{\left(k\right)}\right)}{2{\tilde{\beta }}^{\left(k\right)}}\right),\\ {\tilde{\beta }}^{\left(k+1\right)} & = & {\tilde{\beta }}^{\left(k\right)}-\left(\frac{-{\tilde{\alpha }}^{\left(k+1\right)}\left({\tilde{v}}^{\left(k\right)}+{\left(\tilde{\mu }-{\tilde{\mu }}^{\prime \left(k\right)}\right)}^{2}\right)+{\tilde{\beta }}^{\left(k\right)}}{2{\left[{\tilde{\beta }}^{\left(k\right)}\right]}^{2}}\right),\\ {\tilde{\mu }}^{\prime \left(k+1\right)} & = & {\tilde{\mu }}^{\prime \left(k\right)}-\left(\frac{{\tilde{\alpha }}^{\left(k+1\right)}{\tilde{v}}^{\left(k\right)}\left({\tilde{\mu }}^{\prime \left(k\right)}-\tilde{\mu }\right)}{{\tilde{\beta }}^{\left(k+1\right)}}\right),\\ {\tilde{v}}^{\left(k+1\right)} & = & {\tilde{v}}^{\left(k+1\right)}-\left(\frac{{\tilde{v}}^{\left(k\right)}}{2}-\frac{{\tilde{\alpha }}^{\left(k+1\right)}}{2{\tilde{\beta }}^{\left(k+1\right)}}\left({\left[{\tilde{v}}^{\left(k\right)}\right]}^{2}-2{\tilde{\mu }}^{\prime \left(k+1\right)}{\tilde{v}}^{\left(k\right)}\left(\tilde{\mu }-{\tilde{\mu }}^{\prime \left(k+1\right)}\right)\right)\right). \end{array}$$

## Appendix B. Conjugate Posterior of Normal-Inverse-Wishart of ($\tilde{\mu },\tilde{\Sigma }$)

The Normal-Inverse-Wishart distribution function $NIW(\tilde{\mu },\tilde{\Sigma }|{\tilde{\mu }}_{0},\tilde{\kappa },\tilde{\Psi },\tilde{\nu })$ is:

(A9) $$p\left(\tilde{\mu },\tilde{\Sigma }|{\tilde{\mu }}_{0},\tilde{\kappa },\tilde{\Psi },\tilde{\nu }\right)=N\left(\tilde{\mu }|{\tilde{\mu }}_{0},\frac{1}{\tilde{\kappa }}\tilde{\Sigma }\right)IW\left(\tilde{\Sigma }|\tilde{\Psi },\tilde{\nu }\right).$$

The explicit form of joint distribution $p(x,\tilde{\mu },\tilde{\Sigma })$ is:

(A10) $$\begin{array}{cc} p(x,\tilde{\mu },\tilde{\Sigma })= & p\left(x|\tilde{\mu },\tilde{\Sigma }\right)p\left(\tilde{\mu },\tilde{\Sigma }|{\tilde{\mu }}_{0},\tilde{\kappa },\tilde{\Psi },\tilde{\nu }\right)\\ = & \frac{1}{{\left(2\pi \right)}^{\frac{np}{2}}{∥\tilde{\Sigma }∥}^{\frac{n}{2}}}\exp\left\{-\frac{1}{2}\sum_{i=1}^{n}{\left(x_{i}-\tilde{\mu }\right)}^{⊺}{\tilde{\Sigma }}^{-1}\left(x_{i}-\tilde{\mu }\right)\right\}\\ \; & \frac{\sqrt{\tilde{\kappa }}}{{\left(2\pi \right)}^{\frac{p}{2}}{∥\tilde{\Sigma }∥}^{\frac{1}{2}}}\exp\left\{-\frac{\tilde{\kappa }}{2}\;{\left(\tilde{\mu }-{\tilde{\mu }}_{0}\right)}^{⊺}{\tilde{\Sigma }}^{-1}\left(\tilde{\mu }-{\tilde{\mu }}_{0}\right)\right\}\\ \; & \frac{∥\tilde{\Psi }{∥}^{\frac{\tilde{\nu }}{2}}}{2^{\frac{\tilde{\nu }p}{2}}\Gamma_{p}\left(\frac{\tilde{\nu }}{2}\right)}{∥\tilde{\Sigma }∥}^{-\frac{\tilde{\nu }+p+1}{2}}\exp\left\{-\frac{1}{2}\mathrm{Tr}\left[\tilde{\Psi }{\tilde{\Sigma }}^{-1}\right]\right\}. \end{array}$$

We rewrite the exponential expression of multivariate Normal Distribution as follows:

(A11) $$\sum_{i=1}^{n}{\left(x_{i}-\tilde{\mu }\right)}^{⊺}{\tilde{\Sigma }}^{-1}\left(x_{i}-\tilde{\mu }\right)=n{\left(\bar{x}-\tilde{\mu }\right)}^{⊺}{\tilde{\Sigma }}^{-1}\left(\bar{x}-\tilde{\mu }\right)+\sum_{i=1}^{n}{\left(x_{i}-\bar{x}\right)}^{⊺}{\tilde{\Sigma }}^{-1}\left(x_{i}-\bar{x}\right).$$

By substituting (A11) into (A10), we obtain:

(A12) $$\begin{array}{cc} p(x,\tilde{\mu },\tilde{\Sigma })\propto & ∥\tilde{\Sigma }{∥}^{-\frac{\tilde{\nu }+p+n+2}{2}}\exp\left\{-\frac{n}{2}{\left(\bar{x}-\tilde{\mu }\right)}^{⊺}{\tilde{\Sigma }}^{-1}\left(\bar{x}-\tilde{\mu }\right)-\frac{\tilde{\kappa }}{2}\;{\left(\tilde{\mu }-{\tilde{\mu }}_{0}\right)}^{⊺}{\tilde{\Sigma }}^{-1}\left(\tilde{\mu }-{\tilde{\mu }}_{0}\right)\right.\\ \; & \left.\;-\frac{1}{2}\mathrm{Tr}\left[\left(\tilde{\Psi }+\sum_{i=1}^{n}\left(x_{i}-\bar{x}\right){\left(x_{i}-\bar{x}\right)}^{⊺}\right){\tilde{\Sigma }}^{-1}\right]\right\}. \end{array}$$

We have to make integration over $\tilde{\Sigma }$ and put observations x instead of x to obtain the margin of $\tilde{\mu }$:

(A13) $$\begin{array}{cc} {\langle \ln p\left(\tilde{\mu },\tilde{\Sigma }\right)\rangle }_{q_{1}q_{3}}\propto & -\frac{n}{2}{\langle {\left(\bar{x}-\tilde{\mu }\right)}^{⊺}{\tilde{\Sigma }}^{-1}\left(\bar{x}-\tilde{\mu }\right)\rangle }_{q_{1}q_{3}}-\frac{\tilde{\kappa }}{2}\;{\langle {\left(\tilde{\mu }-{\tilde{\mu }}_{0}\right)}^{⊺}{\tilde{\Sigma }}^{-1}\left(\tilde{\mu }-{\tilde{\mu }}_{0}\right)\rangle }_{q_{1}q_{3}}\\ \propto & -\frac{\tilde{\kappa }+n}{2}{\left(\tilde{\mu }-\frac{\tilde{\kappa }{\tilde{\mu }}_{0}+n\bar{x}}{\tilde{\kappa }+n}\right)}^{⊺}{\tilde{\Lambda }}^{-1}\left(\tilde{\mu }-\frac{\tilde{\kappa }{\tilde{\mu }}_{0}+n\bar{x}}{\tilde{\kappa }+n}\right), \end{array}$$

where, ${\langle {\tilde{\Sigma }}^{-1}\rangle }_{q_{3}}={\tilde{\Lambda }}^{-1}$. Thus, $\tilde{\mu }$ has a Normal distribution $N\left(\frac{\tilde{\kappa }{\tilde{\mu }}_{0}+n\bar{x}}{\tilde{\kappa }+n},\frac{1}{\tilde{\kappa }+n}\tilde{\Lambda }\right)$. Also, we have the same for the margin of $\tilde{\Sigma }$:

(A14) $$\begin{array}{cc} {\langle \ln p\left(\tilde{\mu },\tilde{\Sigma }\right)\rangle }_{q_{1}q_{2}}\propto & -\frac{\tilde{\nu }+p+n+2}{2}\ln∥\tilde{\Sigma }∥-\frac{n}{2}{\langle {\left(\bar{x}-\tilde{\mu }\right)}^{⊺}{\tilde{\Sigma }}^{-1}\left(\bar{x}-\tilde{\mu }\right)\rangle }_{q_{1}q_{2}}\\ \; & -\frac{\tilde{\kappa }}{2}{\langle {\left(\tilde{\mu }-{\tilde{\mu }}_{0}\right)}^{⊺}{\tilde{\Sigma }}^{-1}\left(\tilde{\mu }-{\tilde{\mu }}_{0}\right)\rangle }_{q_{1}q_{2}}\\ \; & -\frac{1}{2}{\langle \mathrm{Tr}\left[\left(\tilde{\Psi }+\sum_{i=1}^{n}\left(x_{i}-\bar{x}\right){\left(x_{i}-\bar{x}\right)}^{⊺}\right){\tilde{\Sigma }}^{-1}\right]\rangle }_{q_{1}q_{2}}\\ \propto & -\frac{\tilde{\nu }+p+n+2}{2}\ln∥\tilde{\Sigma }∥-\frac{\tilde{\kappa }+n}{2}\langle {\left(\tilde{\mu }-\frac{\tilde{\kappa }{\tilde{\mu }}_{0}+n\bar{x}}{\tilde{\kappa }+n}\right)}^{⊺}{\tilde{\Sigma }}^{-1}\\ \; & \left(\tilde{\mu }-\frac{\tilde{\kappa }{\tilde{\mu }}_{0}+n\bar{x}}{\tilde{\kappa }+n}\right)\rangle_{q_{1}q_{2}}-\frac{1}{2}{\langle \mathrm{Tr}\left[\left(\tilde{\Psi }+\sum_{i=1}^{n}\left(x_{i}-\bar{x}\right){\left(x_{i}-\bar{x}\right)}^{⊺}\right){\tilde{\Sigma }}^{-1}\right]\rangle }_{q_{1}}\\ \propto & -\frac{\tilde{\nu }+p+n+2}{2}\ln∥\tilde{\Sigma }∥-\frac{1}{2}\mathrm{Tr}\left[\left(\left(\tilde{\kappa }+n\right)\langle \left(\tilde{\mu }-\frac{\tilde{\kappa }{\tilde{\mu }}_{0}+n\bar{x}}{\tilde{\kappa }+n}\right)\right.\right.\\ \; & \;\left.\left.{\left(\tilde{\mu }-\frac{\tilde{\kappa }{\tilde{\mu }}_{0}+n\bar{x}}{\tilde{\kappa }+n}\right)}^{⊺}\rangle_{q_{1}q_{2}}+\tilde{\Psi }+\sum_{i=1}^{n}\left(x_{i}-\bar{x}\right){\left(x_{i}-\bar{x}\right)}^{⊺}\right){\tilde{\Sigma }}^{-1}\right]\\ \propto & -\frac{\tilde{\nu }+p+n+2}{2}\ln∥\tilde{\Sigma }∥-\frac{1}{2}\mathrm{Tr}\left[\left(\tilde{\Lambda }+\tilde{\Psi }+\sum_{i=1}^{n}\left(x_{i}-\bar{x}\right){\left(x_{i}-\bar{x}\right)}^{⊺}\right){\tilde{\Sigma }}^{-1}\right]. \end{array}$$

So, $\tilde{\Sigma }$ has the structure of an Inverse Wishart distribution with the below parameters as following:

(A15) $$\tilde{\Sigma }∼IW\left(\tilde{\Lambda }+\tilde{\Psi }+\sum_{i=1}^{n}\left(x_{i}-\bar{x}\right){\left(x_{i}-\bar{x}\right)}^{⊺},\tilde{\nu }+n\right).$$

It is clear that x has again a multivariate Normal distribution whose mean and variance-covariance matrix are in the following expression

(A16) $$x∼MN\left(\frac{\tilde{\kappa }{\tilde{\mu }}_{0}+n\bar{x}}{\tilde{\kappa }+n},\frac{1}{\tilde{\nu }+n-p-1}\left[\tilde{\Lambda }+\tilde{\Psi }+\sum_{i=1}^{n}\left(x_{i}-\bar{x}\right){\left(x_{i}-\bar{x}\right)}^{⊺}\right]\right).$$

In the next step, we want to make an iterative algorithm of the above-mentioned marginal distributions. We need some pre-guesses for mean ${\tilde{\mu }}_{0}^{\left(1\right)}=\mu_{0}$, variance ${\tilde{\Lambda }}^{\left(1\right)}=\Lambda_{0}$, and precision ${\tilde{\kappa }}^{\left(1\right)}=\kappa_{0}$ of $\tilde{\mu }$ and also for scale matrix ${\tilde{\Psi }}^{\left(1\right)}=\Psi_{0}$ and degree of freedom ${\tilde{\nu }}^{\left(1\right)}=\nu_{0}+n$ of $\tilde{\Sigma }$. So, we can consider the standard alternative algorithm for Normal-Inverse-Wishart distribution as follows:

1. Standard alternate optimization algorithm:

$$\begin{array}{ccc} {\tilde{\kappa }}^{\left(k+1\right)} & = & {\tilde{\kappa }}^{\left(k\right)}+n,\\ {\tilde{\mu }}_{0}^{\left(k+1\right)} & = & \frac{{\tilde{\kappa }}^{\left(k+1\right)}{\tilde{\mu }}_{0}^{\left(k\right)}+n\bar{x}}{{\tilde{\kappa }}^{\left(k+1\right)}+n},\\ {\tilde{\Lambda }}^{\left(k+1\right)} & = & \frac{1}{{\tilde{\kappa }}^{\left(k+1\right)}+n}{\tilde{\Lambda }}^{\left(k\right)},\\ {\tilde{\nu }}^{\left(k+1\right)} & = & {\tilde{\nu }}^{\left(k\right)}+n,\\ {\tilde{\Psi }}^{\left(k+1\right)} & = & {\tilde{\Lambda }}^{\left(k+1\right)}+{\tilde{\Psi }}^{\left(k\right)}+\sum_{i=1}^{n}\left(x_{i}-\bar{x}\right){\left(x_{i}-\bar{x}\right)}^{⊺}. \end{array}$$

## Appendix C. KLD of Normal-Inverse-Wishart

First of all, we define $\tilde{\Theta }=\left(\tilde{\kappa },{\tilde{\mu }}_{0},\tilde{\Lambda },\tilde{\nu },\tilde{\Psi }\right)$ and the corresponding KLD function:

(A17) $$\begin{array}{cc} KL(\tilde{\Theta })= & \int \int q_{1}\left(\tilde{\mu }\right)q_{2}\left(\tilde{\Sigma }\right)\ln\left(\frac{q_{1}\left(\tilde{\mu }\right)q_{2}\left(\tilde{\Sigma }\right)}{p(\tilde{\mu },\tilde{\Sigma })}\right)d\tilde{\mu }d\tilde{\Sigma }\\ = & \int \int q_{1}\left(\tilde{\mu }\right)q_{2}\left(\tilde{\Sigma }\right)\ln\left(\frac{q_{2}\left(\tilde{\Sigma }\right)}{p(\tilde{\mu },\tilde{\Sigma })}\right)d\tilde{\mu }d\tilde{\Sigma }-H\left(q_{1}\right)\\ = & \int q_{2}\left(\tilde{\Sigma }\right)\left(\ln q_{2}\left(\tilde{\Sigma }\right)-\int q_{1}\left(\tilde{\mu }\right)\ln p\left(\tilde{\mu },\tilde{\Sigma }\right)d\tilde{\mu }\right)d\tilde{\Sigma }-H\left(q_{1}\right). \end{array}$$

We need to compute the internal integral first:

(A18) $$\begin{array}{cc} \int q_{1}\left(\tilde{\mu }\right)\ln p\left(\tilde{\mu },\tilde{\Sigma }\right)d\tilde{\mu }= & \int q_{1}\left(\tilde{\mu }\right)\ln\left\{\frac{\sqrt{\tilde{\kappa }}{∥\tilde{\Psi }∥}^{\frac{\tilde{\nu }}{2}}}{{\left(2\pi \right)}^{\frac{p}{2}}2^{\frac{\tilde{\nu }p}{2}}\Gamma_{p}\left(\frac{\tilde{\nu }}{2}\right)}\right.\\ \; & \;\left.∥\tilde{\Sigma }{∥}^{-\frac{\tilde{\nu }+p+2}{2}}\exp\left\{-\frac{\tilde{\kappa }}{2}\;{\left(\tilde{\mu }-{\tilde{\mu }}_{0}\right)}^{⊺}{\tilde{\Sigma }}^{-1}\left(\tilde{\mu }-{\tilde{\mu }}_{0}\right)-\frac{1}{2}\mathrm{Tr}\left[\tilde{\Psi }{\tilde{\Sigma }}^{-1}\right]\right\}\right\}d\tilde{\mu }\\ = & \ln\left\{\frac{\sqrt{\tilde{\kappa }}{∥\tilde{\Psi }∥}^{\frac{\tilde{\nu }}{2}}}{{\left(2\pi \right)}^{\frac{p}{2}}2^{\frac{\tilde{\nu }p}{2}}\Gamma_{p}\left(\frac{\tilde{\nu }}{2}\right)}{∥\tilde{\Sigma }∥}^{-\frac{\tilde{\nu }+p+2}{2}}\exp\left\{-\frac{1}{2}\mathrm{Tr}\left[\tilde{\Psi }{\tilde{\Sigma }}^{-1}\right]\right\}\right\}\\ \; & \;-\frac{\tilde{\kappa }}{2}\int q_{1}\left(\tilde{\mu }\right)\mathrm{Tr}\left[{\left(\tilde{\mu }-{\tilde{\mu }}_{0}\right)}^{⊺}{\tilde{\Sigma }}^{-1}\left(\tilde{\mu }-{\tilde{\mu }}_{0}\right)\right]d\tilde{\mu }\\ = & \ln\left\{\frac{\sqrt{\tilde{\kappa }}{∥\tilde{\Psi }∥}^{\frac{\tilde{\nu }}{2}}}{{\left(2\pi \right)}^{\frac{p}{2}}2^{\frac{\tilde{\nu }p}{2}}\Gamma_{p}\left(\frac{\tilde{\nu }}{2}\right)}{∥\tilde{\Sigma }∥}^{-\frac{\tilde{\nu }+p+2}{2}}\exp\left\{-\frac{1}{2}\mathrm{Tr}\left[\left(\tilde{\Lambda }+\tilde{\Psi }\right){\tilde{\Sigma }}^{-1}\right]\right\}\right\}. \end{array}$$

By substituting (A18) in side of (A17), we get:

(A19) $$\begin{array}{cc} KL(\tilde{\Theta })= & \int q_{2}\left(\tilde{\Sigma }\right)\left(\ln q_{2}\left(\tilde{\Sigma }\right)-\ln\left\{\frac{\sqrt{\tilde{\kappa }}{∥\tilde{\Psi }∥}^{\frac{\tilde{\nu }}{2}}}{{\left(2\pi \right)}^{\frac{p}{2}}2^{\frac{\tilde{\nu }p}{2}}\Gamma_{p}\left(\frac{\tilde{\nu }}{2}\right)}{∥\tilde{\Sigma }∥}^{-\frac{\tilde{\nu }+p+2}{2}}\right.\right.\\ \; & \left.\left.\exp\{-\frac{1}{2}\mathrm{Tr}\left[\left(\tilde{\Lambda }+\tilde{\Psi }\right){\tilde{\Sigma }}^{-1}\right]\}\right\}\right)d\tilde{\Sigma }-H\left(q_{1}\right). \end{array}$$

Since $q_{2}\left(\tilde{\Sigma }\right)=IW\left(\tilde{\Psi },\tilde{\nu }\right)$, we can rewrite $KL(\tilde{\Theta })$ as follows:

(A20) $$\begin{array}{cc} KL(\tilde{\Theta })= & -\ln\left\{\frac{\sqrt{\tilde{\kappa }}{∥\tilde{\Psi }∥}^{\frac{\tilde{\nu }}{2}}\Gamma_{p}\left(\frac{\tilde{\nu }+1}{2}\right)}{\pi^{\frac{p}{2}}\Gamma_{p}\left(\frac{\tilde{\nu }}{2}\right){∥\tilde{\Lambda }+\tilde{\Psi }∥}^{\frac{\tilde{\nu }+1}{2}}}\right\}\\ \; & +\int q_{2}\left(\tilde{\Sigma }\right)\ln\left(\frac{IW(\tilde{\Psi },\tilde{\nu })}{IW(\tilde{\Lambda }+\tilde{\Psi },\tilde{\nu }+1)}\right)d\tilde{\Sigma }-H\left(q_{1}\right). \end{array}$$

The second term is again a KLD function with respect to two Inverse Wishart distributions with different parameters calculated in [34]:

(A21) $$\begin{array}{cc} \int q_{2}\left(\tilde{\Sigma }\right) & \ln\left(\frac{IW(\tilde{\Psi },\tilde{\nu })}{IW(\tilde{\Lambda }+\tilde{\Psi },\tilde{\nu }+1)}\right)d\tilde{\Sigma }=\\ & \ln\left(\frac{\Gamma_{p}\left(\frac{\tilde{\nu }+1}{2}\right)}{\Gamma_{p}\left(\frac{\tilde{\nu }}{2}\right)}\right)+\frac{\tilde{\nu }}{2}\mathrm{Tr}\left[{\tilde{\Psi }}^{-1}\tilde{\Lambda }+I\right]-\frac{p\tilde{\nu }}{2}-\frac{\tilde{\nu }+1}{2}\ln∥{\tilde{\Psi }}^{-1}\tilde{\Lambda }+I∥-\frac{1}{2}\sum_{i=1}^{p}\psi_{0}\left(\frac{\tilde{\nu }-p+i}{2}\right). \end{array}$$

The last term of (A17) is the Shannon entropy of $\tilde{\mu }$:

(A22) $$\begin{array}{cc} -H(q_{1})= & \int q_{1}\left(\tilde{\mu }\right)\ln q_{1}\left(\tilde{\mu }\right)d\tilde{\mu }\\ = & \int q_{1}\left(\tilde{\mu }\right)\ln\left(\frac{{\tilde{\kappa }}^{\frac{p}{2}}}{{\left(2\pi \right)}^{\frac{p}{2}}{∥\tilde{\Lambda }∥}^{\frac{1}{2}}}\exp\left\{-\frac{\tilde{\kappa }}{2}{\left(\tilde{\mu }-{\tilde{\mu }}_{0}\right)}^{⊺}{\tilde{\Lambda }}^{-1}\left(\tilde{\mu }-{\tilde{\mu }}_{0}\right)\right\}\right)d\tilde{\mu }\\ = & \frac{p}{2}\ln\left(\tilde{\kappa }\right)-\frac{p}{2}\ln\left(2\pi \right)-\frac{1}{2}\ln∥\tilde{\Lambda }∥-\frac{\tilde{\kappa }}{2}\int q_{1}\left(\tilde{\mu }\right){\left(\tilde{\mu }-{\tilde{\mu }}_{0}\right)}^{⊺}{\tilde{\Lambda }}^{-1}\left(\tilde{\mu }-{\tilde{\mu }}_{0}\right)d\tilde{\mu }\\ = & \frac{p}{2}\ln\left(\tilde{\kappa }\right)-\frac{p}{2}\ln\left(2\pi \right)-\frac{1}{2}\ln∥\tilde{\Lambda }∥-\frac{p}{2}. \end{array}$$

The final expression of $KL(\tilde{\Theta })$ is equivalent to the following after substitution of (A21) and (A22) into (A20):

(A23) $$\begin{array}{cc} KL(\tilde{\Theta })= & -\frac{1}{2}\ln\tilde{\kappa }-\frac{\tilde{\nu }}{2}\ln∥\tilde{\Psi }∥+\frac{p}{2}\ln\pi -\ln\left(\frac{\Gamma_{p}\left(\frac{\tilde{\nu }+1}{2}\right)}{\Gamma_{p}\left(\frac{\tilde{\nu }}{2}\right)}\right)+\frac{\tilde{\nu }+1}{2}\ln∥\tilde{\Lambda }+\tilde{\Psi }∥\\ \; & +\ln\left(\frac{\Gamma_{p}\left(\frac{\tilde{\nu }+1}{2}\right)}{\Gamma_{p}\left(\frac{\tilde{\nu }}{2}\right)}\right)+\frac{\tilde{\nu }}{2}\mathrm{Tr}\left[{\tilde{\Psi }}^{-1}\tilde{\Lambda }+I\right]-\frac{p\tilde{\nu }}{2}-\frac{\tilde{\nu }+1}{2}\ln∥{\tilde{\Psi }}^{-1}\tilde{\Lambda }+I∥\\ \; & -\frac{1}{2}\sum_{i=1}^{p}\psi_{0}\left(\frac{\tilde{\nu }-p+i}{2}\right)+\frac{p}{2}\ln\left(\tilde{\kappa }\right)-\frac{p}{2}\ln\left(2\pi \right)-\frac{1}{2}\ln∥\tilde{\Lambda }∥-\frac{p}{2}\\ \propto & \frac{p-1}{2}\ln\tilde{\kappa }-\frac{\tilde{\nu }+1}{2}\ln\left(∥\tilde{\Psi }∥\cdot ∥{\tilde{\Psi }}^{-1}\tilde{\Lambda }+I∥\right)+\frac{1}{2}\ln∥\tilde{\Psi }∥+\frac{\tilde{\nu }+1}{2}\ln∥\tilde{\Lambda }+\tilde{\Psi }∥\\ \; & +\frac{\tilde{\nu }}{2}\mathrm{Tr}\left[{\tilde{\Psi }}^{-1}\tilde{\Lambda }+I\right]-\frac{p\tilde{\nu }}{2}-\frac{1}{2}\sum_{i=1}^{p}\psi_{0}\left(\frac{\tilde{\nu }-p+i}{2}\right)-\frac{1}{2}\ln∥\tilde{\Lambda }∥\\ \propto & \frac{p-1}{2}\ln\tilde{\kappa }+\frac{1}{2}\ln∥\tilde{\Psi }{\tilde{\Lambda }}^{-1}∥+\frac{\tilde{\nu }}{2}\mathrm{Tr}\left[{\tilde{\Psi }}^{-1}\tilde{\Lambda }+I\right]-\frac{p\tilde{\nu }}{2}-\frac{1}{2}\sum_{i=1}^{p}\psi_{0}\left(\frac{\tilde{\nu }-p+i}{2}\right). \end{array}$$

The gradient of $KL(\tilde{\Theta })$ is:

(A24) $$\begin{array}{cc} \nabla KL(\tilde{\Theta })= & \left(\frac{p-1}{2\tilde{\kappa }},\right.\;0,\;-\frac{1}{2}{\tilde{\Lambda }}^{-1}+\frac{\tilde{\nu }}{2}{\tilde{\Psi }}^{-1},\;\frac{1}{2}\mathrm{Tr}\left[{\tilde{\Psi }}^{-1}\tilde{\Lambda }+I\right]\\ \; & \;-\frac{p}{2}-\frac{1}{4}\sum_{i=1}^{p}\psi_{1}\left(\frac{\tilde{\nu }-p+i}{2}\right),\left.\;\frac{1}{2}{\tilde{\Psi }}^{-1}-\frac{\tilde{\nu }}{2}{\tilde{\Psi }}^{-1}\tilde{\Lambda }{\tilde{\Psi }}^{-1}\right), \end{array}$$

where 0 is the zero vector of *p* dimension. So, the parametric gradient and natural gradient algorithms are:

2. The gradient-based algorithm with normal parameters:

$$\begin{array}{ccc} {\tilde{\kappa }}^{\left(k+1\right)} & = & {\tilde{\kappa }}^{\left(k\right)}-\frac{\gamma }{2}\left(\frac{p-1}{{\tilde{\kappa }}^{\left(k\right)}}\right),\\ {\tilde{\mu }}_{0}^{\left(k+1\right)} & = & {\tilde{\mu }}_{0}^{\left(k\right)},\\ {\tilde{\Lambda }}^{\left(k+1\right)} & = & {\tilde{\Lambda }}^{\left(k\right)}-\frac{\gamma }{2}\left(-{\left[{\tilde{\Lambda }}^{\left(k\right)}\right]}^{-1}+{\tilde{\nu }}^{\left(k\right)}{\left[{\tilde{\Psi }}^{\left(k\right)}\right]}^{-1}\right),\\ {\tilde{\nu }}^{\left(k+1\right)} & = & {\tilde{\nu }}^{\left(k\right)}-\frac{\gamma }{2}\left(\mathrm{Tr}\left[{\left[{\tilde{\Psi }}^{\left(k\right)}\right]}^{-1}{\tilde{\Lambda }}^{\left(k+1\right)}+I\right]-p-\frac{1}{2}\sum_{i=1}^{p}\psi_{1}\left(\frac{{\tilde{\nu }}^{\left(k\right)}-p+i}{2}\right)\right),\\ {\tilde{\Psi }}^{\left(k+1\right)} & = & {\tilde{\Psi }}^{\left(k\right)}-\frac{\gamma }{2}\left({\left[{\tilde{\Psi }}^{\left(k\right)}\right]}^{-1}-{\tilde{\nu }}^{\left(k+1\right)}{\left[{\tilde{\Psi }}^{\left(k\right)}\right]}^{-1}{\tilde{\Lambda }}^{\left(k+1\right)}{\left[{\tilde{\Psi }}^{\left(k\right)}\right]}^{-1}\right). \end{array}$$

where γ is a fixed value for the gradient algorithm, and is proportional to 1 and 1/∥∇KL∥ for this algorithm. To get started with the natural gradient method, first, let us redefine $q(\tilde{\mu },\tilde{\Sigma })$ as $q(\tilde{\mu },\tilde{\Sigma }|\tilde{\Theta })$ and $\tilde{\Theta }$ as $\tilde{\Theta }=\left({\tilde{\mu }}_{0},\tilde{\Lambda },\tilde{\Psi }\right)$. We exclude $\tilde{\kappa }$ and $\tilde{\nu }$ from $\tilde{\Theta }$ and hold them constant because we require the estimated values of the remaining parameters. Then, we have:

$$\begin{array}{cc} q(\tilde{\mu },\tilde{\Sigma }|\tilde{\Theta })\propto & {\left(2\pi \right)}^{-\frac{p}{2}}{\tilde{\kappa }}^{\frac{1}{2}}{∥\tilde{\Lambda }∥}^{-\frac{1}{2}}\exp\left\{-\frac{\tilde{\kappa }}{2}{\left(\tilde{\mu }-{\tilde{\mu }}_{0}\right)}^{⊺}{\tilde{\Lambda }}^{-1}\left(\tilde{\mu }-{\tilde{\mu }}_{0}\right)\right\}\\ \; & \frac{∥\tilde{\Psi }{∥}^{\frac{\tilde{\nu }}{2}}}{{\left(2\pi \right)}^{\frac{\tilde{\nu }p}{2}}\Gamma \left(\frac{\tilde{\nu }}{2}\right)}{∥\tilde{\Sigma }∥}^{-\frac{\tilde{\nu }+p+1}{2}}\exp\left\{-\frac{1}{2}\mathrm{Tr}\left[\tilde{\Psi }{\tilde{\Sigma }}^{-1}\right]\right\}. \end{array}$$

Then, we need its logarithm function:

(A25) $$\begin{array}{cc} \ln q(\tilde{\mu },\tilde{\Sigma }|\tilde{\Theta })\propto & -\frac{1}{2}\ln∥\tilde{\Lambda }∥-\frac{\tilde{\kappa }}{2}{\left(\tilde{\mu }-{\tilde{\mu }}_{0}\right)}^{⊺}{\tilde{\Lambda }}^{-1}\left(\tilde{\mu }-{\tilde{\mu }}_{0}\right)\\ \; & +\frac{\tilde{\nu }}{2}\ln∥\tilde{\Psi }∥-\frac{\tilde{\nu }+p+1}{2}\ln∥\tilde{\Sigma }∥-\frac{1}{2}\mathrm{Tr}\left[\tilde{\Psi }{\tilde{\Sigma }}^{-1}\right]. \end{array}$$

The Fisher information is:

$$\begin{array}{cc} \bar{F}= & \langle \left(\begin{array}{cc} -\tilde{\kappa }{\tilde{\Lambda }}^{-1} & -\tilde{\kappa }{\left(\tilde{\mu }-{\tilde{\mu }}_{0}\right)}^{⊺}{\tilde{\Lambda }}^{-1}{\tilde{\Lambda }}^{-1}\\ -\tilde{\kappa }{\left(\tilde{\mu }-{\tilde{\mu }}_{0}\right)}^{⊺}{\tilde{\Lambda }}^{-1}{\tilde{\Lambda }}^{-1} & \frac{1}{2}{\tilde{\Lambda }}^{-1}{\tilde{\Lambda }}^{-1}-\tilde{\kappa }{\tilde{\Lambda }}^{-1}\left(\tilde{\mu }-{\tilde{\mu }}_{0}\right){\left(\tilde{\mu }-{\tilde{\mu }}_{0}\right)}^{⊺}{\tilde{\Lambda }}^{-1}{\tilde{\Lambda }}^{-1}\\ \left[0\right] & \left[0\right] \end{array}\right.\\ \; & \left.\begin{array}{c} \left[0\right]\\ \left[0\right]\\ -\frac{\tilde{\nu }}{2}{\tilde{\Psi }}^{-1}{\tilde{\Psi }}^{-1} \end{array}\right)\rangle_{q(\tilde{\mu },\tilde{\Sigma })}, \end{array}$$

where $\left[0\right]$ is a p×p matrix of 0. After taking into account the expectations of the Fisher matrix, we obtain the following:

$$\bar{F}=\left(\begin{array}{ccc} -\tilde{\kappa }{\tilde{\Lambda }}^{-1} & \left[0\right] & \left[0\right]\\ \left[0\right] & -\frac{1}{2}{\tilde{\Lambda }}^{-1}{\tilde{\Lambda }}^{-1} & \left[0\right]\\ \left[0\right] & \left[0\right] & -\frac{\tilde{\nu }}{2}{\tilde{\Psi }}^{-1}{\tilde{\Psi }}^{-1} \end{array}\right).$$

Then, we have $\tilde{\nabla }KL\left(\tilde{\Theta }\right)$, as well:

$$\begin{array}{cc} \tilde{\nabla }KL\left(\tilde{\Theta }\right)= & \left(\begin{array}{ccc} -\frac{1}{\tilde{\kappa }}\tilde{\Lambda } & \left[0\right] & \left[0\right]\\ \left[0\right] & -2{\tilde{\Lambda }}^{2} & \left[0\right]\\ \left[0\right] & \left[0\right] & -\frac{2}{\tilde{\nu }}{\tilde{\Psi }}^{2} \end{array}\right)\left(\begin{array}{c} \left[0\right]\\ -\frac{1}{2}{\tilde{\Lambda }}^{-1}+\frac{\tilde{\nu }}{2}{\tilde{\Psi }}^{-1}\\ \frac{1}{2}{\tilde{\Psi }}^{-1}-\frac{\tilde{\nu }}{2}{\tilde{\Psi }}^{-1}\tilde{\Lambda }{\tilde{\Psi }}^{-1} \end{array}\right)\\ = & \left(\begin{array}{c} \left[0\right]\\ \tilde{\Lambda }-\tilde{\nu }{\tilde{\Lambda }}^{2}{\tilde{\Psi }}^{-1}\\ -\frac{1}{\tilde{\nu }}\tilde{\Psi }+\tilde{\Psi }\tilde{\Lambda }{\tilde{\Psi }}^{-1} \end{array}\right). \end{array}$$

We replaced the zero vector $\left[0\right]$ with the zero matrix 0 in the first element of $\nabla KL(\tilde{\Theta })$ to feasible matrix multiplication. The natural gradient algorithm for the Normal-Inverse-Wishart example is as follows:

3. The natural gradient algorithm:

$$\begin{array}{ccc} {\tilde{\mu }}_{0}^{\left(k+1\right)} & = & {\tilde{\mu }}_{0}^{\left(k\right)},\\ {\tilde{\Lambda }}^{\left(k+1\right)} & = & \tilde{\nu }{\left[{\tilde{\Lambda }}^{\left(k\right)}\right]}^{2}{\left[{\tilde{\Psi }}^{\left(k\right)}\right]}^{-1},\\ {\tilde{\Psi }}^{\left(k+1\right)} & = & \frac{\tilde{\nu }+1}{\tilde{\nu }}{\tilde{\Psi }}^{\left(k\right)}-{\tilde{\Psi }}^{\left(k\right)}{\tilde{\Lambda }}^{\left(k+1\right)}{\left[{\tilde{\Psi }}^{\left(k\right)}\right]}^{-1}. \end{array}$$

In this algorithm, the iteration is just on $\tilde{\Lambda }$ and $\tilde{\Psi }$. We need to recall Equation (A25) for the final step of the gradient algorithm involving the natural parameters. We require the terms that depend on $\tilde{\mu }$ and $\tilde{\Sigma }$:

$$\begin{array}{c} \ln q\left(\tilde{\mu },\tilde{\Sigma }|\tilde{\Theta }\right)\propto \tilde{\kappa }{\tilde{\mu }}_{0}^{⊺}{\tilde{\Lambda }}^{-1}\tilde{\mu }-\frac{\tilde{\kappa }}{2}{\tilde{\mu }}^{⊺}{\tilde{\Lambda }}^{-1}\tilde{\mu }-\frac{\tilde{\nu }+p+1}{2}\ln∥\tilde{\Sigma }∥-\frac{1}{2}\mathrm{Tr}\left[\tilde{\Psi }{\tilde{\Sigma }}^{-1}\right]. \end{array}$$

We define the natural parameters as $L=({ł}_{1},{ł}_{2},{ł}_{3},{ł}_{4})$, where ${ł}_{1}=\tilde{\kappa }{\tilde{\mu }}_{0}^{⊺}{\tilde{\Lambda }}^{-1}$, ${ł}_{2}=\tilde{\kappa }{\tilde{\Lambda }}^{-1}$, ${ł}_{3}=\tilde{\nu }+p+1$, and ${ł}_{4}=\tilde{\Psi }$. Note that ${ł}_{1}$ is a *p*-vector, ${ł}_{3}$ is a scaler, and ${ł}_{2}$ and ${ł}_{4}$ are p×p matrices. Then, we have a new version of $\ln q(\tilde{\mu },\tilde{\Sigma }|\tilde{\Theta })$ represented as $\ln q(\tilde{\mu },\tilde{\Sigma }|L)$:

$$\begin{array}{c} \ln q\left(\tilde{\mu },\tilde{\Sigma }|L\right)\propto {ł}_{1}\tilde{\mu }-\frac{1}{2}{\tilde{\mu }}^{⊺}{ł}_{2}\tilde{\mu }-\frac{{ł}_{3}}{2}\ln∥\tilde{\Sigma }∥-\frac{1}{2}\mathrm{Tr}\left[{ł}_{4}{\tilde{\Sigma }}^{-1}\right]. \end{array}$$

We fix the $\tilde{\kappa }$ value and substitute ${\tilde{\mu }}_{0}={ł}_{1}{ł}_{2}^{-1}$, ${\tilde{\Lambda }}^{-1}=\frac{1}{\tilde{\kappa }}{ł}_{2}$ so $\tilde{\Lambda }=\tilde{\kappa }{ł}_{2}^{-1}$, $\tilde{\nu }={ł}_{3}-p-1$, and $\tilde{\Psi }={ł}_{4}$ inside Equation (A23) to obtain the new KL(L)

$$\begin{array}{cc} KL(L)\propto & \frac{p-n-1}{2}\ln\tilde{\kappa }+\frac{1}{2}\ln∥{ł}_{4}{ł}_{2}∥+\frac{{ł}_{3}-p-1}{2}\mathrm{Tr}\left[{ł}_{4}^{-1}{ł}_{2}^{-1}+I\right]\\ \; & -\frac{p({ł}_{3}-p-1)}{2}-\frac{1}{2}\sum_{i=1}^{p}\psi_{0}\left(\frac{{ł}_{3}-2p+i-1}{2}\right). \end{array}$$

Then, we have:

$$\begin{array}{cc} \nabla KL(L)= & \left(0,\;\frac{1}{2}{ł}_{2}^{-1}-\frac{{ł}_{3}-p-1}{2}{ł}_{2}^{-1}{ł}_{4}^{-1}{ł}_{2}^{-1},\;\frac{1}{2}\mathrm{Tr}\left[{ł}_{4}^{-1}{ł}_{2}^{-1}+I\right]-\frac{p}{2}\right.\\ \; & \left.\;-\frac{1}{4}\sum_{i=1}^{p}\psi_{1}\left(\frac{{ł}_{3}-2p+i-1}{2}\right),\;\frac{1}{2}{ł}_{4}^{-1}-\frac{{ł}_{3}-p-1}{2}{ł}_{4}^{-1}{ł}_{2}^{-1}{ł}_{4}^{-1}\right). \end{array}$$

To derive the gradient algorithm based on the natural parameters, we substitute the definition of L back into ∇KL(L) to obtain $\nabla KL(\tilde{\Theta })$:

$$\begin{array}{cc} \nabla KL(\tilde{\Theta })= & \left(0,\;\frac{1}{2\tilde{\kappa }}\tilde{\Lambda }-\frac{\tilde{\nu }}{2{\tilde{\kappa }}^{2}}\tilde{\Lambda }{\tilde{\Psi }}^{-1}\tilde{\Lambda },\;\frac{1}{2}\mathrm{Tr}\left[\frac{1}{\tilde{\kappa }}{\tilde{\Psi }}^{-1}\tilde{\Lambda }+I\right]-\frac{p}{2}-\frac{1}{4}\sum_{i=1}^{p}\psi_{1}\left(\frac{\tilde{\nu }-p+i}{2}\right),\right.\\ \; & \left.\;\frac{1}{2}{\tilde{\Psi }}^{-1}-\frac{\tilde{\nu }}{2\tilde{\kappa }}{\tilde{\Psi }}^{-1}\tilde{\Lambda }{\tilde{\Psi }}^{-1}\right). \end{array}$$

Comparing this gradient with Equation (A24), we notice that it has one less component, the first one is missed because we have fixed $\tilde{\kappa }$. Other elements of these two gradients are slightly different because ${ł}_{1}$ is a matrix multiple of ${ł}_{2}$. The last algorithm is:

4. The gradient-based algorithm concerning natural parameters:

$$\begin{array}{ccc} {\tilde{\mu }}_{0}^{\left(k+1\right)} & = & {\tilde{\mu }}^{\left(k\right)},\\ {\tilde{\Lambda }}^{\left(k+1\right)} & = & \frac{2\tilde{\kappa }-1}{2\tilde{\kappa }}{\tilde{\Lambda }}^{\left(k\right)}+\frac{{\tilde{\nu }}^{\left(k\right)}}{2{\tilde{\kappa }}^{2}}{\tilde{\Lambda }}^{\left(k\right)}{\left[{\tilde{\Psi }}^{\left(k\right)}\right]}^{-1}{\tilde{\Lambda }}^{\left(k\right)},\\ {\tilde{\nu }}^{\left(k+1\right)} & = & {\tilde{\nu }}^{\left(k\right)}-\frac{1}{2}\mathrm{Tr}\left[\frac{1}{\tilde{\kappa }}{\left[{\tilde{\Psi }}^{\left(k\right)}\right]}^{-1}{\tilde{\Lambda }}^{\left(k+1\right)}+I\right]+\frac{p}{2}+\frac{1}{4}\sum_{i=1}^{p}\psi_{1}\left(\frac{{\tilde{\nu }}^{\left(k\right)}-p+i}{2}\right),\\ {\tilde{\Psi }}^{\left(k+1\right)} & = & {\tilde{\Psi }}^{\left(k\right)}-\frac{1}{2}{\left[{\tilde{\Psi }}^{\left(k\right)}\right]}^{-1}+\frac{{\tilde{\nu }}^{\left(k+1\right)}}{2\tilde{\kappa }}{\left[{\tilde{\Psi }}^{\left(k\right)}\right]}^{-1}{\tilde{\Lambda }}^{\left(k+1\right)}{\left[{\tilde{\Psi }}^{\left(k\right)}\right]}^{-1}. \end{array}$$

## Appendix D. The Standard Alternative Optimization of the Linear Inverse Problem

In this example, the process is pretty much the same. First, we have to rewrite $p(g,\tilde{f},\tilde{v})$ and $\ln p(g,\tilde{f},\tilde{v})$:

(A26) $$\begin{array}{cc} p(g,\tilde{f},\tilde{v})= & \frac{1}{{\left(2\pi \right)}^{\frac{np}{2}}{∥\tilde{v_{\epsilon }}I∥}^{\frac{n}{2}}}\exp\left\{-\frac{1}{2}\sum_{i=1}^{n}{\left(g_{i}-\tilde{f}\right)}^{⊺}{\left(\tilde{v_{\epsilon }}I\right)}^{-1}\left(g_{i}-\tilde{f}\right)\right\}\frac{1}{{\left(2\pi \right)}^{\frac{p}{2}}{∥\mathrm{diag}\left[\tilde{v}\right]∥}^{\frac{1}{2}}}\\ \; & \exp\left\{-\frac{1}{2}{\tilde{f}}^{⊺}\mathrm{diag}{\left[\tilde{v}\right]}^{-1}\tilde{f}\right\}\prod_{j=1}^{p}\frac{{\tilde{\beta }}_{j}^{{\tilde{\alpha }}_{j}}}{\Gamma ({\tilde{\alpha }}_{j})}{\tilde{v}}_{j}^{-({\tilde{\alpha }}_{j}+1)}\exp\left\{-\frac{{\tilde{\beta }}_{j}}{{\tilde{v}}_{j}}\right\}, \end{array}$$

and

(A27) $$\begin{array}{cc} \ln p(g,\tilde{f},\tilde{v})\propto & -\frac{n}{2}\ln∥\tilde{v_{\epsilon }}I∥-\frac{1}{2}\sum_{i=1}^{n}{\left(g_{i}-\tilde{f}\right)}^{⊺}{\left(\tilde{v_{\epsilon }}I\right)}^{-1}\left(g_{i}-\tilde{f}\right)\\ \; & -\frac{1}{2}\ln∥\mathrm{diag}\left[\tilde{v}\right]∥-\frac{1}{2}{\tilde{f}}^{⊺}\mathrm{diag}{\left[\tilde{v}\right]}^{-1}\tilde{f}+\sum_{j=1}^{p}{\tilde{\alpha }}_{j}\ln{\tilde{\beta }}_{j}-\sum_{j=1}^{p}\ln\Gamma \left({\tilde{\alpha }}_{j}\right)\\ \; & -\sum_{j=1}^{p}\left({\tilde{\alpha }}_{j}+1\right)\ln{\tilde{v}}_{j}-\sum_{j=1}^{p}\frac{{\tilde{\beta }}_{j}}{{\tilde{v}}_{j}}, \end{array}$$

where, I is an identical matrix p×p. According to the above expression, it is easy to find out all margins functions starting from $\tilde{v}$:

$$\begin{array}{cc} {\langle \ln p\left(g,\tilde{f},\tilde{v}\right)\rangle }_{g,\tilde{f}}\propto & -\frac{1}{2}\ln∥\mathrm{diag}\left[\tilde{v}\right]∥-\frac{1}{2}{\langle {\tilde{f}}^{⊺}\mathrm{diag}{\left[\tilde{v}\right]}^{-1}\tilde{f}\rangle }_{\tilde{f}}-\sum_{j=1}^{p}\left({\tilde{\alpha }}_{j}+1\right)\ln{\tilde{v}}_{j}-\sum_{j=1}^{p}\frac{{\tilde{\beta }}_{j}}{{\tilde{v}}_{j}}\\ \propto & -\frac{1}{2}\sum_{j=1}^{p}\ln{\tilde{v}}_{i}-\frac{1}{2}\sum_{j=1}^{p}\frac{{\langle {\tilde{f}}_{k}^{2}\rangle }_{\tilde{f}}}{{\tilde{v}}_{i}}-\sum_{j=1}^{p}\left({\tilde{\alpha }}_{j}+1\right)\ln{\tilde{v}}_{j}-\sum_{j=1}^{p}\frac{{\tilde{\beta }}_{j}}{{\tilde{v}}_{j}}\\ \propto & -\left({\tilde{\alpha }}_{k}+\frac{3}{2}\right)\ln{\tilde{v}}_{k}-\left(\frac{{\tilde{v}}_{{\tilde{f}}_{k}}+{\tilde{\mu }}_{{\tilde{f}}_{k}}^{2}}{2}+{\tilde{\beta }}_{k}\right)\frac{1}{{\tilde{v}}_{k}}. \end{array}$$

Thus, ${\tilde{v}}_{k}∼IG\left({\tilde{\alpha }}_{k},\frac{{\tilde{v}}_{{\tilde{f}}_{k}}+{\tilde{\mu }}_{{\tilde{f}}_{k}}^{2}}{2}+{\tilde{\beta }}_{k}\right)$ for k=1,⋯,p. The process for computation of g density function is similar:

(A28) $$\begin{array}{cc} {\langle \ln p\left(g,\tilde{f},\tilde{v}\right)\rangle }_{\tilde{f},\tilde{v}}\propto & -\frac{1}{2}\sum_{i=1}^{n}\langle {\left(g_{i}-\tilde{f}\right)}^{⊺}{\left(\tilde{v_{\epsilon }}I\right)}^{-1}{(g_{i}-\tilde{f})\rangle }_{\tilde{f}}\\ = & -\frac{1}{2\tilde{v_{\epsilon }}}\sum_{i=1}^{n}\sum_{j=1}^{p}{\langle {\left(g_{ij}-{\tilde{f}}_{j}\right)}^{2}\rangle }_{\tilde{f}}\\ \propto & -\frac{1}{2\tilde{v_{\epsilon }}}\sum_{i=1}^{n}\sum_{j=1}^{p}{\left(g_{ij}-{\langle {\tilde{f}}_{j}\rangle }_{\tilde{f}}\right)}^{2}\\ = & -\frac{1}{2}\sum_{i=1}^{n}{\left(g_{i}-{\tilde{\mu }}_{\tilde{f}}\right)}^{⊺}{\left(\tilde{v_{\epsilon }}I\right)}^{-1}\left(g_{i}-{\tilde{\mu }}_{\tilde{f}}\right). \end{array}$$

So, g has again a Normal distribution of $N({\tilde{\mu }}_{\tilde{f}},\tilde{v_{\epsilon }}I)$. Since we need the density of $\bar{g}$ in the proceeding, we can specify it now. Therefore, back to properties of Normal distribution, we know that $\bar{g}$ has also a Normal density shown by $\bar{g}∼N\left({\tilde{\mu }}_{\tilde{f}},\frac{1}{n}\tilde{v_{\epsilon }}I\right)$. Now, we redo all calculation on (A29) for the objective margin of $\tilde{f}$, separately for each component denoted by ${\tilde{f}}_{k}$ for k=1,⋯,p:

(A29) $$\begin{array}{cc} {\langle \ln p\left(g,\tilde{f},\tilde{v}\right)\rangle }_{g,\tilde{v}}\propto & -\frac{1}{2}\sum_{i=1}^{n}{\langle {\left(g_{i}-\tilde{f}\right)}^{⊺}{\left(\tilde{v_{\epsilon }}I\right)}^{-1}\left(g_{i}-\tilde{f}\right)\rangle }_{g}-\frac{1}{2}{\langle {\tilde{f}}^{⊺}\mathrm{diag}{\left[\tilde{v}\right]}^{-1}\tilde{f}\rangle }_{\tilde{v}}\\ \propto & -\frac{1}{2}\left(n{\langle {\left(\bar{g}-\tilde{f}\right)}^{⊺}{\left(\tilde{v_{\epsilon }}I\right)}^{-1}\left(\bar{g}-\tilde{f}\right)\rangle }_{g}+{\langle {\tilde{f}}^{⊺}diag{\left[\tilde{v}\right]}^{-1}\tilde{f}\rangle }_{\tilde{v}}\right)\\ = & -\frac{1}{2}\left(\frac{n}{\tilde{v_{\epsilon }}}\sum_{j=1}^{p}{\langle {\left({\bar{g}}_{j}-{\tilde{f}}_{j}\right)}^{2}\rangle }_{g}+\sum_{j=1}^{p}{\langle \frac{1}{{\tilde{v}}_{j}}\rangle }_{\tilde{v}}{\tilde{f}}_{j}^{2}\right)\\ \propto & -\frac{1}{2}\sum_{j=1}^{p}\left(\frac{n}{\tilde{v_{\epsilon }}}+{\langle \frac{1}{{\tilde{v}}_{j}}\rangle }_{\tilde{v}}\right){\left({\tilde{f}}_{j}-\frac{{\langle {\bar{g}}_{j}\rangle }_{g}}{1+\frac{\tilde{v_{\epsilon }}}{n}{\langle \frac{1}{{\tilde{v}}_{j}}\rangle }_{\tilde{v}}}\right)}^{2}\\ = & -\frac{1}{2}\sum_{j=1}^{p}\left(\frac{n}{\tilde{v_{\epsilon }}}+\frac{2{\tilde{\alpha }}_{j}}{{\tilde{v}}_{{\tilde{f}}_{j}}+{\tilde{\mu }}_{{\tilde{f}}_{j}}^{2}+2{\tilde{\beta }}_{j}}\right){\left({\tilde{f}}_{j}-\frac{{\tilde{\mu }}_{g_{i}}}{1+\frac{2\tilde{v_{\epsilon }}{\tilde{\alpha }}_{j}}{n({\tilde{v}}_{{\tilde{f}}_{j}}+{\tilde{\mu }}_{{\tilde{f}}_{j}}^{2}+2{\tilde{\beta }}_{j})}}\right)}^{2}. \end{array}$$

Thus, ${\tilde{f}}_{k}$ has a Normal distribution with these structures:

(A30) $${\tilde{f}}_{k}∼N\left(\frac{{\tilde{\mu }}_{g_{k}}}{1+\frac{2\tilde{v_{\epsilon }}{\tilde{\alpha }}_{k}}{n({\tilde{v}}_{{\tilde{f}}_{k}}+{\tilde{\mu }}_{{\tilde{f}}_{k}}^{2}+2{\tilde{\beta }}_{k})}},\frac{\tilde{v_{\epsilon }}\left({\tilde{v}}_{{\tilde{f}}_{k}}+{\tilde{\mu }}_{{\tilde{f}}_{k}}^{2}+2{\tilde{\beta }}_{k}\right)}{n\left({\tilde{v}}_{{\tilde{f}}_{k}}+{\tilde{\mu }}_{{\tilde{f}}_{k}}^{2}\right)+2n{\tilde{\beta }}_{k}+2\tilde{v_{\epsilon }}{\tilde{\alpha }}_{k}}\right),\;k=1,\cdots ,p.$$

So, $\tilde{f}$ is separable due to the diag function. We summarize all in a multivariate Normal distribution and ${\tilde{\mu }}_{g}={\tilde{\mu }}_{\tilde{f}}$:

(A31) $$\tilde{f}∼MN\left(\frac{{\tilde{\mu }}_{\tilde{f}}}{1+\frac{2\tilde{v_{\epsilon }}\tilde{\alpha }}{n({\tilde{v}}_{\tilde{f}}+{\tilde{\mu }}_{\tilde{f}}^{2}+2\tilde{\beta })}},\mathrm{diag}\left[\frac{\tilde{v_{\epsilon }}\left({\tilde{v}}_{\tilde{f}}+{\tilde{\mu }}_{\tilde{f}}^{2}+2\tilde{\beta }\right)}{n\left({\tilde{v}}_{\tilde{f}}+{\tilde{\mu }}_{\tilde{f}}^{2}\right)+2n\tilde{\beta }+2\tilde{v_{\epsilon }}\tilde{\alpha }}\right]\right).$$

where all the mathematical operations on vectors are componentwise, and we find that ${\tilde{\mu }}_{g}={\tilde{\mu }}_{\tilde{f}}$. The recursive algorithm is fixed for $\tilde{v_{\epsilon }}$ and $\tilde{\alpha }$, which can be estimated from the data set. The alternative algorithm is in the following for other parameters:

1. Standard alternate optimization algorithm:

$$\begin{array}{ccc} {\tilde{v_{\epsilon }}}^{\left(k+1\right)} & = & {\tilde{v_{\epsilon }}}^{\left(k\right)},\\ {\tilde{\alpha }}^{\left(k+1\right)} & = & {\tilde{\alpha }}^{\left(k\right)},\\ {\tilde{\beta }}^{\left(k+1\right)} & = & \frac{1}{2}\left({\tilde{v}}_{\tilde{f}}^{\left(k\right)}+{\left[{\tilde{\mu }}_{\tilde{f}}^{\left(k\right)}\right]}^{2}\right)+{\tilde{\beta }}^{\left(k\right)},\\ {\tilde{\mu }}_{\tilde{f}}^{\left(k+1\right)} & = & {\tilde{\mu }}_{\tilde{f}}^{\left(k\right)}{\left(1+\frac{2{\tilde{v_{\epsilon }}}^{\left(k+1\right)}{\tilde{\alpha }}^{\left(k+1\right)}}{n({\tilde{v}}_{\tilde{f}}^{\left(k\right)}+{\left[{\tilde{\mu }}_{\tilde{f}}^{\left(k\right)}\right]}^{2}+2{\tilde{\beta }}^{\left(k+1\right)})}\right)}^{-1},\\ {\tilde{v}}_{\tilde{f}}^{\left(k+1\right)} & = & \mathrm{diag}\left[\frac{{\tilde{v_{\epsilon }}}^{\left(k+1\right)}\left({\tilde{v}}_{\tilde{f}}^{\left(k\right)}+{\left[{\tilde{\mu }}_{\tilde{f}}^{\left(k+1\right)}\right]}^{2}+2{\tilde{\beta }}^{\left(k+1\right)}\right)}{n\left({\tilde{v}}_{\tilde{f}}^{\left(k\right)}+{\left[{\tilde{\mu }}_{\tilde{f}}^{\left(k+1\right)}\right]}^{2}\right)+2n{\tilde{\beta }}^{\left(k+1\right)}+2{\tilde{v_{\epsilon }}}^{\left(k+1\right)}{\tilde{\alpha }}^{\left(k+1\right)}}\right]. \end{array}$$

## Appendix E. *KL*($\tilde{\theta }$) of the Linear Inverse Problem

We define $\tilde{\theta }=\left({\tilde{\mu }}_{g},\tilde{v_{\epsilon }},{\tilde{v}}_{\tilde{f}},\tilde{\alpha },\tilde{\beta }\right)$ and its corresponding $KL(\tilde{\theta })$:

(A32) $$KL\left(\tilde{\theta }\right)=-H\left(q_{1}\right)-H\left(q_{2}\right)-H\left(q_{3}\right)-{\langle \ln p\left(g,\tilde{f},\tilde{v}\right)\rangle }_{q_{1}q_{2}q_{3}}.$$

We separately decompose each term initializing from $-H(q_{1})$, the negative entropy of g:

(A33) $$\begin{array}{cc} -H(q_{1})= & \int q_{1}\left(g\right)\ln\left(\frac{1}{{\left(2\pi \right)}^{\frac{pn}{2}}{∥\mathrm{diag}\left[\tilde{v_{\epsilon }}I\right]∥}^{\frac{n}{2}}}\right.\\ \; & \left.\;\exp\{-\frac{1}{2}\sum_{i=1}^{n}{\left(g_{i}-{\tilde{\mu }}_{g}\right)}^{⊺}\mathrm{diag}{\left[\tilde{v_{\epsilon }}\right]}^{-1}\left(g_{i}-{\tilde{\mu }}_{g}\right)\}\right)dg\\ = & -\frac{pn}{2}-\frac{pn}{2}\ln\left(2\pi \right)-\frac{pn}{2}\ln\tilde{v_{\epsilon }}, \end{array}$$

and the negative entropy of $\tilde{f}$:

(A34) $$\begin{array}{cc} -H(q_{2})= & \int q_{2}\left(\tilde{f}\right)\ln\left(\frac{1}{{\left(2\pi \right)}^{\frac{p}{2}}{∥\mathrm{diag}\left[{\tilde{v}}_{\tilde{f}}\right]∥}^{\frac{1}{2}}}\exp\left\{-\frac{1}{2}{\tilde{f}}^{⊺}\mathrm{diag}{\left[{\tilde{v}}_{\tilde{f}}\right]}^{-1}\tilde{f}\right\}\right)d\tilde{f}\\ = & -\frac{p}{2}-\frac{p}{2}\ln\left(2\pi \right)-\frac{1}{2}\ln∥\mathrm{diag}\left[{\tilde{v}}_{\tilde{f}}\right]∥, \end{array}$$

and the negative entropy of $\tilde{v}$:

(A35) $$\begin{array}{cc} -H(q_{3})= & \int q_{3}\left(\tilde{v}\right)\ln\left(\prod_{j=1}^{p}\frac{{\tilde{\beta }}_{j}^{{\tilde{\alpha }}_{j}}}{\Gamma ({\tilde{\alpha }}_{j})}{\tilde{v}}_{j}^{-({\tilde{\alpha }}_{j}+1)}\exp\left\{-\frac{{\tilde{\beta }}_{j}}{{\tilde{v}}_{j}}\right\}\right)d\tilde{v}\\ = & -\sum_{j=1}^{p}{\tilde{\alpha }}_{j}-\sum_{j=1}^{p}\ln\left({\tilde{\beta }}_{j}\Gamma \left({\tilde{\alpha }}_{j}\right)\right)+\sum_{j=1}^{p}\left({\tilde{\alpha }}_{j}+1\right)\psi_{0}\left({\tilde{\alpha }}_{j}\right), \end{array}$$

and the last term of $KL(\tilde{\theta })$ according to Equation (A27):

(A36) $$\begin{array}{cc} -{\langle \ln p\left(g,\tilde{f},\tilde{v}\right)\rangle }_{q_{1}q_{2}q_{3}}\propto & \int \int \int q_{1}\left(g\right)q_{2}\left(\tilde{f}\right)q_{3}\left(\tilde{v}\right)\left(\frac{n}{2}\ln∥\tilde{v_{\epsilon }}I∥\right.\\ \; & +\frac{1}{2}\sum_{i=1}^{n}{\left(g_{i}-\tilde{f}\right)}^{⊺}{\left(\tilde{v_{\epsilon }}I\right)}^{-1}\left(g_{i}-\tilde{f}\right)+\frac{1}{2}\ln∥\mathrm{diag}\left[\tilde{v}\right]∥\\ \; & +\frac{1}{2}{\tilde{f}}^{⊺}\mathrm{diag}{\left[\tilde{v}\right]}^{-1}\tilde{f}-\sum_{j=1}^{p}{\tilde{\alpha }}_{j}\ln{\tilde{\beta }}_{j}+\sum_{j=1}^{p}\ln\Gamma \left({\tilde{\alpha }}_{j}\right)\\ \; & \left.+\sum_{j=1}^{p}\left({\tilde{\alpha }}_{j}+1\right)\ln{\tilde{v}}_{j}+\sum_{j=1}^{p}\frac{{\tilde{\beta }}_{j}}{{\tilde{v}}_{j}}\right)dgd\tilde{f}d\tilde{v}\\ \propto & \frac{np}{2}\ln\tilde{v_{\epsilon }}+\frac{1}{2\tilde{v_{\epsilon }}}\sum_{i=1}^{n}\sum_{j=1}^{p}\int \int q_{1}\left(g\right)q_{2}\left(\tilde{f}\right)\left(g_{ij}^{2}+{\tilde{f}}_{j}^{2}\right)dgd\tilde{f}\\ \; & +\frac{1}{2}\sum_{j=1}^{p}\int \int \frac{{\tilde{f}}_{j}^{2}}{{\tilde{v}}_{j}}d\tilde{f}d\tilde{v}-\sum_{j=1}^{p}{\tilde{\alpha }}_{j}\ln{\tilde{\beta }}_{j}+\sum_{j=1}^{p}\ln\Gamma \left({\tilde{\alpha }}_{j}\right)\\ \; & +\int q_{3}\left(\tilde{v}\right)\left(\sum_{j=1}^{p}\left({\tilde{\alpha }}_{j}+\frac{3}{2}\right)\ln{\tilde{v}}_{j}+\sum_{j=1}^{p}\frac{{\tilde{\beta }}_{j}}{{\tilde{v}}_{j}}\right)d\tilde{v}\\ \propto & \frac{np}{2}\ln\tilde{v_{\epsilon }}+\frac{n}{2\tilde{v_{\epsilon }}}\sum_{j=1}^{p}\left(\tilde{v_{\epsilon }}+{\tilde{\mu }}_{g_{j}}^{2}+{\tilde{v}}_{{\tilde{f}}_{j}}\right)+\sum_{j=1}^{p}\frac{{\tilde{\alpha }}_{j}{\tilde{v}}_{{\tilde{f}}_{j}}}{{\tilde{\beta }}_{j}}-\sum_{j=1}^{p}{\tilde{\alpha }}_{j}\ln{\tilde{\beta }}_{j}\\ \; & +\sum_{j=1}^{p}\ln\Gamma \left({\tilde{\alpha }}_{j}\right)+\sum_{j=1}^{p}\left({\tilde{\alpha }}_{j}+\frac{3}{2}\right)\left(\ln{\tilde{\beta }}_{j}-\psi_{0}\left({\tilde{\alpha }}_{j}\right)\right)+\sum_{j=1}^{p}{\tilde{\alpha }}_{j}. \end{array}$$

Finally, we get:

$$\begin{array}{cc} KL(\tilde{\theta })\propto & -\frac{pn}{2}\ln\tilde{v_{\epsilon }}-\frac{1}{2}\ln∥\mathrm{diag}\left[{\tilde{v}}_{\tilde{f}}\right]∥-\sum_{j=1}^{p}\ln\left({\tilde{\beta }}_{j}\Gamma \left({\tilde{\alpha }}_{j}\right)\right)+\sum_{j=1}^{p}\left({\tilde{\alpha }}_{j}+1\right)\psi_{0}\left({\tilde{\alpha }}_{j}\right)\\ \; & +\frac{np}{2}\ln\tilde{v_{\epsilon }}+\frac{n}{2\tilde{v_{\epsilon }}}\sum_{j=1}^{p}\left(\tilde{v_{\epsilon }}+{\tilde{\mu }}_{g_{j}}^{2}+{\tilde{v}}_{{\tilde{f}}_{j}}\right)+\sum_{j=1}^{p}\frac{{\tilde{\alpha }}_{j}{\tilde{v}}_{{\tilde{f}}_{j}}}{{\tilde{\beta }}_{j}}-\sum_{j=1}^{p}{\tilde{\alpha }}_{j}\ln{\tilde{\beta }}_{j}\\ \; & +\sum_{j=1}^{p}\ln\Gamma \left({\tilde{\alpha }}_{j}\right)+\sum_{j=1}^{p}\left({\tilde{\alpha }}_{j}+\frac{3}{2}\right)\left(\ln{\tilde{\beta }}_{j}-\psi_{0}\left({\tilde{\alpha }}_{j}\right)\right).\\ \propto & -\frac{1}{2}\sum_{j=1}^{p}\ln{\tilde{v}}_{{\tilde{f}}_{j}}+\frac{n}{2\tilde{v_{\epsilon }}}\sum_{j=1}^{p}\left({\tilde{\mu }}_{g_{j}}^{2}+{\tilde{v}}_{{\tilde{f}}_{j}}\right)+\sum_{j=1}^{p}\frac{{\tilde{\alpha }}_{j}{\tilde{v}}_{{\tilde{f}}_{j}}}{{\tilde{\beta }}_{j}}-\frac{1}{2}\sum_{j=1}^{p}\psi_{0}\left({\tilde{\alpha }}_{j}\right)+\frac{1}{2}\sum_{j=1}^{p}\ln{\tilde{\beta }}_{j}. \end{array}$$

To make the last two algorithms, we have to differentiate $KL(\tilde{\theta })$ with respect to $\tilde{\theta }$:

$$\begin{array}{cc} \nabla KL(\tilde{\theta })= & \left(\frac{n}{\tilde{v_{\epsilon }}}{\tilde{\mu }}_{g},\;-\frac{n}{2{\tilde{v_{\epsilon }}}^{2}}\sum_{j=1}^{p}\left({\tilde{\mu }}_{g_{j}}^{2}+{\tilde{v}}_{{\tilde{f}}_{j}}\right),\;\frac{n}{2\tilde{v_{\epsilon }}}1-\frac{1}{2}{\tilde{v}}_{\tilde{f}}^{-1}+\frac{\tilde{\alpha }}{\tilde{\beta }},\right.\\ \; & \left.\;-\frac{1}{2}\psi_{1}\left(\tilde{\alpha }\right)+\frac{{\tilde{v}}_{\tilde{f}}}{\tilde{\beta }},\;\frac{1}{2}{\tilde{\beta }}^{-1}-\frac{\tilde{\alpha }{\tilde{v}}_{\tilde{f}}}{{\tilde{\beta }}^{2}}\right), \end{array}$$

where 1 is the all-ones vector, and also all operations are componentwise. The gradient algorithm is as follows:

**2. The gradient-based algorithm with normal parameters:**

$$\begin{array}{ccc} {\tilde{\mu }}_{g}^{\left(k+1\right)} & = & {\tilde{\mu }}_{g}^{\left(k\right)}-\gamma \left(\frac{n}{{\tilde{v_{\epsilon }}}^{\left(k\right)}}{\tilde{\mu }}_{g}^{\left(k\right)}\right),\\ {\tilde{v_{\epsilon }}}^{\left(k+1\right)} & = & {\tilde{v_{\epsilon }}}^{\left(k\right)}+\gamma \left(\frac{n}{2{\left[{\tilde{v_{\epsilon }}}^{\left(k\right)}\right]}^{2}}\sum_{j=1}^{p}\left(2{\tilde{v}}_{{\tilde{f}}_{j}}^{\left(k\right)}+{\left[{\tilde{\mu }}_{g_{j}}^{\left(k+1\right)}\right]}^{2}\right)\right),\\ {\tilde{v}}_{\tilde{f}}^{\left(k+1\right)} & = & {\tilde{v}}_{\tilde{f}}^{\left(k\right)}-\gamma \left(\frac{n}{{\tilde{v_{\epsilon }}}^{\left(k+1\right)}}1-\frac{1}{2}{\left[{\tilde{v}}_{\tilde{f}}^{\left(k\right)}\right]}^{-1}+\frac{{\tilde{\alpha }}^{\left(k\right)}}{{\tilde{\beta }}^{\left(k\right)}}\right),\\ {\tilde{\alpha }}^{\left(k+1\right)} & = & {\tilde{\alpha }}^{\left(k\right)}-\gamma \left(\frac{{\tilde{v}}_{\tilde{f}}^{\left(k+1\right)}}{{\tilde{\beta }}^{\left(k\right)}}-\frac{1}{2}\psi_{1}\left({\tilde{\alpha }}^{\left(k\right)}\right)\right),\\ {\tilde{\beta }}^{\left(k+1\right)} & = & {\tilde{\beta }}^{\left(k\right)}-\gamma \left(\frac{1}{2}{\left[{\tilde{\beta }}^{\left(k\right)}\right]}^{-1}-\frac{{\tilde{\alpha }}^{\left(k+1\right)}{\tilde{v}}_{\tilde{f}}^{\left(k+1\right)}}{{\left[{\tilde{\beta }}^{\left(k\right)}\right]}^{2}}\right). \end{array}$$

Another algorithm we consider here is the natural gradient algorithm with respect to the redefine $\tilde{\theta }$. We fix $\tilde{v_{\epsilon }}$ and determine $\tilde{\theta }=\left({\tilde{\mu }}_{g},{\tilde{v}}_{\tilde{f}},\tilde{\alpha },\tilde{\beta }\right)$ because the empirical Fisher information respect to $\tilde{v_{\epsilon }}$ is 0. It is needed to calculate the Fisher information of $\ln q(g,\tilde{f},\tilde{v}|\tilde{\theta })$:

$$\begin{array}{cc} \ln q(g,\tilde{f},\tilde{v}|\tilde{\theta })\propto & -\frac{pn}{2}\ln\tilde{v_{\epsilon }}-\frac{1}{2\tilde{v_{\epsilon }}}\sum_{i=1}^{n}\sum_{j=1}^{p}{\left(g_{ij}-{\tilde{\mu }}_{g_{j}}\right)}^{2}-\frac{1}{2}\sum_{j=1}^{p}\ln{\tilde{v}}_{{\tilde{f}}_{j}}\\ \; & -\frac{1}{2}{\tilde{f}}^{⊺}\mathrm{diag}{\left[{\tilde{v}}_{\tilde{f}}\right]}^{-1}\tilde{f}+\sum_{j=1}^{p}{\tilde{\alpha }}_{j}\ln{\tilde{\beta }}_{j}-\sum_{j=1}^{p}\ln\Gamma \left({\tilde{\alpha }}_{j}\right)-\sum_{j=1}^{p}\left({\tilde{\alpha }}_{j}+1\right)\ln{\tilde{v}}_{j}-\sum_{j=1}^{p}\frac{{\tilde{\beta }}_{j}}{{\tilde{v}}_{j}}. \end{array}$$

Its corresponding Fisher information matrix is:

$$\begin{array}{cc} \bar{F}= & \langle \left(\begin{array}{cccc} -\frac{n}{\tilde{v_{\epsilon }}}1 & 0 & 0 & 0\\ 0 & \frac{1}{2{\tilde{v}}_{\tilde{f}}^{2}}-\frac{{\tilde{f}}^{⊺}\tilde{f}}{{\tilde{v}}_{\tilde{f}}^{3}} & 0 & 0\\ 0 & 0 & -\Psi_{1}\left(\tilde{\alpha }\right) & \frac{1}{\tilde{\beta }}\\ 0 & 0 & \frac{1}{\tilde{\beta }} & -\frac{\tilde{\alpha }}{{\tilde{\beta }}^{2}} \end{array}\right)\rangle_{q(g,\tilde{f},\tilde{v})}\\ = & \left(\begin{array}{cccc} -\frac{n}{\tilde{v_{\epsilon }}}1 & 0 & 0 & 0\\ 0 & -\frac{1}{2{\tilde{v}}_{\tilde{f}}^{2}} & 0 & 0\\ 0 & 0 & -\Psi_{1}\left(\tilde{\alpha }\right) & \frac{1}{\tilde{\beta }}\\ 0 & 0 & \frac{1}{\tilde{\beta }} & -\frac{\tilde{\alpha }}{{\tilde{\beta }}^{2}} \end{array}\right). \end{array}$$

Then, we can calculate $\tilde{\nabla }KL\left(\tilde{\theta }\right)$ as follows:

$$\tilde{\nabla }KL\left(\tilde{\theta }\right)=\left(\begin{array}{cccc} -\frac{\tilde{v_{\epsilon }}}{n}1 & 0 & 0 & 0\\ 0 & -2{\tilde{v}}_{\tilde{f}}^{2} & 0 & 0\\ 0 & 0 & \frac{-\tilde{\alpha }}{\tilde{\alpha }\Psi_{1}\left(\tilde{\alpha }\right)-1} & \frac{-\tilde{\beta }}{\tilde{\alpha }\Psi_{1}\left(\tilde{\alpha }\right)-1}\\ 0 & 0 & \frac{-\tilde{\beta }}{\tilde{\alpha }\Psi_{1}\left(\tilde{\alpha }\right)-1} & \frac{-{\tilde{\beta }}^{2}\Psi_{1}\left(\tilde{\alpha }\right)}{\tilde{\alpha }\Psi_{1}\left(\tilde{\alpha }\right)-1} \end{array}\right)\left(\begin{array}{c} \frac{n}{\tilde{v_{\epsilon }}}{\tilde{\mu }}_{g}\\ \frac{n}{2\tilde{v_{\epsilon }}}1-\frac{1}{2}{\tilde{v}}_{\tilde{f}}^{-1}+\frac{\tilde{\alpha }}{\tilde{\beta }}\\ -\frac{1}{2}\psi_{1}\left(\tilde{\alpha }\right)+\frac{{\tilde{v}}_{\tilde{f}}}{\tilde{\beta }}\\ \frac{1}{2}{\tilde{\beta }}^{-1}-\frac{\tilde{\alpha }{\tilde{v}}_{\tilde{f}}}{{\tilde{\beta }}^{2}} \end{array}\right)=\left(\begin{array}{c} -{\tilde{\mu }}_{g}\\ 2{\tilde{v}}_{\tilde{f}}^{2}\left(\frac{-n}{2\tilde{v_{\epsilon }}}1+\frac{1}{2}{\tilde{v}}_{\tilde{f}}^{-1}-\frac{\tilde{\alpha }}{\tilde{\beta }}\right)\\ \frac{1}{2}\\ \frac{-\tilde{\beta }\Psi_{1}\left(\tilde{\alpha }\right)}{2(\tilde{\alpha }\Psi_{1}\left(\tilde{\alpha }\right)-1)}+{\tilde{v}}_{\tilde{f}} \end{array}\right).$$

For the natural gradient algorithm, we set ${\tilde{\mu }}_{g}^{\left(k+1\right)}=2{\tilde{\mu }}_{g}$ and ${\tilde{\alpha }}^{\left(k+1\right)}=\tilde{\alpha }-\frac{1}{2}$, and repeat the algorithm for ${\tilde{v}}_{\tilde{f}}$ and $\tilde{\beta }$.

3. The natural gradient algorithm:

$$\begin{array}{ccc} {\tilde{v}}_{\tilde{f}}^{\left(k+1\right)} & = & {\tilde{v}}_{\tilde{f}}^{\left(k\right)}-2{\left[{\tilde{v}}_{\tilde{f}}^{\left(k\right)}\right]}^{2}\left(\frac{-n}{2\tilde{v_{\epsilon }}}1+\frac{1}{2}{\left[{\tilde{v}}_{\tilde{f}}^{\left(k\right)}\right]}^{-1}-\frac{\tilde{\alpha }}{{\tilde{\beta }}^{\left(k\right)}}\right),\\ {\tilde{\beta }}^{\left(k+1\right)} & = & {\tilde{\beta }}^{\left(k\right)}\left(1+\frac{\Psi_{1}\left(\tilde{\alpha }\right)}{2(\tilde{\alpha }\Psi_{1}\left(\tilde{\alpha }\right)-1)}\right)+{\tilde{v}}_{\tilde{f}}^{\left(k+1\right)}. \end{array}$$

For the last algorithm, we rewrite $\ln q(g,\tilde{f},\tilde{v}|\tilde{\Lambda })$ respect to the natural parameters:

$$\begin{array}{cc} \ln q(g,\tilde{f},\tilde{v}|\tilde{\Lambda })\propto & \frac{{\tilde{\lambda }}_{1}}{2}\sum_{i=1}^{n}g_{i}-\frac{{\tilde{\lambda }}_{2}}{2}\sum_{i=1}^{n}g_{i}^{⊺}g_{i}-\frac{1}{2}{\tilde{f}}^{⊺}{\tilde{\lambda }}_{3}\tilde{f}-\sum_{j=1}^{p}{\tilde{\lambda }}_{4j}\ln{\tilde{v}}_{j}-\sum_{j=1}^{p}\frac{{\tilde{\lambda }}_{5j}}{{\tilde{v}}_{j}}, \end{array}$$

where $\tilde{\Lambda }$ is defined with $\left({\tilde{\lambda }}_{1},{\tilde{\lambda }}_{2},{\tilde{\lambda }}_{3},{\tilde{\lambda }}_{4},{\tilde{\lambda }}_{5}\right)$ such that ${\tilde{\lambda }}_{1}=\frac{{\tilde{\mu }}_{g}^{⊺}}{\tilde{v_{\epsilon }}}$, ${\tilde{\lambda }}_{2}=\frac{1}{\tilde{v_{\epsilon }}}$, ${\tilde{\lambda }}_{3}=\frac{1}{{\tilde{v}}_{\tilde{f}}}$, ${\tilde{\lambda }}_{4}=\tilde{\alpha }+1$, ${\tilde{\lambda }}_{5}=\tilde{\beta }$. Then, the corresponding $KL(\tilde{\Lambda })$ is:

$$\begin{array}{cc} KL(\tilde{\Lambda })\propto & \frac{1}{2}\sum_{j=1}^{p}\ln{\tilde{\lambda }}_{3j}+\frac{n}{2}\sum_{j=1}^{p}\left(\frac{{\tilde{\lambda }}_{1j}^{2}}{{\tilde{\lambda }}_{2}}+\frac{{\tilde{\lambda }}_{2}}{{\tilde{\lambda }}_{3j}}\right)+\sum_{j=1}^{p}\frac{{\tilde{\lambda }}_{4j}-1}{{\tilde{\lambda }}_{3j}{\tilde{\lambda }}_{5j}}-\frac{1}{2}\sum_{j=1}^{p}\psi_{0}\left({\tilde{\lambda }}_{4j}-1\right)+\frac{1}{2}\sum_{j=1}^{p}\ln{\tilde{\lambda }}_{5j}. \end{array}$$

The gradient of $KL(\tilde{\Lambda })$ is:

$$\begin{array}{cc} \nabla KL(\tilde{\Lambda })= & \left(n\frac{{\tilde{\lambda }}_{1}}{{\tilde{\lambda }}_{2}},\;-\frac{n}{2}\sum_{j=1}^{p}\left(\frac{{\tilde{\lambda }}_{1j}^{2}}{{\tilde{\lambda }}_{2}^{2}}-\frac{1}{{\tilde{\lambda }}_{3j}}\right),\;\frac{1}{2{\tilde{\lambda }}_{3}}-\frac{n{\tilde{\lambda }}_{2}}{2{\tilde{\lambda }}_{3}^{2}}-\frac{{\tilde{\lambda }}_{4}-1}{{\tilde{\lambda }}_{3}^{2}{\tilde{\lambda }}_{5}},\right.\\ \; & \left.\;\frac{1}{{\tilde{\lambda }}_{3}{\tilde{\lambda }}_{5}}-\frac{1}{2}\psi_{1}\left({\tilde{\lambda }}_{4}-1\right),\;-\frac{{\tilde{\lambda }}_{4}-1}{{\tilde{\lambda }}_{3}{\tilde{\lambda }}_{5}^{2}}+\frac{1}{2{\tilde{\lambda }}_{5}}\right). \end{array}$$

We substitute $\tilde{\theta }=\left({\tilde{\mu }}_{g},\tilde{v_{\epsilon }},{\tilde{v}}_{\tilde{f}},\tilde{\alpha },\tilde{\beta }\right)$ for $\tilde{\Lambda }$ in the $\nabla KL(\tilde{\Lambda })$:

$$\begin{array}{cc} \nabla KL(\tilde{\theta })= & \left(n{\tilde{\mu }}_{g},\;-\frac{n}{2}\sum_{j=1}^{p}\left({\tilde{\mu }}_{g_{j}}^{2}-{\tilde{v}}_{{\tilde{f}}_{j}}\right),\;\frac{1}{2}{\tilde{v}}_{\tilde{f}}-\frac{n{\tilde{v}}_{\tilde{f}}^{2}}{2\tilde{v_{\epsilon }}}-\frac{\tilde{\alpha }{\tilde{v}}_{\tilde{f}}^{2}}{\tilde{\beta }},\right.\\ \; & \left.\;-\frac{1}{2}\psi_{1}\left(\tilde{\alpha }\right)+\frac{{\tilde{v}}_{\tilde{f}}}{\tilde{\beta }},\;\frac{1}{2}{\tilde{\beta }}^{-1}-\frac{\tilde{\alpha }{\tilde{v}}_{\tilde{f}}}{{\tilde{\beta }}^{2}}\right). \end{array}$$

For this algorithm, we fix ${\tilde{\mu }}_{g}^{\left(k+1\right)}=\left(1-n\right){\tilde{\mu }}_{g}$ and repeat for other remaining parameters.

4. The gradient-based algorithm concerning natural parameters:

$$\begin{array}{ccc} {\tilde{v_{\epsilon }}}^{\left(k+1\right)} & = & {\tilde{v_{\epsilon }}}^{\left(k\right)}+\frac{n}{2}\sum_{j=1}^{p}\left({\tilde{\mu }}_{g_{j}}^{2}-{\tilde{v}}_{{\tilde{f}}_{j}}^{\left(k\right)}\right),\\ {\tilde{v}}_{\tilde{f}}^{\left(k+1\right)} & = & \frac{1}{2}{\tilde{v}}_{\tilde{f}}^{\left(k\right)}+\frac{n{\left[{\tilde{v}}_{\tilde{f}}^{\left(k\right)}\right]}^{2}}{2{\tilde{v_{\epsilon }}}^{\left(k+1\right)}}+\frac{{\tilde{\alpha }}^{\left(k\right)}{\left[{\tilde{v}}_{\tilde{f}}^{\left(k\right)}\right]}^{2}}{{\tilde{\beta }}^{\left(k\right)}},\\ {\tilde{\alpha }}^{\left(k+1\right)} & = & {\tilde{\alpha }}^{\left(k\right)}+\frac{1}{2}\psi_{1}\left({\tilde{\alpha }}^{\left(k\right)}\right)-\frac{{\tilde{v}}_{\tilde{f}}^{\left(k+1\right)}}{{\tilde{\beta }}^{\left(k\right)}},\\ {\tilde{\beta }}^{\left(k+1\right)} & = & {\tilde{\beta }}^{\left(k\right)}-\frac{1}{2}{\left[{\tilde{\beta }}^{\left(k\right)}\right]}^{-1}+\frac{{\tilde{\alpha }}^{\left(k+1\right)}{\tilde{v}}_{\tilde{f}}^{\left(k+1\right)}}{{\left[{\tilde{\beta }}^{\left(k\right)}\right]}^{2}}. \end{array}$$
